# Design, synthesis, molecular docking study, and α-glucosidase inhibitory evaluation of novel hydrazide–hydrazone derivatives of 3,4-dihydroxyphenylacetic acid

**DOI:** 10.1038/s41598-024-62034-x

**Published:** 2024-05-18

**Authors:** Hammad Khan, Faheem Jan, Abdul Shakoor, Ajmal Khan, Abdullah F. AlAsmari, Fawaz Alasmari, Saeed Ullah, Ahmed Al-Harrasi, Momin Khan, Shaukat Ali

**Affiliations:** 1 Organic Synthesis and Catalysis Research Laboratory, Institute of Chemical Sciences, University of Peshawar, Peshawar, 25120 Khyber Pakhtunkhwa Pakistan; 2 Shenyang National Laboratory for Materials Science, Institute of Metal Research Chinese Academy of Sciences, Shenyang, 110016 Liaoning China; 3 School of Materials Science and Engineering, University of Science and Technology of China, Shenyang, 110016 Liaoning China; 4 Department of Chemistry, Abdul Wali Khan University, Mardan, 23200 Pakistan; 5 Natural and Medical Sciences Research Center, University of Nizwa, PO Box 33, 616 Birkat Al Mauz, Nizwa, Oman; 6 Department of Pharmacology and Toxicology, College of Pharmacy, King Saud University, Riyadh, 11451 Saudi Arabia

**Keywords:** Biochemistry, Chemical biology, Drug discovery, Chemistry

## Abstract

A series of novel Schiff base derivatives **(1–28)** of 3,4-dihydroxyphenylacetic acid were synthesized in a multi-step reaction. All the synthesized Schiff bases were obtained in high yields and their structures were determined by ^1^HNMR, ^13^CNMR, and HR-ESI–MS spectroscopy. Except for compounds **22**, **26**, **27,** and **28,** all derivatives show excellent to moderate α-glucosidase inhibition. Compounds **5** (IC_50_ = 12.84 ± 0.52 µM), **4** (IC_50_ = 13.64 ± 0.58 µM), **12** (IC_50_ = 15.73 ± 0.71 µM), **13** (IC_50_ = 16.62 ± 0.47 µM), **15** (IC_50_ = 17.40 ± 0.74 µM), **3** (IC_50_ = 18.45 ± 1.21 µM), **7** (IC_50_ = 19.68 ± 0.82 µM), and **2** (IC_50_ = 20.35 ± 1.27 µM) shows outstanding inhibition as compared to standard acarbose (IC_50_ = 873.34 ± 1.67 µM). Furthermore, a docking study was performed to find out the interaction between the enzyme and the most active compounds. With this research work, 3,4-dihydroxyphenylacetic acid Schiff base derivatives have been introduced as a potential class of α-glucosidase inhibitors that have remained elusive till now.

## Introduction

Diabetes mellitus is a persistent and potentially life-threatening metabolic condition characterized by inadequate insulin secretion, resulting in a complication known as hyperglycaemia^1–4^. In type II diabetes mellitus, linked to increased postprandial glucose levels, there is an elevated risk of stroke, atherosclerosis, and other cardiovascular conditions^5,6^. Therefore, an effective approach to managing type II diabetes mellitus and its associated issues involves the inhibition of digestive enzymes, specifically aimed at alleviating postprandial hyperglycemia^7–9^. As enzyme activity and blood glucose concentrations are strongly correlated with each other, the inhibition of α-glucosidase can potentially decrease postprandial blood glucose levels^10,11^. The function of α-glucosidase inhibitors like acarbose, miglitol, and voglibose, used in clinical settings is to slow down sharp rises in blood sugar levels^12,13^. The α-glucosidase enzyme found in human intestinal cells serves a crucial role as the primary hydrolase enzyme. Its primary function involves breaking down complex carbohydrates into glucose monomers, which can then readily diffuse into the bloodstream^14,15^. In the case of type II diabetes, where human body is resistant to insulin then α-glucosidase inhibitors utilization plays a beneficial role in reducing postprandial hyperglycaemia^16,17^. Commonly available α-glucosidase inhibitors such as acarbose, miglitol, and voglibose are associated with side effects such as diarrhea, nausea, and other intestinal disturbances^18,19^. Notably, numerous pieces of evidences indicate that α-glucosidase inhibitors may prove beneficial in the treatment of carbohydrate-mediated diseases, including conditions such as cancer, Alzheimer’s disease, hepatitis, and bacterial and viral infections^20–22^. Consequently use of α-glucosidase inhibitors has emerged as a promising therapeutic avenue for mitigating the risks associated with diabetes and related conditions^23,24^ . Therefore, the pursuit of designing and developing novel α-glucosidase inhibitors with high efficacy and minimal side effects holds a great appeal for medicinal chemists.

**Figure 1:**
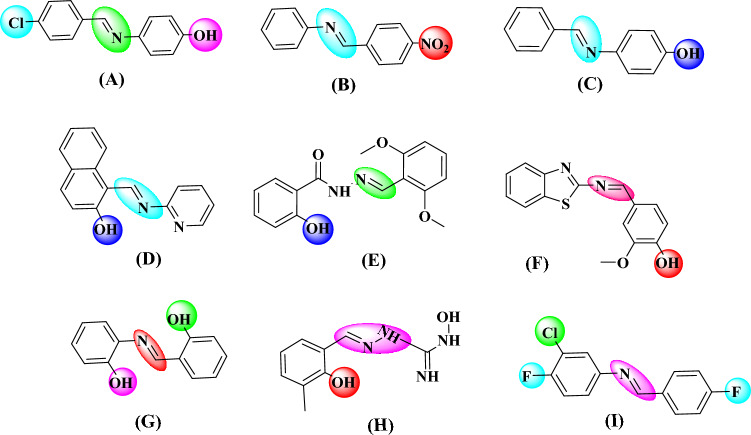
Commercially available drugs containing Schiff base moities.

Schiff bases are one of the most widely used organic compounds having a broad range of applications in different fields such as biology, medicinal drugs, organometallic chemistry, inorganic and analytical chemistry^25–32^. Imine or azomethine groups are mostly present in natural and-synthetic compounds^33–35^. The presence of imine or azomethine group in Schiff bases has shown profound biological importance and have been recognized as privileged precursors for designing biologically active drugs^36–38^ with a broad spectrum of biological activities^39^. For instance compounds (**A**) and (**B**) show antibacterial activity^40–42^, compound (**C**) and (**D**) have anticancer activity^43,44^, compound (**E**) shows anti-cholinesterase activity^45^, compound (**F**) shows anti-inflammatory activity^46^, compound (**G**) shows anti-fungal activity^47^, compound (**H**) shows anti-viral activity^48^, and compound (**I**) shows anti-oxidant activity^49^ **(**Fig. 1**)**. Knowing the biological importance of Schiff bases, this study focuses on synthesizing novel Schiff base derivatives as potent inhibitors of α-glucosidase. These synthetic compounds are expected to possess high lipophilicity, enhanced activity, and facilitating easy passage through cellular barriers.

Most of the synthetic anti-diabetic drugs are widely used now days because they are synthesized from cheaper reagent and are effective and safer than most of natural anti-diabetic drugs. On the basis of literature survey, the current research work is focused mainly on the synthesis of 3,4-dihydroxyphenylacetic acid derived Schiff bases, their molecular docking study and their α-glucosidase inhibition assay.

**Scheme 1.**
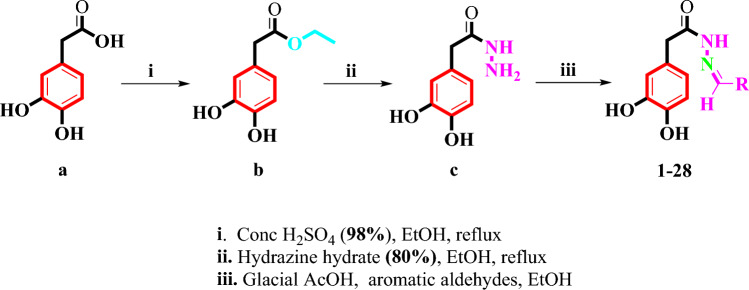
Design for the synthesis of Schiff bases of 3,4-dihydroxyphenylhydrazide.

In the current work a series of 3,4-dihydroxyphenylacetic acid Schiff bases **(1–28)** were synthesized in good yields through multi-step reactions (Scheme 1). First 3,4-dihydroxyphenylacetic acid **(a)** was esterified to the corresponding esters **(b)** in ethanol and a small amount of sulfuric acid and then treated with excess amount of hydrazine hydrate to afford 3,4-dihydroxyphenylhydrazide **(c)**. Subsequently, Schiff bases of 3,4-dihydroxyphenylhydrazide were obtained by treating **c** with various aromatic aldehydes in ethanol and catalytic amount of glacial acetic acid. All the Schiff bases were recrystallized from ethanol in good yields **(1–28)**. The synthesized Schiff bases were characterized with the help of ^1^H-NMR, ^13^C-NMR, and HR-ESI–MS spectroscopy. Finally, Schiff bases of 3,4-dihydroxyphenylhydrazide were subjected to α-glucosidase inhibition assay.

## Materials and methods

3,4-Dihydroxyphenylacetic acid, hydrazine hydrate, ethanol, DMSO, potassium carbonate, and iodine were obtained from Macklin Company and used without further purification. Reactions were tracked using Merck aluminum TLC plates with 0.2 mm of silica gel 60 F-254. The ^1^H-NMR and ^13^C-NMR spectra of the synthesized compounds were recorded in DMSO-d_6_ using an Avance Bruker AM 600 MHz spectrometer. Chemical shift values are expressed in ppm, and coupling constants (J) are reported in hertz (Hz). The molecular masses of the synthesized compounds were determined by high-resolution electrospray ionization mass spectrometry (HR-ESI–MS). Melting points of all the new synthesized compounds were determined using a Digital Electro-thermal apparatus.

### Synthesis of ethyl 2-(2,4-dihydroxyphenyl)acetate (b)

In a 100 mL round-bottom flask, 3,4-dihydroxyphenylacetic acid (**a**) (1.68 g, 10 mmol) was dissolved in 35 mL of absolute ethanol. The temperature of this solution was lowered to 0 °C using an ethanol–water ice bath. Subsequently, 0.5 mL of concentrated sulfuric acid (H_2_SO_4_) was added drop wise as a dehydrating agent, and the mixture was stirred at room temperature for 2 h before being refluxed for an additional 8 h. After cooling the reaction mixture to room temperature, it was poured onto a beaker containing 20 mL of crushed ice. The esterified product (monitored via TLC) was collected through filtration and neutralized with a 5% aqueous solution of sodium bicarbonate (NaHCO_3_), yielding 1.79 g of ethyl 2-(3,4-dihydroxyphenyl)acetate (**b**).

### Synthesis of 2-(3,4-dihydroxyphenyl)acetohydrazide (c)

Ethyl 2-(3,4-dihydroxyphenyl)acetate (**b**) (0.98 g, 5 mmol) was dissolved in 10 mL of absolute ethanol, and 15 mmol of 80% hydrazine hydrate was gradually added. The reaction mixture was refluxed for 8–10 h, monitored by TLC using a solvent system of n-hexane and ethyl acetate (1:2). Upon completion of the reaction, the mixture was cooled to room temperature, resulting in the appearance of a precipitate. The precipitate was filtered, washed with an excess of distilled water (100 mL), and then dried in an oven set at 50 °C, yielding 0.87 g of the desired product (**c**).

### Synthesis of Schiff bases (1–28)

One millimole of 3,4-dihydroxyphenylacetic acid hydrazide was subjected to a reaction with an equimolar amount of aromatic aldehyde in the presence of 5 mL of absolute ethanol as a solvent and a few drops of glacial acetic acid as catalyst. The reaction mixture was refluxed and allowed to stir overnight, with monitoring through TLC (ethyl acetate: n-hexane: methanol 1:1:0.5). The resulting precipitate was cooled to room temperature, filtered, dried, and subsequent recrystallizion from ethanol afforded Schiff bases (**1–28**) . The structural characterization of the synthesized Schiff bases was conducted through ^1^H-NMR, ^13^C-NMR, and HR-ESI–MS spectroscopy.

## Results and discussions

### In vitro α-glucosidase inhibition effect

All synthetic novel Schiff bases of 3,4-dihydroxypehnylacetic acid derivatives **(1–28)** were subjected to α-glucosidase inhibition effect according to the literature protocol^50^. Except compounds **22**, **26**, **27**, and **28**, all derivatives show good to moderate α-glucosidase inhibition potential ranging from (IC_50_ = 12.84 ± 0.52–43.76 ± 2.34 µM) as compared to standard acarbose (IC_50_ = 873.34 ± 1.67 µM). Compounds, **12** (IC_50_ = 15.73 ± 0.71 µM), **15** (IC_50_ = 17.40 ± 0.74 µM), **7** (IC_50_ = 19.68 ± 0.82 µM), **4** (IC_50_ = 13.64 ± 0.58 µM), **13** (IC_50_ = 16.62 ± 0.47 µM), **2** (IC_50_ = 20.35 ± 1.27 µM), **3** (IC_50_ = 18.45 ± 1.21 µM) and **5** (IC_50_ = 12.84 ± 0.52 µM) were found to be more potent among the series Table 1.

**Table 1:** Results for different substituent attached to the products **(1–28).**

Comp. No	R	IC_50_ ± SEM (µM)	Comp. No	R	IC_50_ ± SEM (µM)
1		24.37 ± 0.83	**16**		26.41 ± 1.10
2		20.35 ± 1.27	**17**		43.76 ± 2.34
3		18.45 ± 1.21	**18**		28.16 ± 1.09
4		13.64 ± 0.58	**19**		31.22 ± 1.40
5		12.84 ± 0.52	**20**		27.26 ± 0.83
6		23.69 ± 1.11	**21**		24.16 ± 0.52
7		19.68 ± 0.82	**22**		NA
8		20.88 ± 0.53	**23**		30.46 ± 1.28
9		27.29 ± 1.15	**24**		29.67 ± 0.98
10		28.66 ± 1.21	**25**		34.46 ± 1.48
11		33.51 ± 1.22	**26**		NA
12		15.73 ± 0.71	**27**		NA
13		16.62 ± 0.47	**28**		NA
14		20.61 ± 0.59	Acarbose	Standard inhibitor	873.34 ± 1.67
15		17.405 ± 0.74			

### Structure–activity relationship (SAR)

**Figure 2:**
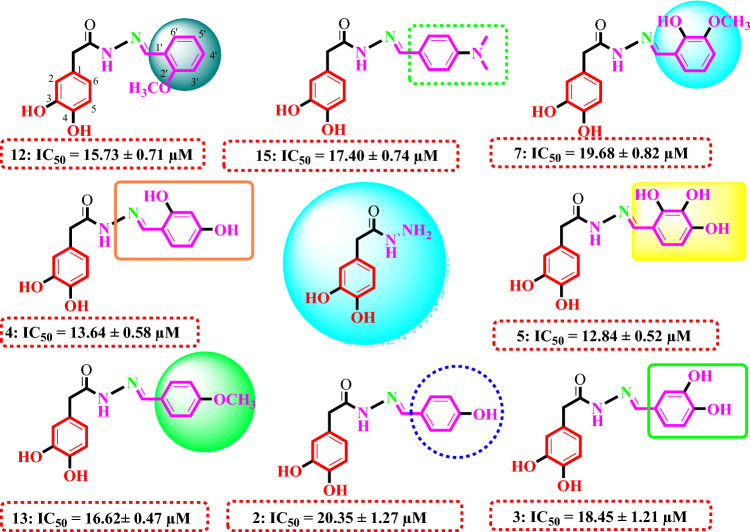
Structure Activity relationship of compounds **2, 3, 4, 5, 7, 12, 13,** and** 15.**

Structure activity relation for α-glucosidase inhibition potential was developed from Table 1 by analyzing the effect of various aldehyde substituents, R and data of inhibition of the most active compounds as shown in Fig. 2. α-glucosidase inhibition activity of hydroxyl-substituted analogs (**1–5**) provides valuable insights into structure–activity relationships (SAR), with a focus on the positional effect of hydroxy groups. All derivatives showed excellent inhibition effect, among the analogs tested, the ortho-hydroxy analog **1** exhibited an excellent inhibition activity, with an IC_50_ value of 24.37 ± 0.83, indicating a potential role for hydroxyl substitution at ortho position. Similarly, the para-hydroxy analog **2** demonstrated comparable activity, with an IC_50_ value of 20.35 ± 1.27, suggesting that hydroxyl substitution at para position also contributes to inhibition potency. The introduction of a second hydroxyl group at positions meta and para position of compound **3** yielded a slightly increased inhibition activity, with an IC_50_ value of 18.45 ± 1.21, highlighting the importance of the proximity and arrangement of hydroxyl groups. Further examination of another ortho, para dihydroxy analog **4** revealed a notable augmentation in inhibition activity, with an IC_50_ value of 13.64 ± 0.58. Most notably, the ortho, meta, and para trihydroxy analog exhibited the highest inhibition activity among the hydroxyl substituted analogs tested, with an IC_50_ value of 12.84 ± 0.52. However, it is crucial to note that all analogs displayed excellent inhibition activity compared to the standard inhibitor, acarbose, which had an IC_50_ value of 873.34 ± 1.67.

Compounds **6–10** represent analogs where the hydrogen of one hydroxyl group has been substituted with either methoxy (compounds **6**, **7**, and **8**) or ethoxy (compounds **9** and **10**). The IC_50_ values for these analogs, namely **6** (IC_50_ = 23.69 ± 1.11),** 7** (IC_50_ = 19.68 ± 0.82), **8** (IC_50_ = 20.88 ± 0.53), **9** (IC_50_ = 27.29 ± 1.15), and **10** (IC_50_ = 28.66 ± 1.21), indicate that the introduction of these electron-donating groups results in a slight decrease in inhibition activity. Moreover, the impact of the ethoxy group appears to be more pronounced than that of the methoxy group, as evidenced by the observed results. Compound **11**, featuring a hydroxyl group at the meta position and a methyl group at the para position, exhibited an IC_50_ value of 33.51 ± 1.22, indicating a decrease in activity when compared to compounds **1–10**.

Compound **12**, with an IC_50_ of 15.73 ± 0.71, is ortho-methoxy substituted, while compound **13**, with an IC_50_ of 16.62 ± 0.47, is para-methoxy substituted. Compound **14**, an analog with meta, para-dimethoxy substitution, exhibits an IC_50_ of 20.61 ± 0.59. Additionally, Compound **15**, featuring a para *N,N*-dimethylamino substituent, displays an IC_50_ of 17.405 ± 0.74, while compound **16** (IC_50_ of 26.41 ± 1.10) and **17** (IC_50_ of 43.76 ± 2.34) with a methyl group at the para position and naphthalene respectively. The activity trends observed among these analogs underscore the significant role that both the position and nature of the substituent play in modulating inhibition activity.

Analogs **18–22** represent chloro-substituted variations. Analog **18** (ortho-chloro) exhibits an IC_50_ value of 28.16 ± 1.09, analog **19** (para-chloro) shows an IC_50_ value of 31.22 ± 1.40, and analog **20** (meta-chloro) demonstrates an IC_50_ value of 27.26 ± 0.83. These analogs, each singly substituted with a chloro group, display no significant variation in activity based on the position of the chloro group. However, compound **21,** ortho meta dichloro-substituted, displays an enhanced activity with an IC_50_ value of 24.16 ± 0.52, compared to its singly-substituted counterpart. Conversely, compound **22** ortho ortho dichloro-substituted, exhibits no inhibitory activity, indicating an extreme positional effect.

Analogs **23** (with an IC_50_ value of 30.46 ± 1.28), **24** (with an IC_50_ value of 29.67 ± 0.98), and **25** (with an IC_50_ value of 34.46 ± 1.48) represent para, ortho, and meta nitro-substituted variations, respectively. The inhibitory results of these analogs also suggest that the impact of the nitro group's position is relatively minor. Conversely, compounds **26–28** exhibited less than 50% inhibition, rendering them inactive.

In summary, the fundamental structure within this class of compounds predominantly dictates their activity. However, the specific characteristics of substituents within the structure exert a notable influence; for instance, hydroxyl substitutions, as well as those with electron-donating or electron-withdrawing properties, can either enhance or diminish the inhibitory activity. Moreover, introducing disubstitutions tends to amplify the effect on inhibition activity. Nonetheless, the positional arrangement of substituents appears to exert a relatively minor influence on inhibition activity.

### Molecular docking

The docking study was carried out using the AutoDock Vina package (1.5.7 version)^51^. The docking study provides deep insight into synthesized compounds’ binding modes with α-glucosidase. The protein structure of α-glucosidase was obtained from Protein Data Bank [https://www.rcsb.org/structure/3WY1]. The water molecules were eliminated from the protein structure, and the missing hydrogens were added to the protein structure using AutoDock Tools. For the docking calculations, a cube grid box was prepared with dimensions of 3.391, 1.310, and − 8.362 Å in *x, y,* and *z*-directions. Furthermore, the grid box position was centered on the middle of compounds under docking. The AutoGrid Tools was used to form grid maps, and spacing within the grid was selected as 0.375 Å. The grid consists of 40 points along *x, y,* and *z*-directions. We focused on the amino acids that play a key role in the binding with the active site of targeted compounds, as we listed in Table 2.

**Table 2:** Different types of interaction with binding affinity.

Compds	Binding affinity (kcal/mol)	Types of interactions		
		Conventional H-bonds	Van der Waals	π*-interactions
**2**	− 7.9	HIS.348, ARG.437, ASN.447, ASP.441, ALA.451	THR.445, GLY.438, LEU.433, PHE.455, GLU.432, ALA.514, VAL.513, ASN.443, THR.448	ARG.450, ALA.454
**12**	− 7.5	THR.445, LYS.352, ASN.44	ALA.45, ALA.451, LEU.433, ASN447, SER.44	ASP.441, ARG.450, ALA.444, HIS.348
**14**	− 8.2	ASP.441	VAL.513, ALA.514, GLU.432, LEU.433, THR.445, HIS.348, ASN.46, SER.44, PHE.455, SER.458, GLN.438, ALA.444, ASN.443, GLN.439	ARG.450, ARG.437, ALA.451, ALA.454
**15**	− 8.0	THR.445, GLU.432, GLU.438	LEU.446, LEU.433, LYS.352, ALA.514, ASN.443, ARG.437, ASN.46, ARG.450, SER.44	HIS.348, ASP.441, ALA.444, ALA.451, ALA.454

**Figure 3:**
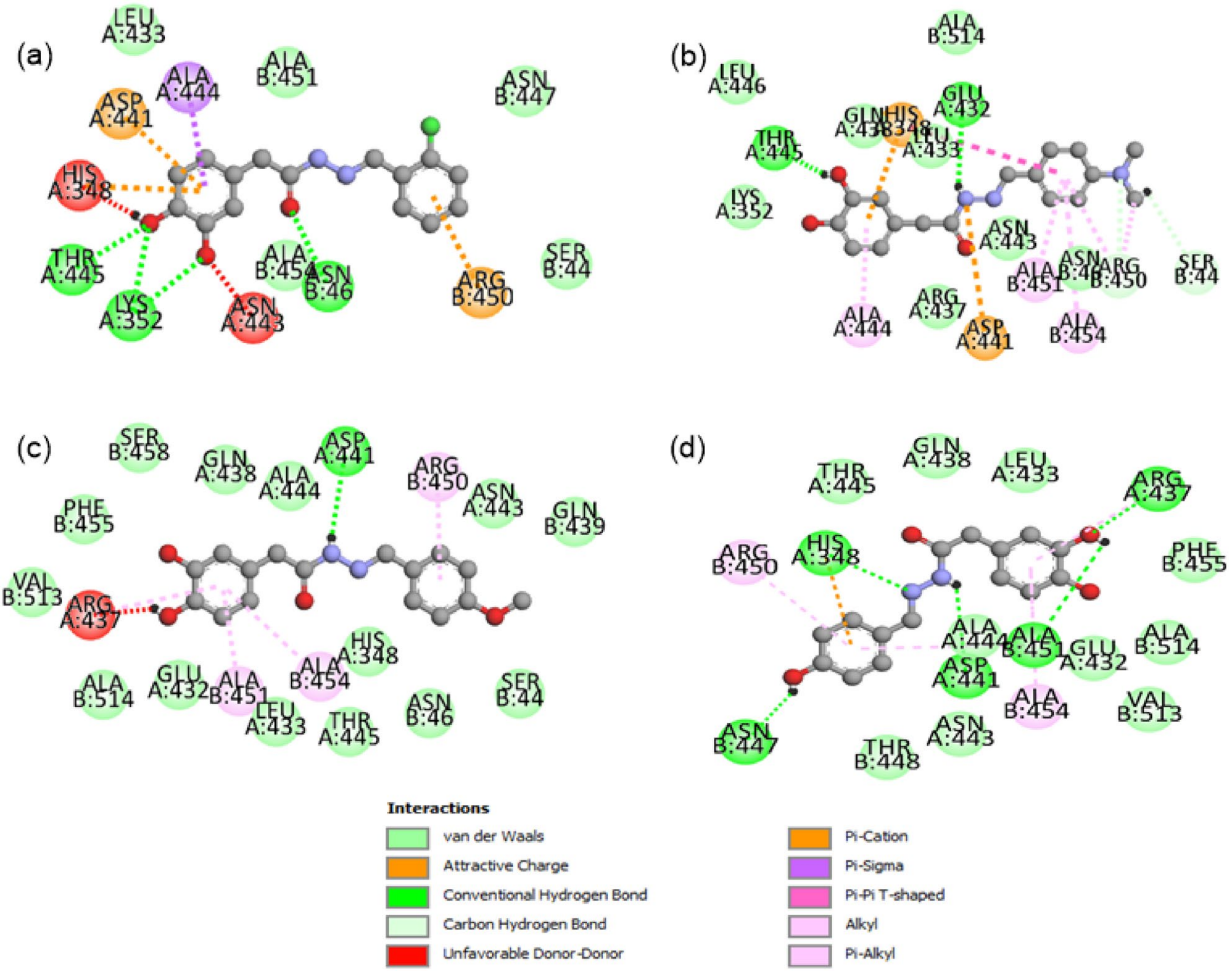
Docking studies of synthesized compounds **12, 15, 13,** and **2**, respectively.

The ligands (compounds) were made and optimized using the density functional theory (DFT) method. After the optimization, the ligands were used for docking calculation. The interactions between the ligands and amino acids of protein were visualized by the PyMOL 2.5.4. The visualization clearly shows the different types of interaction, such as van der Waals, attractive charges, conventional H-bonding, unfavorable interaction, and different types of π-interactions as shown in Fig. 3. The Biovia Discovery Studio was used for making the 2D representation of the interaction between protein and ligand.

The molecular docking analysis reveals complex interactions between compound **12** and various amino acid residues. In particular, THR.445, LYS.352, and ASN.46 engage in conventional hydrogen bonding with the oxo groups of the compound. On the other hand, ALA.45, ALA.451, LEU.433, ASN.447, and SER.44 form van der Waals interactions with the ligand. Additionally, HIS.348, ASP.441, and ARG.450 exhibit π-interactions with the ring's delocalized electronic cloud, as shown in Table 2 and Fig. 3. Furthermore, ALA.444 reveals π-sigma bonding with the aromatic ring. Notably, HIS.348 and ASN.443 contribute to unfavorable interactions with the ligand's oxo groups. The overall binding affinity of compound **12** is calculated as − 7.5 kcal/mol.

Compound **15**, shows a higher binding affinity of − 8.0 kcal/mol. This higher affinity reflects a substantial increase in the number of amino acid residues engaged in the interaction. Noteworthy contributors include LEU.446, LEU.433, LYS.352, ALA.514, and ASN.443, indicating their significant involvement through van der Waals interactions. Key amino acids, namely THR.445, LYS.352, and ASN.46, establish conventional hydrogen bonds with the OH and NH groups. The HIS.348 and ASP.441 participate in π-electronic interactions with the aromatic ring and NH through attractive charges/π-cation interactions, as shown in Fig. 3. Furthermore, ALA.444, ALA.451, and ALA.454 contribute through π-alkyl interactions.

Compound **13** exhibits the highest binding affinity at − 8.2 kcal/mol. Notably, conventional hydrogen bonding is reduced, with only ASP.441 forming such bonds with the NH group of synthesized compound. In contrast, van der Waals interactions significantly increase compared to other compounds, with noteworthy contributions from amino acids such as VAL.513, ALA.514, GLU.432, LEU.433, and others, as listed in Table 2. Furthermore, the ARG.450, ARG.437, ALA.451, and ALA.454 engage in π-interactions with the aromatic ring. These interactions highlight the unique binding characteristics of Compound **13**.

Compound **2** shows the binding affinity as − 7.9 kcal/mol. Notably, in Compound **2**, an increased number of amino acids, including HIS.348, ARG.437, ASN.447, ASP.441, and ALA.451, actively contribute through conventional H-bonding. The engagement of oxygen and nitrogen atoms from compound **2** in H-bonding is shown in Fig. 3. The multifaceted role of HIS.348 not only participates in conventional hydrogen bonding but also engages in π-cation interactions with the aromatic ring. Furthermore, Compound **2** is surrounded by many amino acids through van der Waals interactions, as detailed in Table 2. Additionally, ARG.450 and ALA.454 contribute through π-interactions. After an in-depth molecular docking analysis, compounds **12, 15, 13,** and **2** exhibited diverse interaction profiles with the target protein. These findings underscore their unique binding characteristics, providing valuable insights for their potential development as promising drug discovery and optimization candidates.

## Conclusion

A series of novel Schiff base derivatives (**1–28**) of 3,4-dihydroxyphenylacetic acid were successfully synthesized and characterized using advanced spectroscopic techniques, including ^1^H-NMR, ^13^C-NMR, and high-resolution electrospray ionization mass spectrometry (HR-ESI–MS). Subsequently, the inhibitory potential of these compounds against the α-glucosidase enzyme was evaluated. Most of our synthesized compounds exhibited satisfactory to moderate inhibitory activity against α-glucosidase (see Supplementary information). Notably, derivatives **12**, **15**, **7**, **4**, **13**, **2**, **3**, and **5** demonstrated outstanding inhibition, showcasing IC_50_ values ranging between 12.84 ± 0.52 µM and 20.35 ± 1.27 µM. To further elucidate the binding modes of these promising derivatives with the enzyme active site, molecular docking studies were conducted. This comprehensive approach provides valuable insights into the synthesized compounds' inhibitory potential and their potential application in developing α-glucosidase inhibitors.

### Supplementary Information

Supplementary Information.

## Data Availability

All data generated or analysed during this study are included in this published article and its supplementary information files.
