# Supplementary material for: Design, synthesis, molecular docking study, and α-glucosidase inhibitory evaluation of novel hydrazide–hydrazone derivatives of 3,4-dihydroxyphenylacetic acid
Source: Sci Rep. 2024 May 18;14:11410. doi: 10.1038/s41598-024-62034-x (PMC11102520; doi:10.1038/s41598-024-62034-x)
Supplement: Supplementary file 1 — Supplementary Information. [file 41598_2024_62034_MOESM1_ESM.docx]

**Supporting Information**

**Design, Synthesis, Molecular Docking Study, and α-glucosidase Inhibitory Evaluation of Novel Hydrazide-Hydrazone Derivatives of 3,4-Dihydroxyphenylacetic Acid**

Hammad Khan^a^, Faheem Jan^b,c^, Abdul Shakoor^d^, Ajmal Khan^e^, Abdullah F. AlAsmari^f^, Fawaz Alasmari^f^, Saeed Ullah^e^, Ahmed Al-Harrasi^e^*, Momin Khan^d^*, Shaukat Ali^a^*

^a^Organic Synthesis and Catalysis Research Laboratory, Institute of Chemical Sciences, University of Peshawar, Peshawar 25120, Khyber Pakhtunkhwa, Pakistan.

^b^Shenyang national laboratory for Materials Science, Institute of Metal Research Chineses Academy of Sciences, Shenyang, 110016, Liaoning, China

^c^School of Materials Science and Engineering, University of Science and Technology of China, Shenyang 110016, Liaoning, China

^d^Department of Chemistry, Abdul Wali Khan University, Mardan-23200, Pakistan

^e^Natural and Medical Sciences Research Center, University of Nizwa, PO Box 33, 616 Birkat Al Mauz, Nizwa, Oman

^f^Department of Pharmacology and Toxicology, College of Pharmacy, King Saud University, Riyadh 11451, Saudi Arabia

**Corresponding Author:** [drshaukatali@uop.edu.pk](mailto:drshaukatali@uop.edu.pk); [mominkhan@awkum.edu.pk](mailto:mominkhan@awkum.edu.pk); [aharrasi@unizwa.edu.om](mailto:aharrasi@unizwa.edu.om);

**Characterization data of compounds (1-28)**

1. **(3,4-dihydroxyphenyl)-*N*'-(2-hydroxybenzylidene)acetohydrazide (1)**

Yield: 0.25 g (82%); (white crystalline). M.P.: 164ºC; ^1^H-NMR (600 MHz, DMSO-d_6,_ *δ*, ppm): 11.63(s, 1H, -NH), 10.75 (s, 1H, H-OH), 9.15 (s, 2H, (-OH)_2_), 8.46 (s, 1H, =CH), 7.48-7.47 (d, 1H, *J* _6’,5’_ = 7.2 Hz, H-6’), 7.21-7.19 (t, 1H, *J* _4’(5’,6’)_ = 7.2, H-4’), 6.91-6.89 (t, 1H, *J* _5’(4’,6’)_ = 7.2 Hz, H-5’), 6.70-6.68 (d, 1H, *J*_3’,4’_ = 7.2 Hz, H-3’), 6.53 (s, 1H, H-2), 6.32-6.31 (d, 1H, *J* _5,6_ = 7.2 Hz, H-5), 6.20-6.18 (d, 1H, *J*_6,5_ = 7.2 H-6), 3.97 (s, 2H,-CH_2_, H-Ph); ^13^C NMR (150 MHz, DMSO‑d_6_): *δ* 171.0, 157.2, 138.3, 138.3, 137.4, 132.8, 132.3, 127.2, 123.4, 121.6, 119.2, 117.7, 116.5, 115.4, 40.6; LC-HRMS (ESI^+^): [M+H]^+^ calcld for C_15_H_14_N_2_O_4_: 286.287; found 287.287.

**2-(3,4-dihydroxyphenyl)-*N*'-(4-hydroxybenzylidene)acetohydrazide (2)**

Yield: 0.23 g (71%); (Pale yellow powder); M.P.: 210ºC; ^1^H-NMR (600 MHz, DMSO-d_6,_ *δ*, ppm): 11.13 (s, 1H, -NH), 9.76 (s, 1H, -OH, H-4’), 9.53 (s, 2H, (-OH)_2,_ H-3/4), 8.46 (s, 1H, =CH), 7.76-7.75 (d, 2H, *J* _2’,3’/6’,5’_ = 7.2 Hz, H-2’/6’). 6.97-6.96 (t, 3H, *J* = 7.2 H-2/3’/5’), 6.73-6.72 (d, 1H, *J* _5,6_ = 7.2 Hz, H-5), 6.61-6.60 (d, 1H, *J* _6,5_ = 6.6 Hz, H-6), 3.92 (s, 2H,-CH_2_, H-Ph); ^13^C NMR (150 MHz, DMSO‑d_6_): *δ* 171.0, 160.2, 146.2, 146.2, 144.7, 132.3, 130.2. 130.2, 126.8, 123.3, 116.8, 116.2, 116.2, 115.6, 40.6; LC-HRMS (ESI^+^): [M+H]^+^ calcld for C_15_H_14_N_2_O_4_: 286.287; found 287.099.

***N*'-(3,4-dihydroxybenzylidene)-2-(3,4-dihydroxyphenyl)acetohydrazide (3)**

Yield: 0.19 g (67%); (Off white powder); M.P.: 212ºC; ^1^H-NMR (600 MHz, DMSO-d_6,_ *δ*, ppm): 11.18 (s, 1H, -NH), 9.54 (s, 4H, (-OH)_4_, H-3/3’/4/4’), 8.45 (s, 1H, =CH), 7.34-7.33 (d, 2H, *J* _2’,4’/6’,5’_ = 7.2 Hz, H-2’/6’), 6.87 (s, 1H, H-2), 6.78-6.77 (d, 1H, *J* _5’,6’_ = 7.2 Hz, H-5’), 6.64-6.63 (d, 1H, *J* _5,6_ = 7.2 Hz, H-5), 6.52-6.51 (d, 1H, *J* _6,5_ = 7.2, H-6), 3.86 (s, 2H, -CH_2_, H-Ph), 2.12 (s, 3H, -CH3, H-4’); ^13^C NMR (150 MHz, DMSO‑d_6_): *δ* 171.4, 156.4, 148.3, 147.5, 147.5, 145.7, 132.6, 132.4, 128.3, 124.2, 122.5, 117.4, 116.2, 115.6, 40.6; LC-HRMS (ESI^+^): [M+H]^+^ calcld for C_15_H_14_N_2_O_5_: 302.286; found 303.094 .

***N*'-(2,4-dihydroxybenzylidene)-2-(3,4-dihydroxyphenyl)acetohydrazide (4)**

Yield: 0.25 g (82%); (Yellow crystalline). M.P.: 198ºC; ^1^H-NMR (600 MHz, DMSO-d_6,_ *δ*, ppm): 11.44 (s, 1H, -NH), 11.14 (s, 1H,-OH, H-2’), 10.11 (s, 1H,-OH, H-4’), 9.53 (s, 2H, (-OH)_2,_ H-3/4), 8.75 (s, 1H, =CH), 7.65-7.54 (m, 2H, H-3’/6’), 6.84 (s, 1H, H-2), 6.68-6.67 (d, 1H, *J* _5,6_ = 7.2, H-5), 6.54-6.53 (d, 1H, *J* _6,5_ = 7.2, H-6), 6.33-6.32 (d, 1H, *J* _5’,6’_ = 7.2 Hz, H-5’), 3.86 (s, 2H,-CH_2_, H-Ph); ^13^C NMR (150 MHz, DMSO‑d_6_): *δ* 171.4, 163.2, 162.1, 148.8, 148.8, 148.2, 133.7, 132.5, 123.6, 116.8, 115.4, 111.5, 108.3, 103.8, 40.6; LC-HRMS (ESI^+^): [M+H]^+^ calcld for C_15_H_14_N_2_O_4_: 302.286; found 303.094.

**2-(3,4-dihydroxyphenyl)-*N*'-(2,3,4-trihydroxybenzylidene)acetohydrazide (5)**

Yield: 0.24 g (78%); (Off white powder). M.P.: 212ºC; ^1^H-NMR (600 MHz, DMSO-d_6,_ *δ*, ppm): 11.38 (s, 1H, -NH), 11.16 (s, 1H,-OH, H-2’), 10.11 (s, 1H,-OH, H-3’), 9.57 (s, 3H, (-OH)_3,_ H-3/4/4’), 8.74 (s, 1H, =CH), 7.65-7.63 (d, 1H, *J* _6’,5’_ = 7.2, H-6’), 6.84 (s, 1H, H-2), 6.68-6.67 (d, 1H, *J* _5,6_ = 7.2, H-5), 6.54-6.53 (d, 1H, *J* _6,5_ = 7.2, H-6), 6.33-6.32 (d, 1H, *J* _5’,6’_ = 7.2 Hz, H-5’), 3.86 (s, 2H,-CH_2_, H-Ph); ^13^C NMR (150 MHz, DMSO‑d_6_): *δ* 171.4, 162.9, 161.1, 148.7, 148.7, 148.2, 133.5, 132.8, 123.3, 116.4, 115.6, 111.7, 108.3, 103.6, 40.6; LC-HRMS (ESI^+^): [M+H]^+^ calcld for C_15_H_14_N_2_O_6_: 318.285; found 319.089.

**2-(3,4-dihydroxyphenyl)-*N*'-(4-hydroxy-3-methoxybenzylidene)acetohydrazide (6)**

Yield: 0.24 g (79%); (Yellow powder). M.P.: 189ºC; ^1^H-NMR (600 MHz, DMSO-d_6,_ *δ*, ppm): 11.21 (s, 1H, -NH), 9.73 (s, 1H, H-OH), 9.67 (s, 2H, (-OH)_2_), 8.58 (s, 1H, =CH), 7.45-7.34 (m, 2H, H-2’/6’), 6.91-6.89 (d, 1H, *J* _5’,6’_ = 6.6 Hz, H-5’), 6.74 (s, 1H, H-2), 6.61-6.59 (d, 1H, *J* _5,6_ = 6.6 Hz, H-5), 6.41-6.39 (d, 1H, *J* _6,5_ = 6.6 Hz, H-6), 3.85 (s, 3H,-OCH_3,_ H-3’), 3.79 (s, 2H,-CH_2_, H-Ph); ^13^C NMR (150 MHz, DMSO‑d_6_): *δ* 171.0, 151.8, 149.4, 147.2, 146.8, 146.8, 132.4, 131.3, 123.3, 122.7, 117.2, 116.2, 115.3, 112.1, 58.2, 40.6; LC-HRMS (ESI^+^): [M+H]^+^ calcld for C_16_H_16_N_2_O_5_: 316.313; found 317.109.

**2-(3,4-dihydroxyphenyl)-*N*'-(2-hydroxy-3-methoxybenzylidene)acetohydrazide (7)**

Yield: 0.28 g (89%); (Pale yellow powder). M.P.: 204ºC; ^1^H-NMR (600 MHz, DMSO-d_6,_ *δ*, ppm): 11.24 (s, 1H, -NH), 9.28 (s, 1H, H-OH), 8.10 (s, 2H, (-OH)_2_), 7.64 (s, 1H, =CH), 6.61-6.59 (d, 1H, *J* _6’,5’_ = 7.2 Hz, H-6’), 6.38-6.37 (d, 1H, *J* _4’,5’_ = 7.2 Hz, H-4’), 6.21-6.14 (m, 2H, H-2/5’), 5.95-5.94 (d, 1H, *J* _5,6_ = 7.2 Hz, H-5), 5.87-5.86 (d, 1H, *J* _6,5_ = 7.2 Hz, H-6), 3.97 (s, 2H, -CH_2_, H-Ph), 3.87 (s, 3H, -OCH3, H-3’); 6^13^C NMR (150 MHz, DMSO‑d_6_): *δ* 171.4, 161.4, 159.3, 146.8, 146.8, 146.2, 143.3, 124.4, 123.3, 119.6, 116.8, 116.3, 115.2, 115.2, 67.2, 40.6; LC-HRMS (ESI^+^): [M+H]^+^ calcld for C_16_H_16_N_2_O_5_: 316.313; found 317.109 .

**2-(3,4-dihydroxyphenyl)-*N*'-(2-hydroxy-4-methoxybenzylidene)acetohydrazide (8)**

Yield: 0.23 g (84%); (Off white powder); M.P.: 206ºC; ^1^H-NMR (600 MHz, DMSO-d_6,_ *δ*, ppm): 11.82 (s, 1H, -NH), 11.13 (s, 1H, H-OH), 9.53 (s, 2H, (-OH)_2_), 8.84 (s, 1H, =CH), 6.84-6.83 (d, 1H, *J* _6’,5’_ = 7.2 Hz, H-6’), 6.81 (s, 1H, H-2), 6.69-6.68 (d, 1H, *J* _5,6_ = 7.2 Hz, H-5), 6.57-6.56 (m, 2H, H-5’/6), 6.49 (s, 1H, H-3’), 3.87(s, 2H, -CH_2_, H-Ph), 3.75 (s, 3H, -OCH3, H-4’); ^13^C NMR (150 MHz, DMSO‑d_6_): *δ* 171.4, 161.4, 159.3, 146.2, 146.2, 145.8, 133.2, 132.8, 123.3, 116.4, 115.2, 110.4, 107.3, 104.5, 55.2, 40.6; LC-HRMS (ESI^+^): [M+H]^+^ calcld for C_16_H_16_N_2_O_5_: 316.313; found 317.109.

**2-(3,4-dihydroxyphenyl)-*N*'-(3-ethoxy-4-hydroxybenzylidene)acetohydrazide (9)**

Yield: 0.26 g (86%); (Pale yellow powder). M.P.: 184ºC; ^1^H-NMR (600 MHz, DMSO-d_6,_ *δ*, ppm): 11.21 (s, 1H, -NH), 9.89 (s, 1H, H-OH), 8.41 (s, 2H, (-OH)_2_), 8.34 (s, 1H, =CH), 7.25-7.14 (m, 2H, H-2’/6’), 6.71-6.69 (d, 1H, *J* _5’,6’_ = 6.6 Hz, H-5’), 6.54 (s, 1H, H-2), 6.41-6.39 (d, 1H, *J* _5,6_ = 6.6 Hz, H-5), 6.21-6.19 (d, 1H, *J* _6,5_ = 6.6 Hz, H-6), 3.97-3.76 (m, 2H, -OCH_2_-, H-3’), 3.56 (s, 2H, -CH_2_, H-Ph), 1.63-1.62 (t, 3H, -CH_3_, *J* = 4.2 Hz, H-3’); 6^13^C NMR (150 MHz, DMSO‑d_6_): *δ* 171.4, 163.8, 162.4, 146.6, 146.6, 145.4, 134.6, 133.5, 122.8, 118.9, 116.7, 110.3, 107.8, 103.9, 65.2, 40.6, 14.7; LC-HRMS (ESI^+^): [M+H]^+^ calcld for C_17_H_18_N_2_O_5_: 330.340; found 331.125.

**2-(3,4-dihydroxyphenyl)-*N*'-(4-ethoxy-2-hydroxybenzylidene)acetohydrazide (10)**

Yield: 0.25 g (82%); (white crystalline); M.P.: 164ºC; ^1^H-NMR (600 MHz, DMSO-d_6,_ *δ*, ppm): 11.51 (s, 1H, -NH), 10.89 (s, 1H, H-OH), 8.84 (s, 2H, (-OH)_2_), 8.24 (s, 1H, =CH), 7.98-7.97 (d, 1H, *J* _6’,5’_ = 7.2 Hz, H-6’), 7.01 (s, 1H, H-2), 6.84-6.83 (d, 1H, *J* _5,6_ = 7.2 Hz, H-5), 6.74-6.73 (d, 1H, *J* _6,5_ = 7.2 Hz, H-6), 6.61 (s, 1H, H-3’), 6.58-6.57 (d, 1H, *J* _5’,6’_ = 7.2 Hz, H-5’), 4.37-4.26 (m, 2H, -OCH_2_-_,_ H-4’), 3.97 (s, 2H,-CH_2_, H-Ph), 1.43-1.42 (t, 3H, -CH_3,_ *J* = 4.2 Hz, H-4’); ^13^C NMR (150 MHz, DMSO‑d_6_): *δ* 171.1, 163.6, 162.8, 146.5, 146.5, 145.9, 134.3, 133.5, 123.5, 118.9, 117.3, 110.1, 108.4, 104.2, 64.2, 40.6, 14.8; LC-HRMS (ESI^+^): [M+H]^+^ calcld for C_17_H_18_N_2_O_5_: 330.340; found 331.125.

**2-(3,4-dihydroxyphenyl)-*N*'-(3-hydroxy-4-methylbenzylidene)acetohydrazide (11)**

Yield: 0.26 g (87%); (Off white powder); M.P.: 210ºC; ^1^H-NMR (600 MHz, DMSO-d_6,_ *δ*, ppm): 11.34 (s, 1H, -NH), 9.87 (s, 1H, -OH, H-3’), 9.74 (s, 2H, (-OH)_2_), 8.65 (s, 1H, =CH), 7.54-7.53 (d, 1H, *J* _6’,5’_ = 7.6 Hz, H-6’), 7.31 (s, 1H, H-2’) 7.24-7.23 (d, 1H, *J* _5’,6’_ = 7.2 Hz, H-5’), 6.84 (s, 1H, H-2), 6.68-6.67 (d, 1H, *J* _5,6_ = 7.2 Hz, H-5), 6.54-6.53 (d, 1H, *J* _6,5_ = 7.2, H-6), 3.86 (s, 2H, -CH_2_, H-Ph), 2.12 (s, 3H, -CH3, H-4’); ^13^C NMR (150 MHz, DMSO‑d_6_): *δ* 171.4, 156.2, 148.4, 147.3, 147.3, 136.7, 132.8, 132.2, 128.2, 124.4, 122.6, 117.2, 116.6, 115.8, 40.6, 15.4; LC-HRMS (ESI^+^): [M+H]^+^ calcld for C_16_H_16_N_2_O_4_: 300.314; found 301.114.

**2-(3,4-dihydroxyphenyl)-*N*'-(2-methoxybenzylidene)acetohydrazide (12)**

Yield: 0.26 g (85%); (Off white powder). M.P.: 190-193ºC; H-NMR (600 MHz, DMSO-d_6,_ *δ*, ppm): 11.21 (s, 1H, -NH), 9.74 (s, 2H, H,(-OH)_2_), 8.84 (s, 1H, =CH), 7.98-7.97 (d, 1H, *J* _6’,5’_ = 7.2 Hz, H-6’), 7.78-7.64 (m, 1H, H-4’), 7.45-7.35 (m, 1H, H-5’), 7.28-7.27 (d, 1H, *J* _3’,4’_ = 7.2, H-3’), 6.93 (s, 1H, H-2) 6.76-6.75 (d, 1H, *J* _5,6_ = 7.2, H-5), 6.68-6.67 (d, 1H, *J* _6,5_ = 7.2, H-6), 3.96 (s, 3H, -OCH_3_, H-2’), 3.87 (s, 2H,-CH_2_, H-Ph); ^13^C NMR (150 MHz, DMSO‑d_6_): *δ* 171.6, 158.4, 146.5, 146.5, 146.1, 132.7, 131.2, 122.8, 121.3, 116.8, 116.3, 115.0, 111.2, 55.7, 40.6; LC-HRMS (ESI^+^):[M+H]^+^ calcld for C_16_H_16_N_2_O_4_: 300.314; found 301.114.

**2-(3,4-dihydroxyphenyl)-*N*'-(4-methoxybenzylidene)acetohydrazide (13)**

Yield: 0.27 g (88%); (Pale yellow powder); M.P.: 198ºC; H-NMR (600 MHz, DMSO-d_6,_ *δ*, ppm): 11.24 (s, 1H, -NH), 9.53 (s, 2H, H,(-OH)_2_), 8.58 (s, 1H, =CH), 7.95-7.94 (d, 2H, *J* _2’,4’/6’,5’_ = 7.2 Hz, H-2’/6’), 7.24-7.23 (d, 2H, *J* _3’,2’/5’,6’_ = 7.2 Hz, H-3’/5’), 6.87 (s, 1H, H-2), 6.74-6.73 (d, 1H, *J* _5,6_ = 7.2, H-5), 6.64-6.63 (d, 1H, *J* _6,5_ = 7.2, H-6), 3.95 (s, 2H,-CH_2_, H-Ph), 3.86 (s, 3H, -CH_3_, H-4’); ^13^C NMR (150 MHz, DMSO‑d_6_): *δ* 171.4, 163.2, 148.4, 148.4, 146.3, 131.6, 130.1, 130.1, 126.7, 124.4, 118.5, 116.9, 116.2, 116.2, 58.6, 40.6; LC-HRMS (ESI^+^): [M+H]^+^ calcld for C_16_H_16_N_2_O_4_: 300.314; found 301.114.

**2-(3,4-dihydroxyphenyl)-*N*'-(3,4-dimethoxybenzylidene)acetohydrazide (14)**

Yield: 0.28 g (89%); (Yellow powder); M.P.: 210ºC; ^1^H-NMR (600 MHz, DMSO-d_6,_ *δ*, ppm): 11.18 (s, 1H, -NH), 9.35 (s, 2H, (-OH)_2_), 8.24 (s, 1H, =CH), 7.34-7.21 (m, 2H, H-2’/6’) 6.71-6.69 (d, 1H, *J* _5’,6’_ = 6.6 Hz, H-5’), 6.41 (s, 1H, H-2), 6.31-6.29 (d, 1H, *J* _5,6_ = 6.6 Hz, H-5), 6.21-6.19 (d, 1H, *J* _6,5_ = 6.6 Hz, H-6), 3.85 (s, 6H, (-OCH_3_)_2,_ H-3’/4’), 3.79 (s, 2H, -CH_2_, H-Ph); ^13^C NMR (150 MHz, DMSO‑d_6_): *δ* 171.0, 151.9, 149.7, 147.2, 146.8, 146.8, 131.4, 130.3, 123.4, 122.3, 117.2, 116.2, 111.3, 109.1, 56.2, 56.2, 40.6; LC-HRMS (ESI^+^): [M+H]^+^ calcld for C_17_H_18_N_2_O_5_: 330.340; found 331.125.

1. **(3,4-dihydroxyphenyl)-*N*'-(4-(dimethylamino)benzylidene)acetohydrazide (15)**

Yield: 0.28 g (85%); (Orange powder). M.P.: 203ºC; ^1^H-NMR (600 MHz, DMSO-d_6,_ *δ*, ppm): 11.11 (s, 1H, -NH), 9.48 (s, 2H, (-OH)_2_), 8.48 (s, 1H, =CH), 7.51-7.49 (d, 2H, *J* _2’.3’/6’, 5’_ = 6.6 H-2’/6’), 6.91 (s, 1H, H-2), 6.81-6.79 (d, 1H, *J* _3’,2’/5’, 6’_ = 6.6 Hz, H-3’/5’), 6.61-6.55 (d, 1H, *J* _5,6_ = 6.6 Hz, H-5), 6.41-6.39 (d, 1H, *J* _6,5_ = 6.6 Hz, H-6), 3.85 (s, 2H,-CH_2_, H-Ph), 3.01 (s, 6H,(-CH_3_)_2_ H-4’); 6^13^C NMR (150 MHz, DMSO‑d_6_): *δ* 171.2, 153.3, 146.4, 146.4, 144.2, 132.5, 128.3, 128.3, 123.3, 117.2, 116.2, 115.3, 112.1, 112.1, 41.5, 41.5, 40.6; LC-HRMS (ESI^+^): [M+H]^+^ calcld for C_17_H_19_N_3_O_3_: 313.357; found 314.146.

**2-(3,4-dihydroxyphenyl)-*N*'-(4-methylbenzylidene)acetohydrazide (16)**

Yield: 0.27 g (82%); (Off white powder); M.P.: 201ºC; ^1^H-NMR (600 MHz, DMSO-d_6,_ *δ*, ppm): 11.18 (s, 1H, -NH), 9.65 (s, 2H, (-OH)_2_), 8.58 (s, 1H, =CH), 8.01-7.99 (d, 1H, *J* _2’,3’/6’,5’_ = 7.2 Hz, H-2’/6’). 7.34-7.33 (d, 2H, *J* _3’,2’/5’,6’_ = 7.2 H-3’/5’), 6.84 (s, 1H, H-2), 6.76-6.75 (d, 1H, *J* _5,6_ = 7.2 Hz, H-5), 6.63-6.52 (d, 1H, *J* _6,5_ = 6.6 Hz, H-6), (d, 1H, *J* _6,6_ = 6.6 Hz, H-6), 2.54 (s, 3H, -CH_3_,H-4’); ^13^C NMR (150 MHz, DMSO‑d_6_): *δ* 171.7, 148.2, 148.2, 146.5, 143.6, 132.2, 130.6. 129.4, 129.4, 127.6, 127.6, 124.3, 118.3, 116.7, 40.6, 21.3; LC-HRMS (ESI^+^): [M+H]^+^ calcld for C_16_H_16_N_2_O_3_: 284.315; found 285.119.

**2-(3,4-dihydroxyphenyl)-*N*'-(naphthalen-1-ylmethylene)acetohydrazide (17)**

Yield: 0.21 g (69%); (Yellow powder); M.P.: 210ºC; ^1^H-NMR (600 MHz, DMSO-d_6,_ *δ*, ppm): 11.23 (s, 1H, -NH), 9.53 (s, 2H, (-OH)_2,_ H-3/4), 8.65-8.64 (d, 1H, *J* _2’,3’_ = 7.2 Hz, H-2’), 8.54-8.43 (m, 2H, H-( =CH)/4’), 8.12-7.94 (m, 3H, H-3’/6’/9’), 7.89-7.87 (t, 1H, *J* _8’(7’,9’)_= 7.2Hz, H-8’), 7.58-7.56 (t, 1H, *J* _7’(6’,8’)_= 7.2Hz, H-7’), 6.91 (s, 1H, H-2), 6.64-6.62 (d, 1H, *J* _6,5_ = 6.6 Hz, H-6), 6.57-6.54 (d, 1H, *J*_5,6_ = 7.2Hz, H-5), 3.92 (s, 2H,-CH_2_, H-Ph); ^13^C NMR (150 MHz, DMSO‑d_6_): *δ* 171.0, 146.2, 146.2, 144.7, 133.6, 132.3. 131.2, 130.1, 128.8, 128.3, 127.3, 126.8, 126.8, 125.4, 123.3, 122.6, 116.8, 115.6, 40.6; LC-HRMS (ESI^+^): [M+H]^+^ calcld for C_19_H_16_N_2_O_3_: 320.348; found 321.119.

***N'*-(2-chlorobenzylidene)-2-(3,4-dihydroxyphenyl)acetohydrazide (18)**

Yield: 0.26 g (83%); (Pale yellow).M.P.: 164ºC; ^1^H-NMR (600 MHz, DMSO-d_6,_ *δ*, ppm): 11.23 (s, 1H, -NH), 9.75 (s, 2H, H,(-OH)_2_), 9.15 (s, 1H, =CH), 7.98-7.97 (d, 1H, *J* _6’5’_ = 7.2 Hz, H-6’), 7.86-7.85 (d, 2H, *J* _3’4’/4’5’_ = 7.2 Hz, H-3’/4’), 7.61-7.43 (m, 1H, H-5’), 6.94 (s, 1H, H-2), 6.73-6.72 (d, 1H, *J* _5,6_ = 7.2, H-5), 6.68-6.67 (d, 1H, *J* _6,5_ = 7.2, H-6), 3.97 (s, 2H,-CH_2_, H-Ph). (s, 2H, H(-CH_2_)); ^13^C NMR (150 MHz, DMSO‑d_6_): *δ* 171.8, 147.8, 147.8, 139.3, 135.4, 134.1, 133.1, 132.4, 130.1, 124.8, 120.3, 118.1, 117.5, 40.6; LC-HRMS (ESI^+^): [M+H]^+^ calcld for C_15_H_13_ClN_2_O_3_: 304.730; found 305.730.

***N*'-(4-chlorobenzylidene)-2-(3,4-dihydroxyphenyl)acetohydrazide (19)**

Yield: 0.26 g (79%); (Yellow powder); M.P.: 198ºC; ^1^H-NMR (600 MHz, DMSO-d_6,_ *δ*, ppm): 11.18 (s, 1H, -NH), 9.72 (s, 2H, (-OH)_2,_ H-3/4), 8.49 (s, 1H, =CH), 7.96-7.95 (d, 2H, *J* _2’,3’/6’,5’_ = 7.2 Hz, H-2’/6’), 7.57-7.56 (d, 2H, *J*_3’,2’/5’, 6’_ = 7.2Hz, H-3’/5’), 6.96 (s, 1H, H-2), 6.73-6.72 (d, 1H, *J* _6,5_ = 6.6 Hz, H-6), 6.61-6.60 (d, 1H, *J*_5,6_ = 7.2Hz, H-5), 3.92 (s, 2H,-CH_2_, H-Ph); ^13^C NMR (150 MHz, DMSO‑d_6_): *δ* 171.4, 146.2, 146.2, 144.7, 139.5, 132.2. 131.2, 130.2, 130.2, 129.8, 129.8, 123.3, 116.2, 115.6, 40.6; LC-HRMS (ESI^+^): [M+H]^+^ calcld for C_15_H_13_ClN_2_O_3_: 304.730; found 305.065 .

***N'*-(3-chlorobenzylidene)-2-(3,4-dihydroxyphenyl)acetohydrazide (20)**

Yield: 0.28 g (79%); (Yellow powder); M.P.: 196ºC; ^1^H-NMR (600 MHz, DMSO-d_6,_ *δ*, ppm): 11.14 (s, 1H, -NH), 9.52 (s, 2H, (-OH)_2,_ H-3/4), 8.46 (s, 1H, =CH), 7.96 (s, 1H, H-2’), 7.85-7.77 (m, 1H, H-6’), 7.66-7.58 (m, 2H, H-4’/5’), 6.96 (s, 1H, H-2), 6.73-6.72 (d, 1H, *J* _5,6_ = 6.6 Hz, H-5), 6.61-6.60 (d, 1H, *J* _6,5_ = 7.2Hz, H-6), 3.92 (s, 2H,-CH_2_, H-Ph); ^13^C NMR (150 MHz, DMSO‑d_6_): *δ* 171.0, 147.4, 146.2, 146.2 144.9, 133.3, 132.2. 131.8, 130.3, 128.8, 127.4, 123.7, 116.8, 115.6, 40.6; LC-HRMS (ESI^+^): [M+H]^+^ calcld for C_15_H_13_ClN_2_O_3_: 304.730; found 305.065. ***N*'-(2,3-dichlorobenzylidene)-2-(3,4-dihydroxyphenyl)acetohydrazide (21)**

Yield: 0.28 g (79%); (Yellow powder); M.P.: 196ºC; ^1^H-NMR (600 MHz, DMSO-d_6,_ *δ*, ppm): 11.10 (s, 1H, -NH), 9.51 (s, 2H, (-OH)_2,_ H-3/4), 9.02 (s, 1H, =CH), 7.68-7.67 (s, 1H, *J* _6’,5’_ = 7.2Hz, H-6’), 7.51-7.42 (m, 2H, H-4’/5’), 6.89 (s, 1H, H-2), 6.64-6.63 (d, 1H, *J* _5,6_ = 7.2Hz, H-5), 6.54-6.53 (d, 1H, *J* _6,5_ = 7.2Hz, H-6), 3.81 (s, 2H,-CH_2_, H-Ph); ^13^C NMR (150 MHz, DMSO‑d_6_): *δ* 171.4, 146.7, 146.7, 142.7, 139.4, 136.6, 133.6. 132.1, 130.0, 128.4, 125.2, 123.7, 116.4, 115.3, 40.6; LC-HRMS (ESI^+^): [M+H]^+^ calcld for C_15_H_12_Cl_2_N_2_O_3_: 339.172; found 340.020.

***N'*-(2,6-dichlorobenzylidene)-2-(3,4-dihydroxyphenyl)acetohydrazide (22)**

Yield: 0.21 g (81%); (Dark brown powder); M.P.: 262ºC; ^1^H-NMR (600MHz, DMSO-d_6,_ *δ*, ppm): 11.12 (s, 1H, -NH), 9.48 (s, 2H, (-OH)_2,_ H-3/4), 8.35 (s, 1H, =CH), 7.42 (s, 3H, H-3’/4’/5’), 6.89 (s, 1H, H-2), 6.64-6.63 (d, 1H, *J* _5,6_ = 6.6 Hz, H-5), 6.54-6.53 (d, 1H, *J* _6,5_ = 6.6 Hz, H-6), 3.81 (s, 2H, -CH_2_, H-Ph); ^13^C NMR (150 MHz, DMSO‑d_6_): *δ* 171.4, 148.3, 148.3, 138.7, 133.6, 133.6, 132.1, 130.0, 129.4, 128.2, 128.2, 123.7, 116.4, 115.3, 40.6; LC-HRMS (ESI^+^): [M+H]^+^ calcld for C_15_H_12_C_l2_N_2_O_3_: 340.020; found 287.099.

**2-(3,4-dihydroxyphenyl)-*N*'-(4-nitrobenzylidene)acetohydrazide (23)**

Yield: 0.22 g (77%); (Deep yellow powder); M.P.: 201ºC; ^1^H-NMR (600 MHz, DMSO-d_6,_ *δ*, ppm): 11.24 (s, 1H, -NH), 9.53 (s, 2H, (-OH)_2_), 8.58 (s, 1H, =CH), 8.45-8.44 (d, 2H, *J* _3’,2’/5’,6’_ = 7.2 H-3’/5’), 8.14-8.13 (d, 1H, *J* _2’,3’/6’,5’_ = 7.2 Hz, H-2’/6’), 6.87 (s, 1H, H-2), 6.74-6.73 (d, 1H, *J* _5,6_ = 6.6 Hz, H-5), 6.51-6.49 (d, 1H, *J* _6,5_ = 6.6 Hz, H-6), 3.85 (s, 2H,-CH_2_, H-Ph); 6^13^C NMR (150 MHz, DMSO‑d_6_): *δ* 171.0, 150.2, 146.4, 146.4, 145.2, 139.8, 130.1, 124.8, 124.8, 124.2, 124.2, 123.3, 118.1, 116.5, 40.6; LC-HRMS (ESI^+^): [M+H]^+^ calcld for C_15_H_13_N_3_O_5_: 315.285; found 316.089.

**2-(3,4-dihydroxyphenyl)-*N*'-(2-nitrobenzylidene)acetohydrazide (24)**

Yield: 0.28 g (87%); (Yellow powder); M.P.: 204ºC; ^1^H-NMR (600 MHz, DMSO-d_6,_ *δ*, ppm): 11.28 (s, 1H, -NH), 9.64 (s, 2H, (-OH)_2_), 8.73 (s, 1H, =CH), 8.45-8.44 (d, 1H, *J* _6’,5’_ = 7.2, H-6’), 8.34-8.33 (d, 1H, *J* _3’,4’_ = 7.2 Hz, H-3’), 7.97-7.96 (t, 1H, *J* _5’(4’,6’)_ = 7.6 Hz, H-5’), 7.84-7.83 (t, 1H, *J* _4’(3’,5’)_ = 7.6 Hz, H-4’), 6.97 (s, 1H, H-2), 6.81-6.79 (d, 1H, *J* _5,6_ = 6.6 Hz, H-5), 6.61-6.59 (d, 1H, *J* _6,5_ = 6.6 Hz, H-6), 3.95 (s, 2H,-CH_2_, H-Ph); ^13^C NMR (150 MHz, DMSO‑d_6_): *δ* 171.3, 150.4, 148.4, 148.4, 144.2, 139.5, 130.2, 126.6, 126.6, 125.7, 125.7, 123.3, 116.1, 113.5 40.6; LC-HRMS (ESI^+^): [M+H]^+^ calcld for C_15_H_13_N_3_O_5_: 315.285; found 316.089.

**2-(3,4-dihydroxyphenyl)-*N*'-(3-nitrobenzylidene)acetohydrazide (25)**

Yield: 0.24 g (82%); (Yellow powder); M.P.: 206ºC; ^1^H-NMR (600 MHz, DMSO-d_6,_ *δ*, ppm): 11.11 (s, 1H, -NH), 9.56 (s, 2H, (-OH)_2_), 8.53 (s, 2H,H-(=CH)/2’), 8.21-8.10 (m, 2H, H- 4’/6’), 7.84-7.83 (t, 1H, *J* _5’(4’,6’ )_ = 7.2 Hz, H-5’), 6.89 (s, 1H, H-2), 6.76-6.75 (d, 1H, *J* _5,6_ = 7.2 Hz, H-5), 6.62-6.61 (d, 1H, *J* _6,5_ = 7.2 Hz, H-6), 3.95 (s, 2H,-CH_2_, H-Ph); ^13^C NMR (150 MHz, DMSO‑d_6_): *δ* 171.3, 147.4, 146.2, 146.2, 142.5, 134.2, 132.6, 131.7, 129.3, 126.1, 123.7, 121.6, 116.3, 115.4, 40.6; LC-HRMS (ESI^+^): [M+H]^+^ calcld for C_15_H_13_N_3_O_5_: 315.285; found 316.089.

**2-(3,4-dihydroxyphenyl)-*N*'-(4-formylbenzylidene)acetohydrazide (26)**

Yield: 0.28 g (89%); (Pale yellow powder). M.P.: 204ºC; ^1^H-NMR (600 MHz, DMSO-d_6,_ *δ*, ppm): 11.10 (s, 1H, -NH), 9.98 (s, 1H, =CH, H-4’), 9.48 (s, 2H, (-OH)_2_), 8.45 (s, 1H, =CH), 8.00-7.98 (d, 2H, *J* _3’,2’/5’, 6_ = 8.4 Hz, H-3’/5’), 7.84-7.83 (d, 2H, *J* _2’,3’/6’,5’_ = 7.2 Hz, H-2’/6’), 6.89 (s, 1H, H-2), 6.74-6.73 (d, 1H, *J* _6’,5’_ = 7.2 Hz, H-6’), 6.64-6.63 (d, 1H, *J* _5’,6’_ = 6.6 Hz, H-5’), 3.81 (s, 2H, -CH_2_, H-Ph); 6^13^C NMR (150 MHz, DMSO‑d_6_): *δ* 171.4, 170.2, 148.2, 148.2, 146.4, 139.2, 138.7, 132.6, 130.0, 130.0, 129.4, 129.4, 122.3, 116.4, 115.2, 40.6; LC-HRMS (ESI^+^): [M+H]^+^ calcld for C_16_H_14_N_2_O_4_: 298.298; found 299.099.

**2-(3,4-dihydroxyphenyl)-*N*'-(furan-2-ylmethylene)acetohydrazide (27)**

Yield: 0.23 g (71%); (Off white powder); M.P.: 156 ºC; ^1^H-NMR (600 MHz, DMSO-d_6,_ *δ*, ppm): 11.67 (s, 1H, -NH), 10.45 (s, 2H, (-OH)_2,_ H-3/4), 9.23 (s, 1H, =CH), 8.547-8.53 (d, 1H, *J* _2’,3’_= 7.2 Hz, H-2’), 7.56-7.54 (d, 1H, *J* _4’,3’_= 7.2 Hz, H-4’), 7.28 (s, 1H, H-2) 7.18-6.97(m, 3H, H-3’/5/6), 3.92 (s, 2H,-CH_2_, H-Ph); ^13^C NMR (150 MHz, DMSO‑d_6_): *δ* 171.2, 160.2, 149.9, 148.2, 148.2, 140.5, 137.3, 124.4, 118.5, 116.3, 115.8, 112.4, 40.6; LC-HRMS (ESI^+^): [M+H]^+^ calcld for C_13_H_12_N_2_O_4_: 260.249; found 261.083;

**2-(3,4-dihydroxyphenyl)-*N*'-(4-(methylthio)benzylidene)acetohydrazide (28)**

Yield: 0.22 g (81%); (Off white powder); M.P.: 198ºC; ^1^H-NMR (600 MHz, DMSO-d_6,_ *δ*, ppm): 11.10 (s, 1H, -NH), 9.47 (s, 2H, (-OH)_2_), 8.45 (s, 1H, =CH), 7.84-7.83 (d, 2H, *J* _2’,4’/6’,5’_ = 7.2 Hz, H-2’/6’), 7.24-7.23 (d, 2H, *J* _4’,2’/5’ ,6’_ = 7.2 Hz, H-4’/5’), 6.87 (s, 1H, H-2), 6.68-6.67 (d, 1H, *J* _5,6_ = 7.2 Hz, H-5), 6.54-6.53 (d, 1H, *J* _6,5_ = 7.2 Hz, H-6), 3.86 (s, 2H, -CH_2_, H-Ph), 2.31 (s, 3H, -SCH_3_, H-4’); ^13^C NMR (150 MHz, DMSO‑d_6_): *δ* 171.4, 146.2, 146.2, 144.5, 142.7 132.6, 130.4, 129.3, 129.3, 127.7, 127.7, 124.2, 116.2, 115.6, 40.6, 15.4; LC-HRMS (ESI^+^): [M+H]^+^ calcld for C_16_H_16_N_2_O_3_S: 316.375; found 317.092.


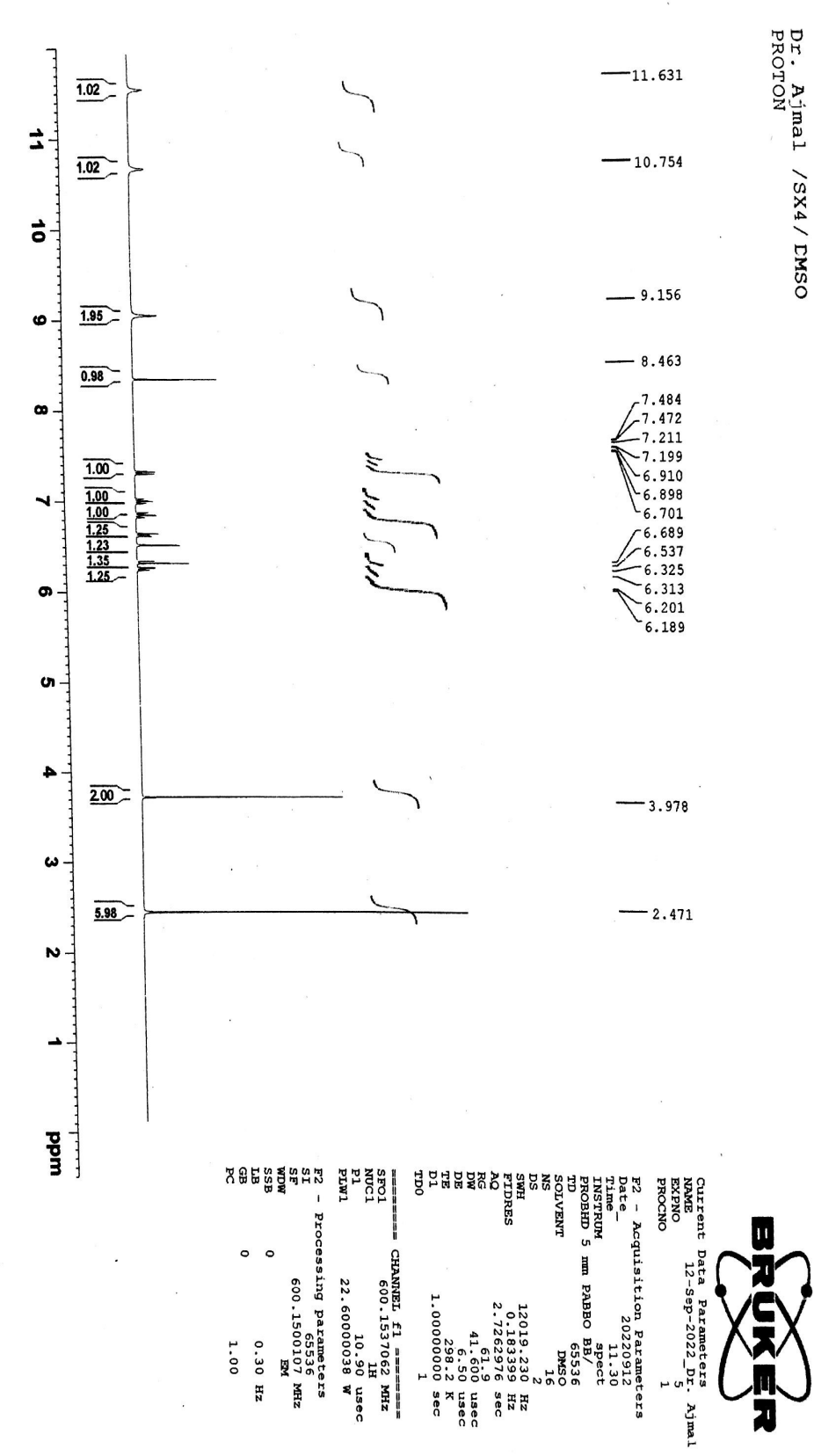


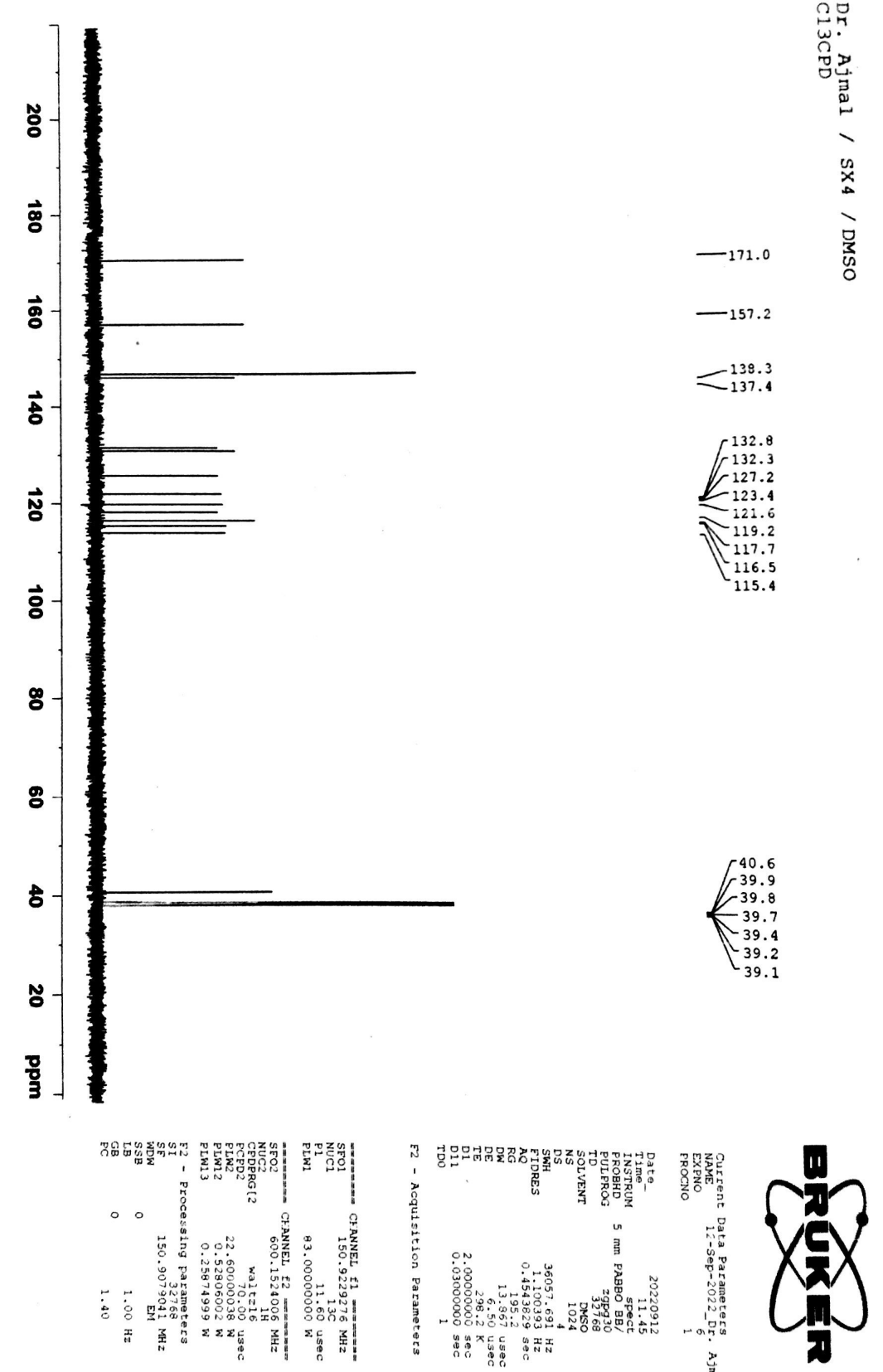


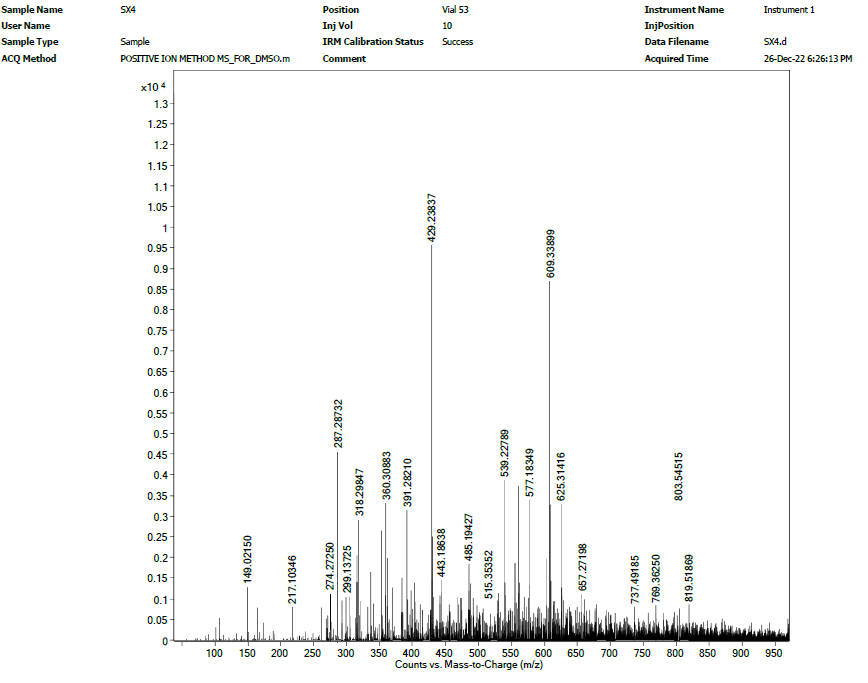


**Fig S1:** ^1^H-, ^13^C-NMR and HR-ESI-MS spectra of compound **1**


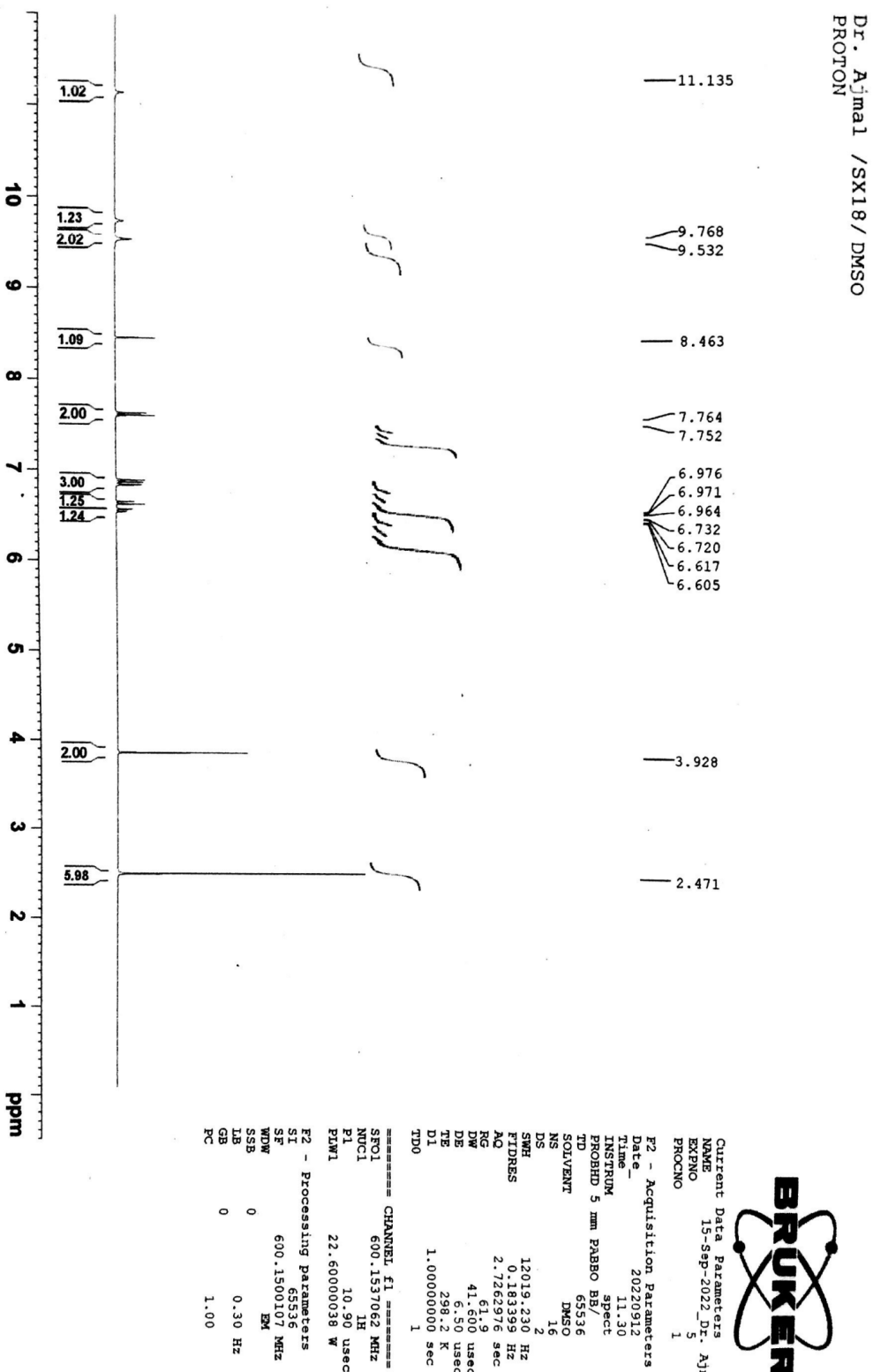


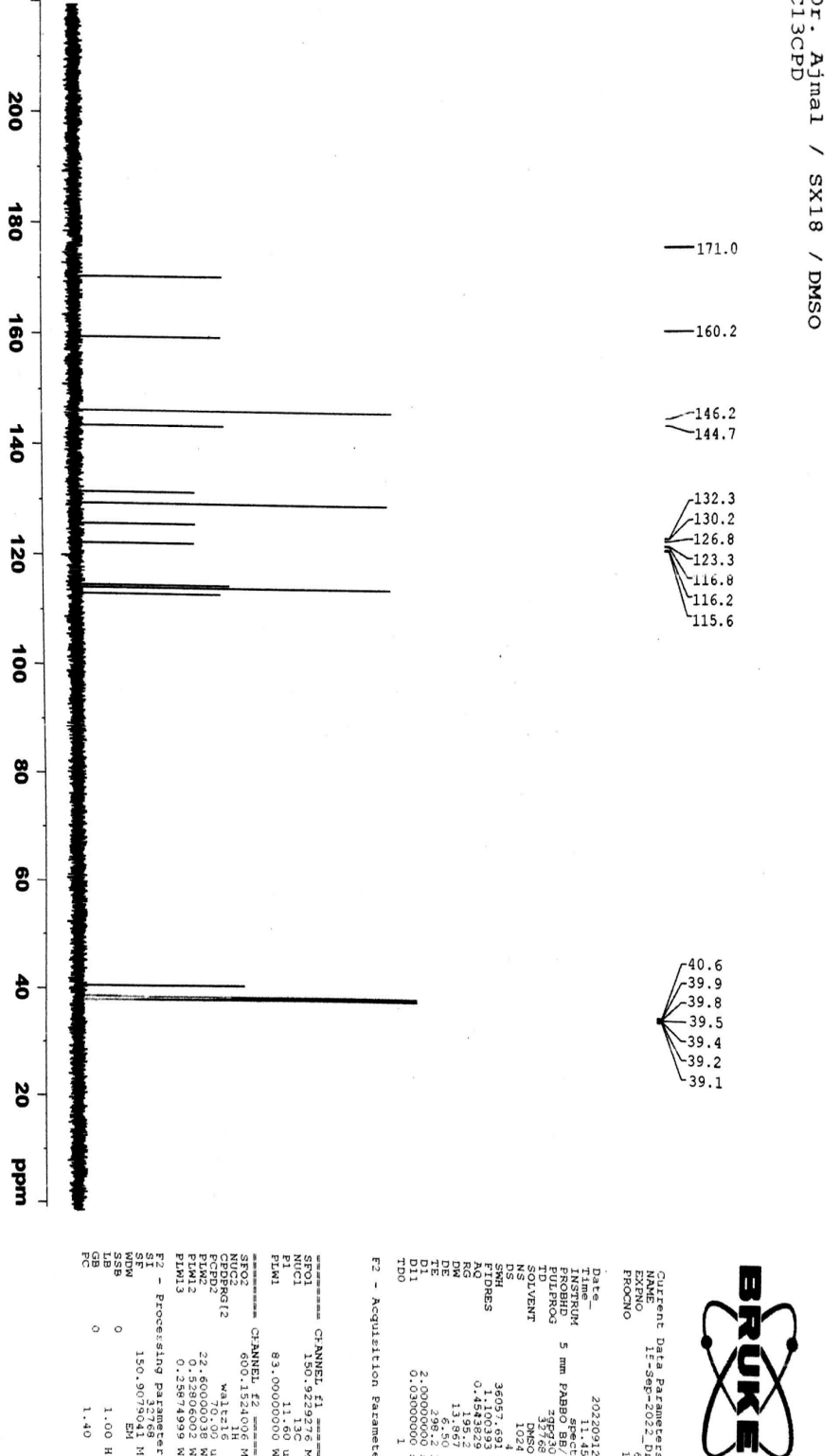


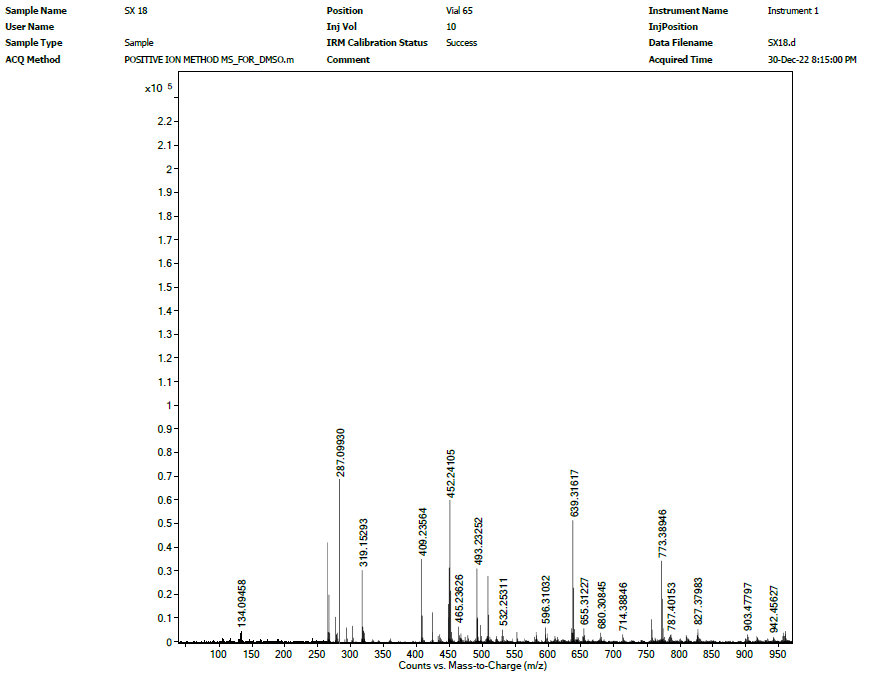


**Fig S2:** ^1^H-, ^13^C-NMR and HR-ESI-MS spectra of compound **2**


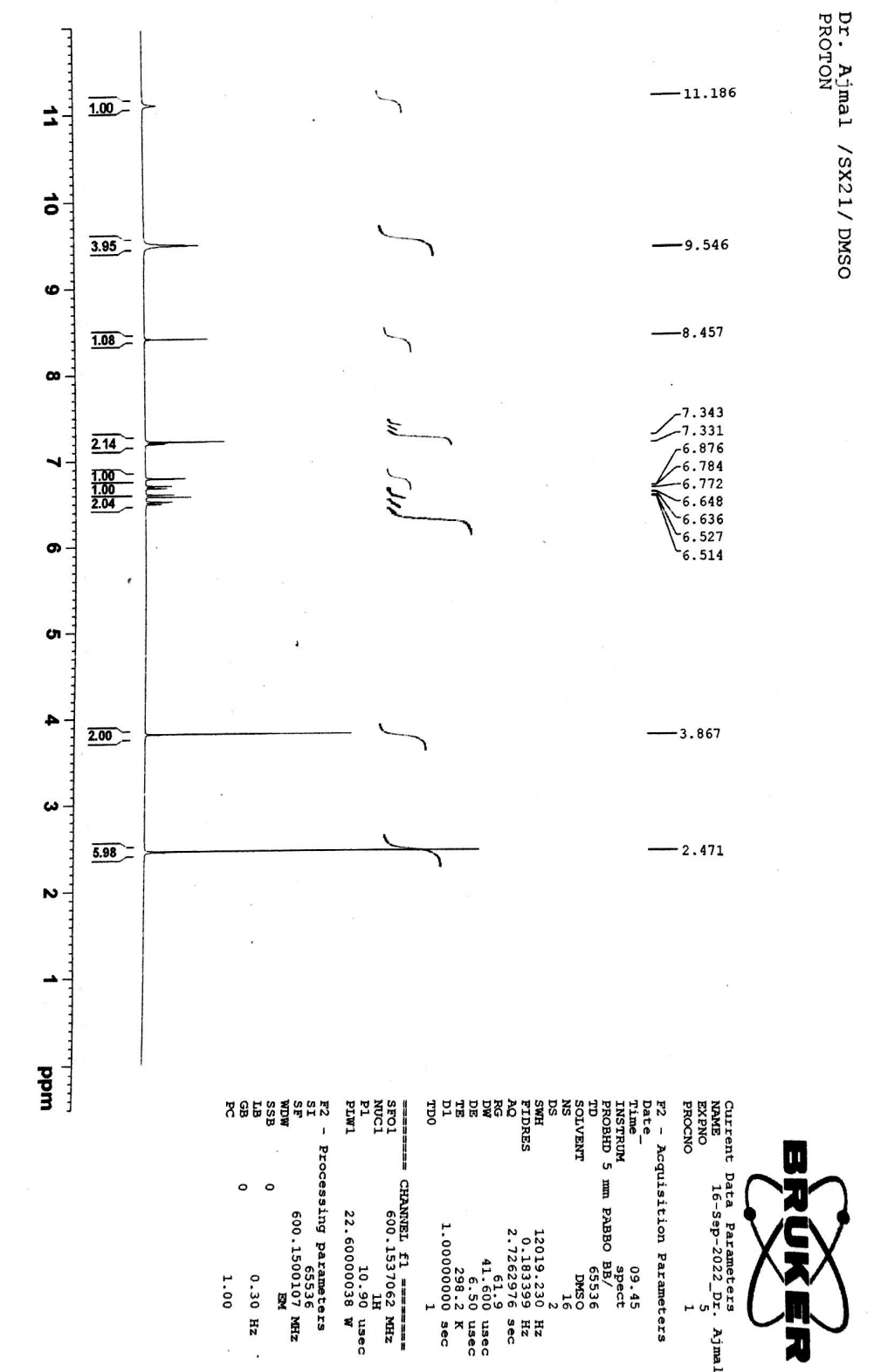


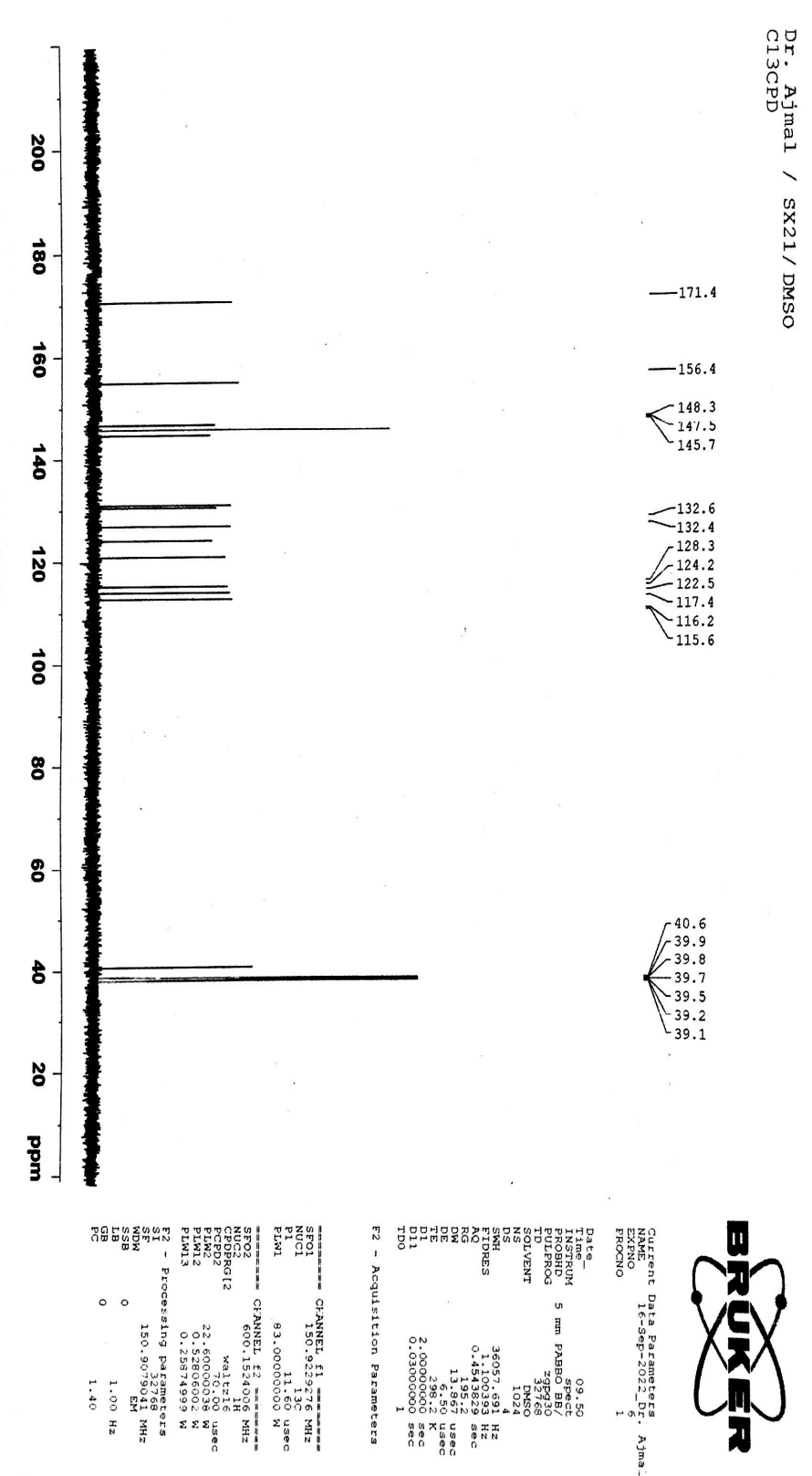


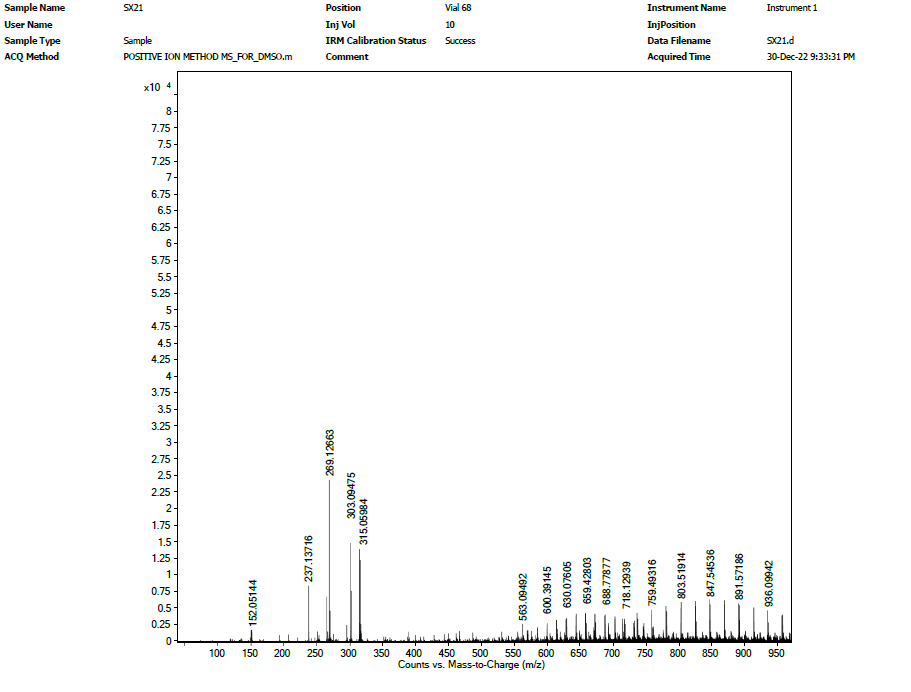


**Fig S3:** ^1^H-, ^13^C-NMR and HR-ESI-MS spectra of compound **3**


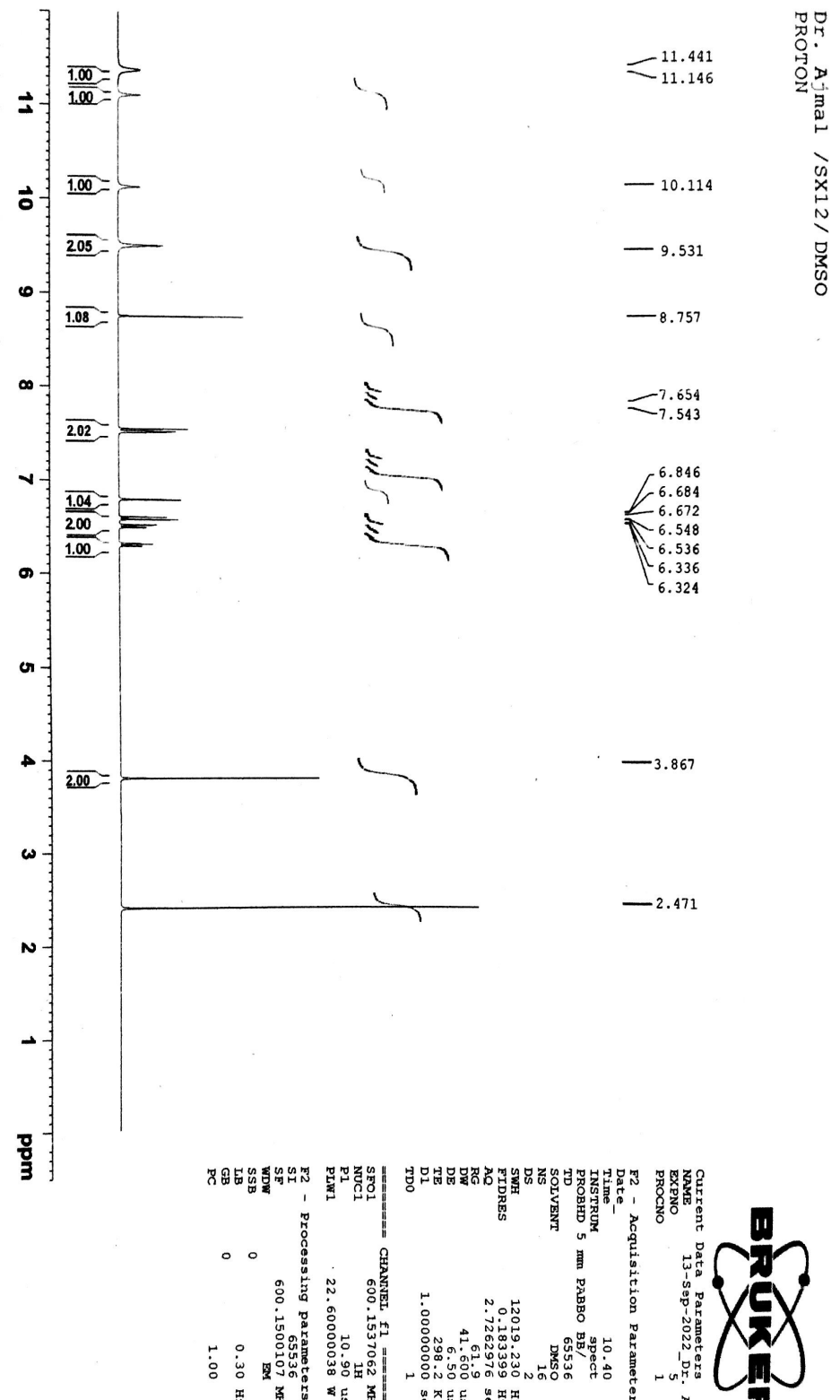

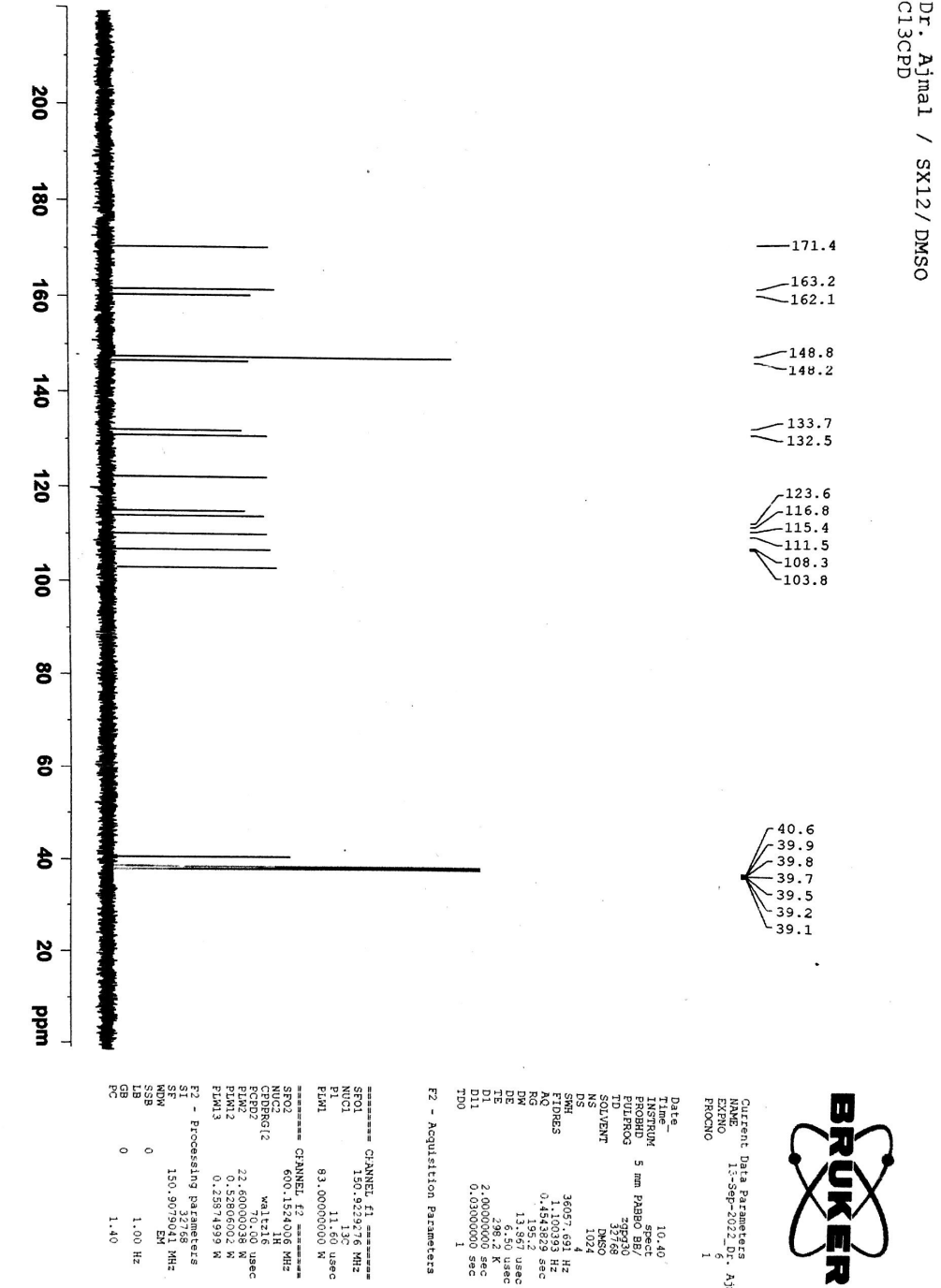


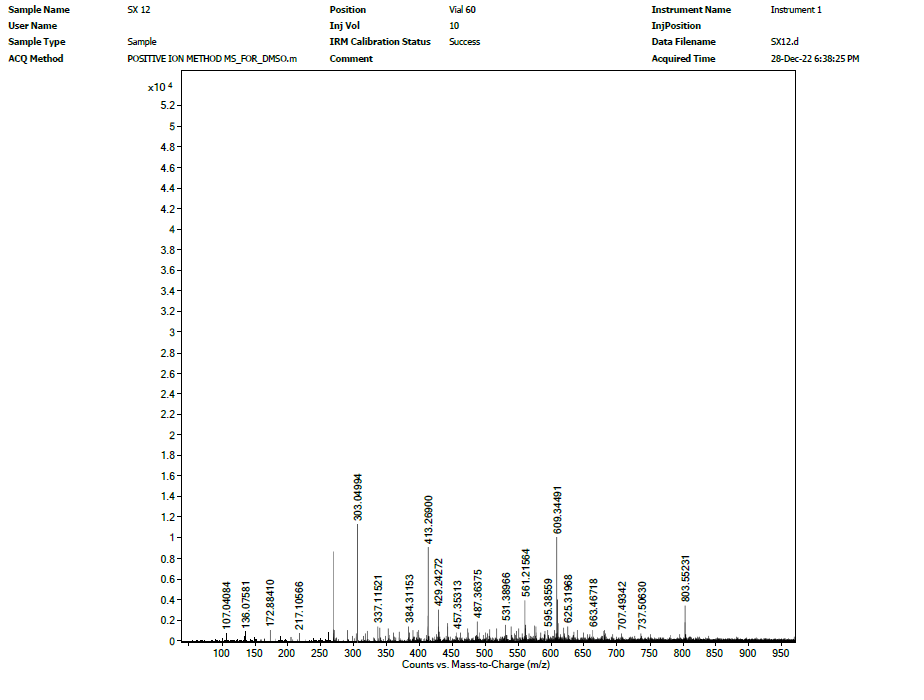


**Fig S4:** ^1^H-, ^13^C-NMR and HR-ESI-MS spectra of compound **4**


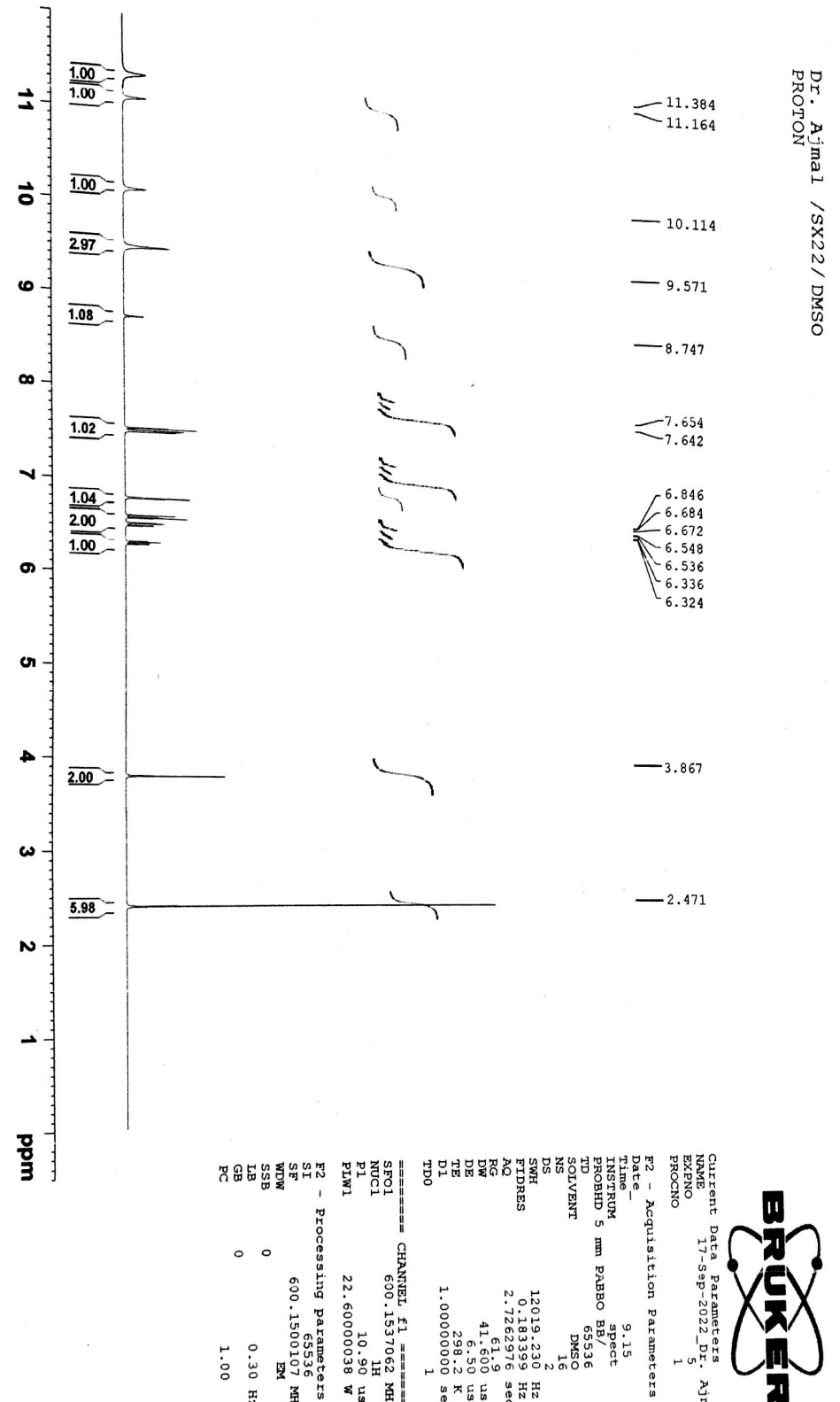


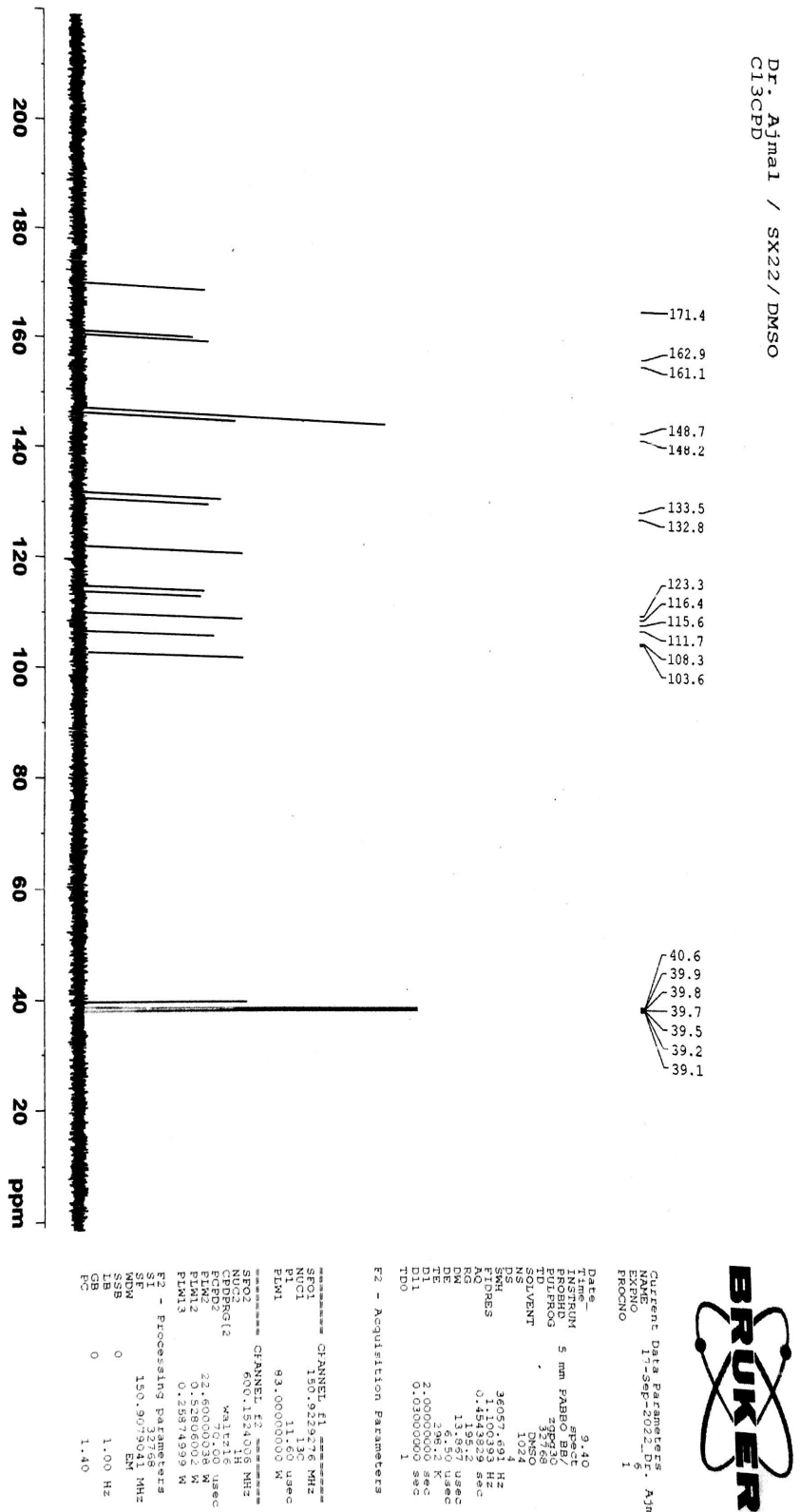


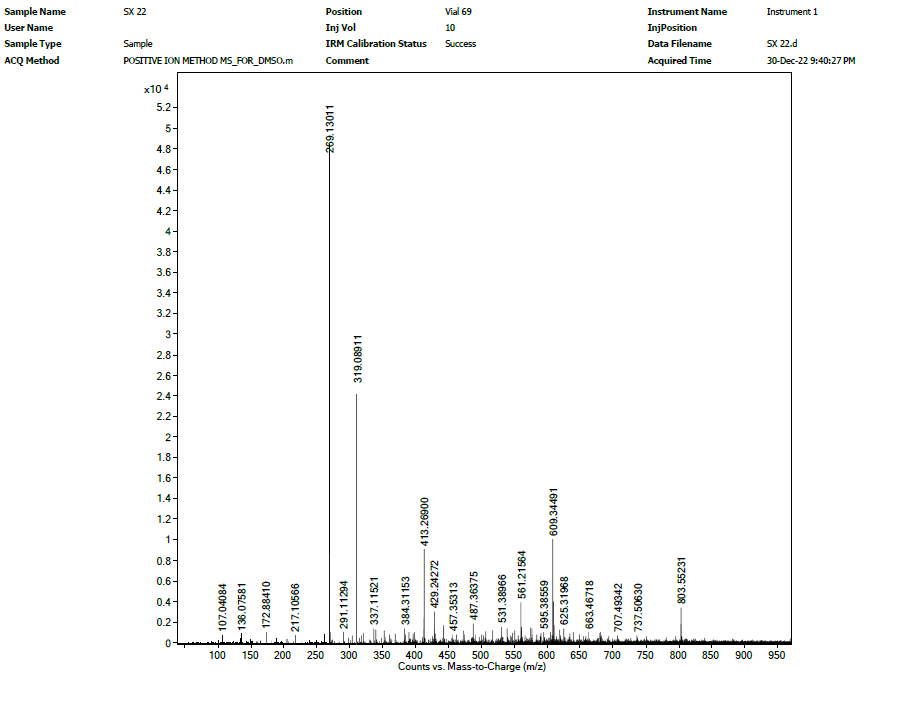


**Fig S5:** ^1^H-, ^13^C-NMR and HR-ESI-MS spectra of compound **5**


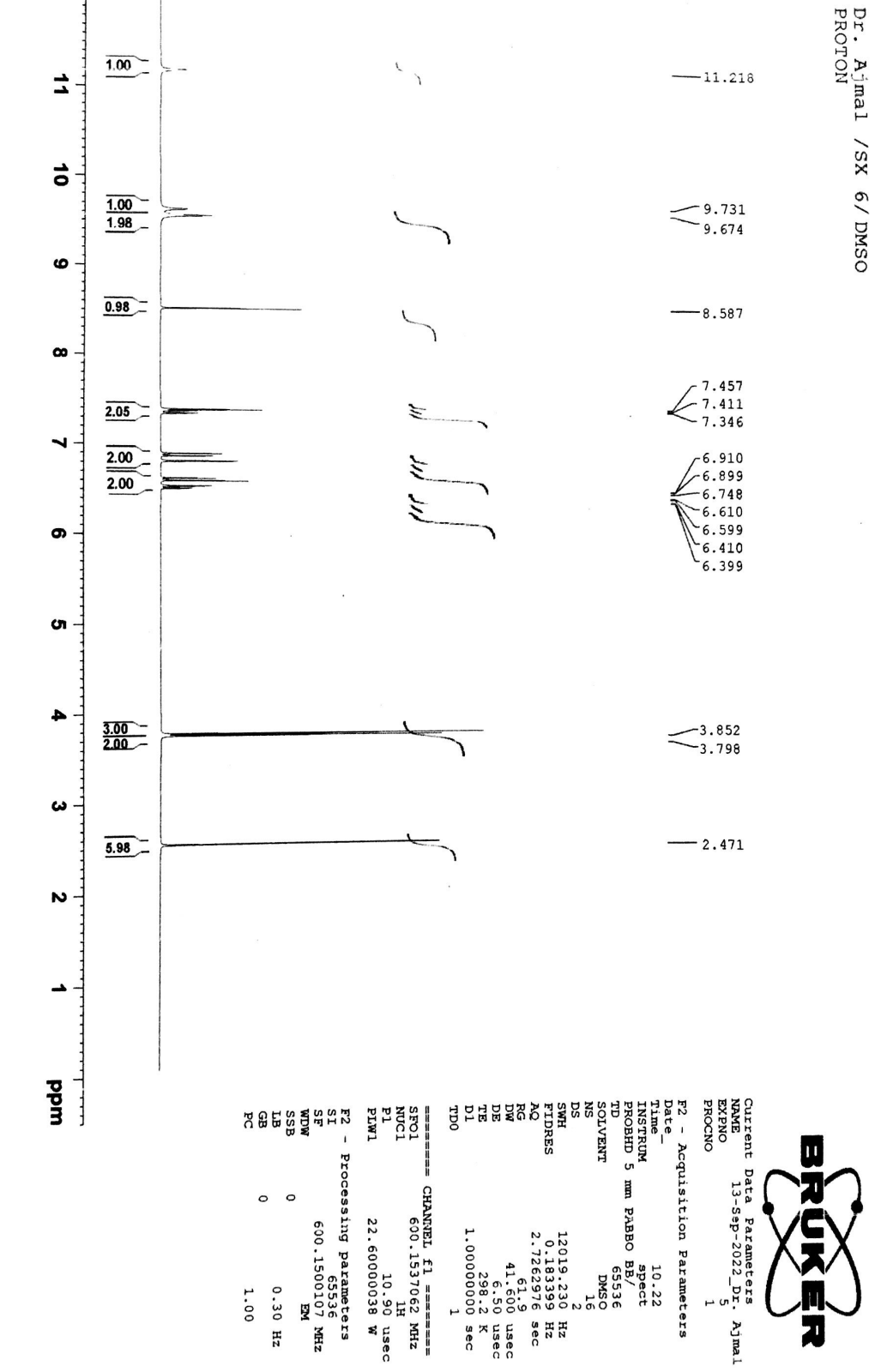


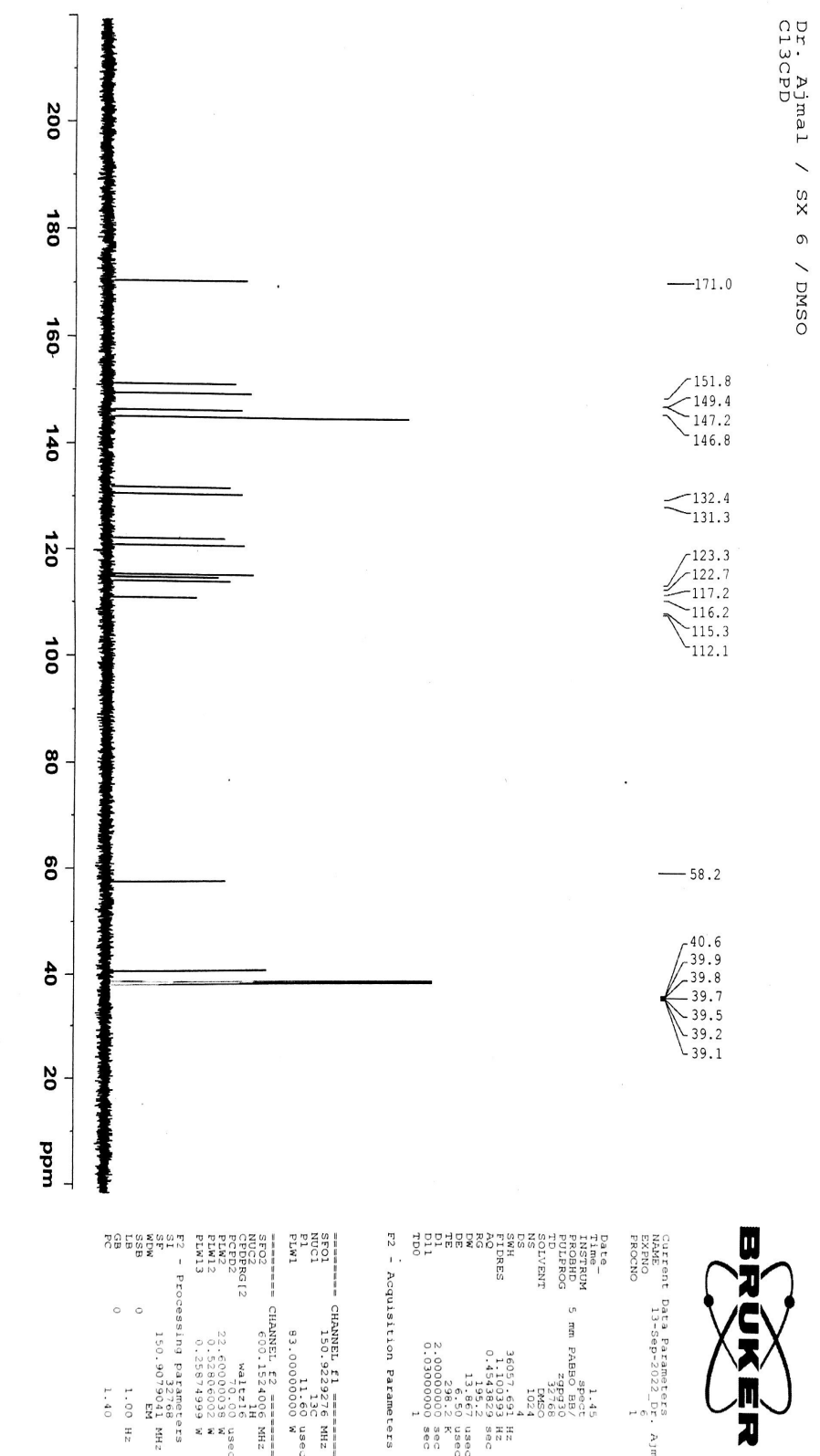


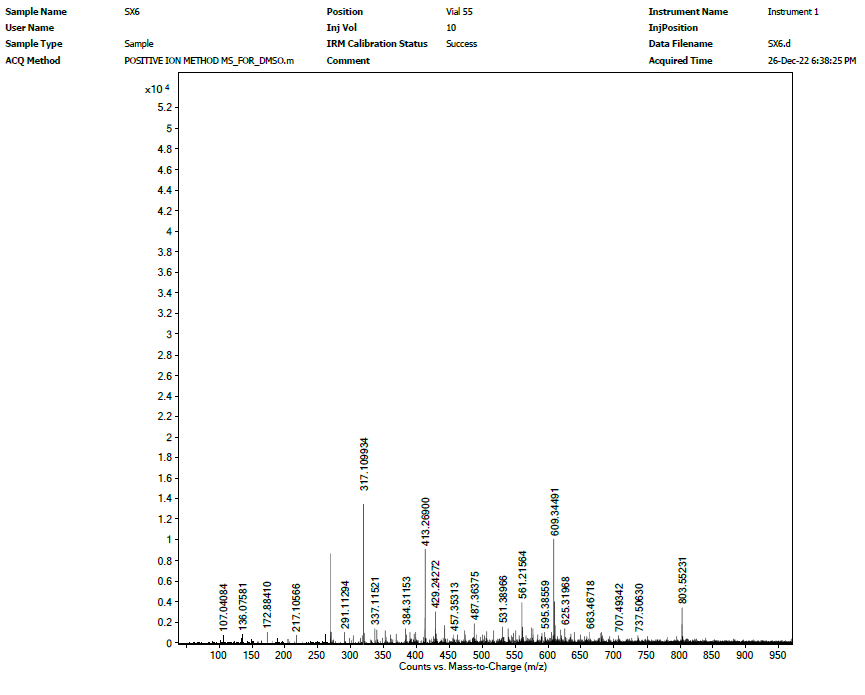
 **Fig S6:** ^1^H-, ^13^C-NMR and HR-ESI-MS spectra of compound **6**


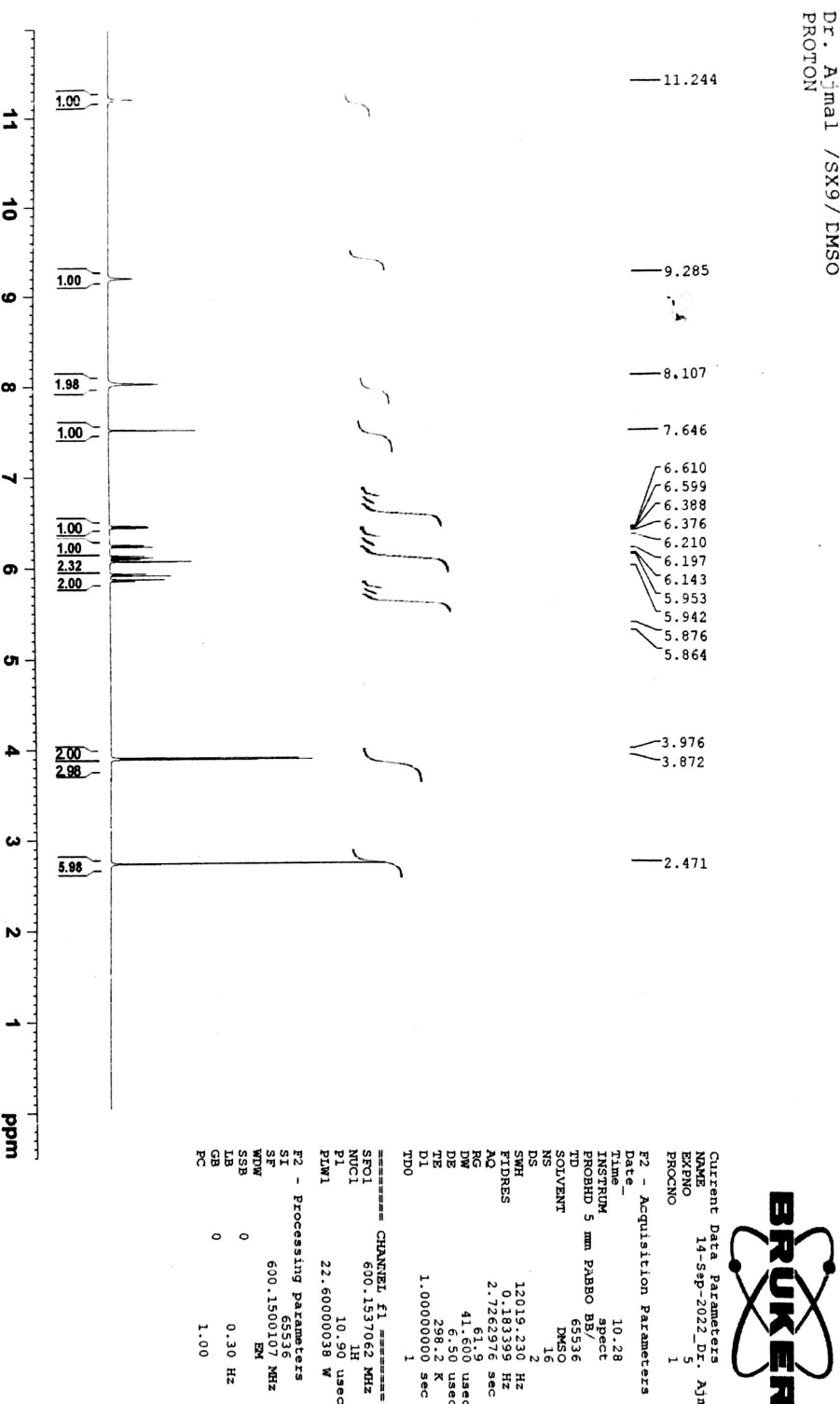


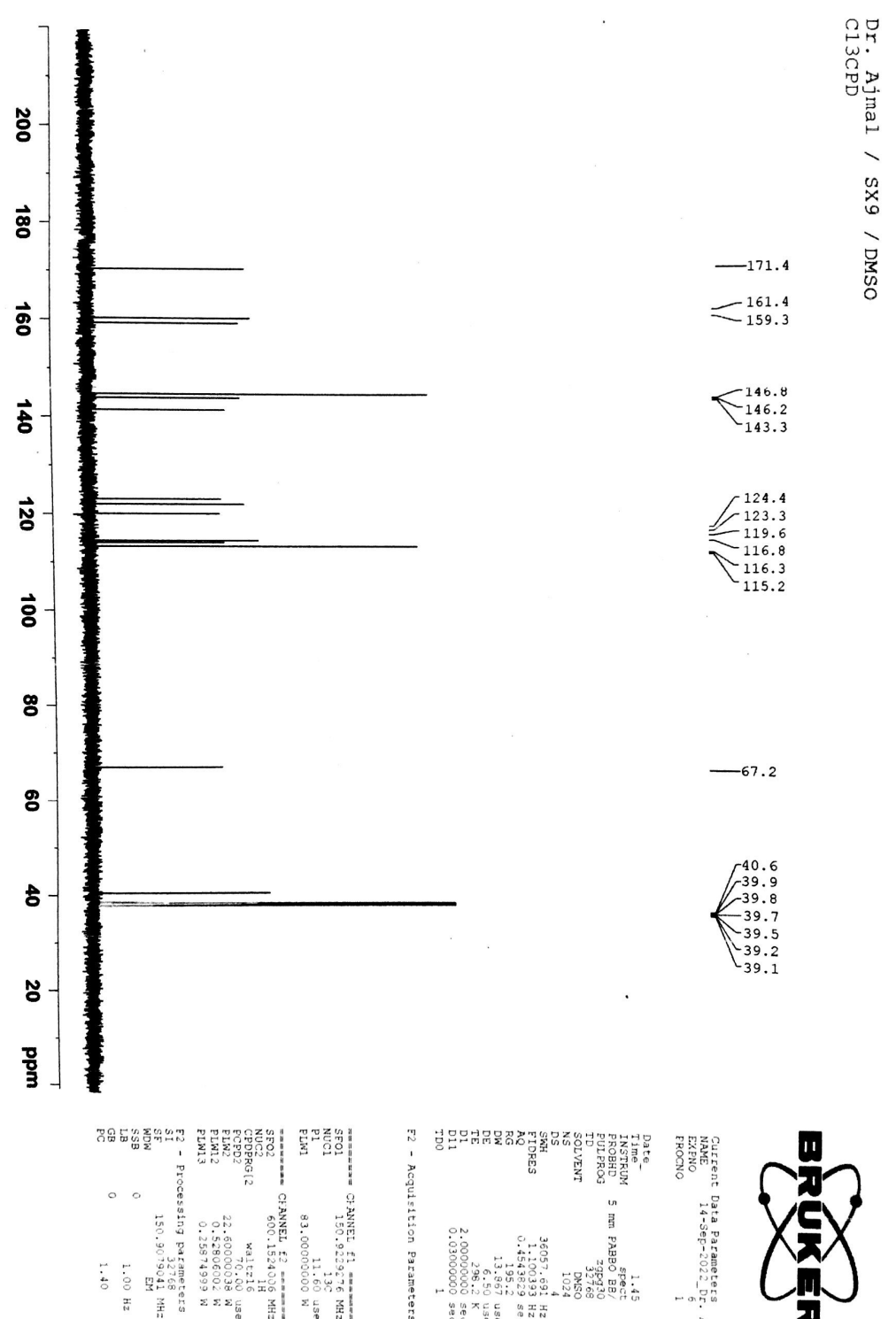


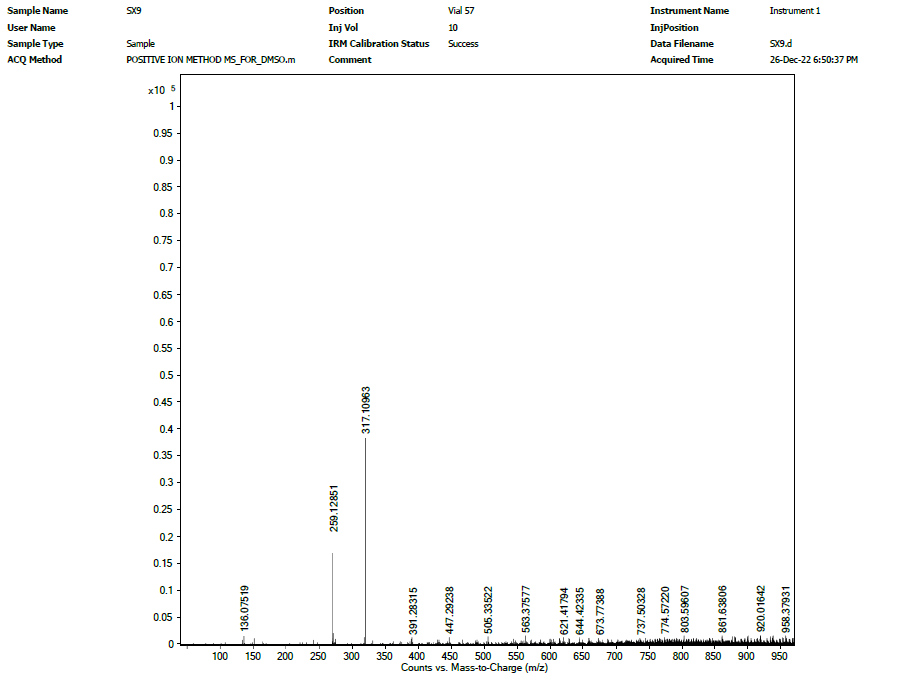


**Fig S7:** ^1^H-, ^13^C-NMR and HR-ESI-MS spectra of compound **7**


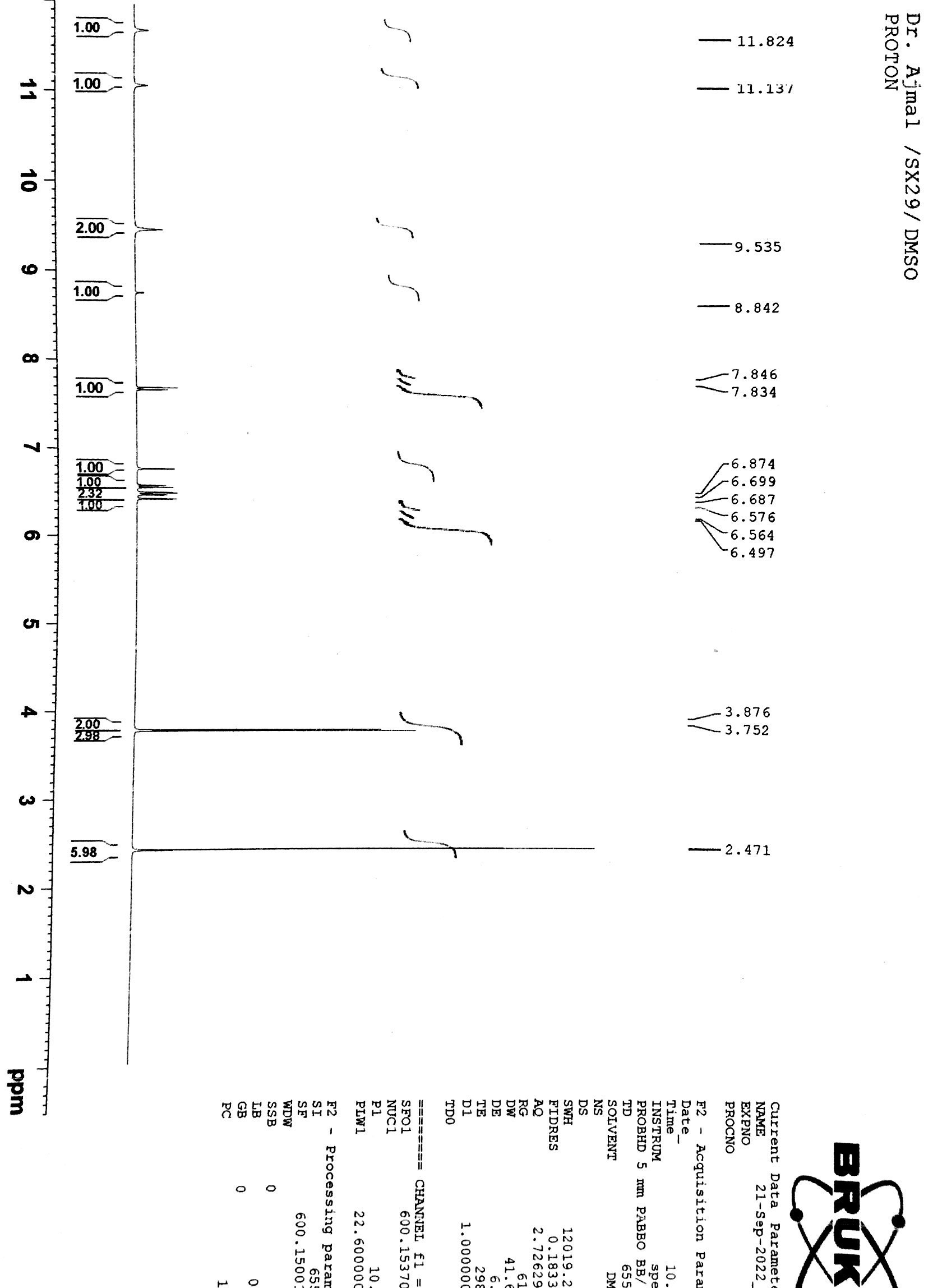


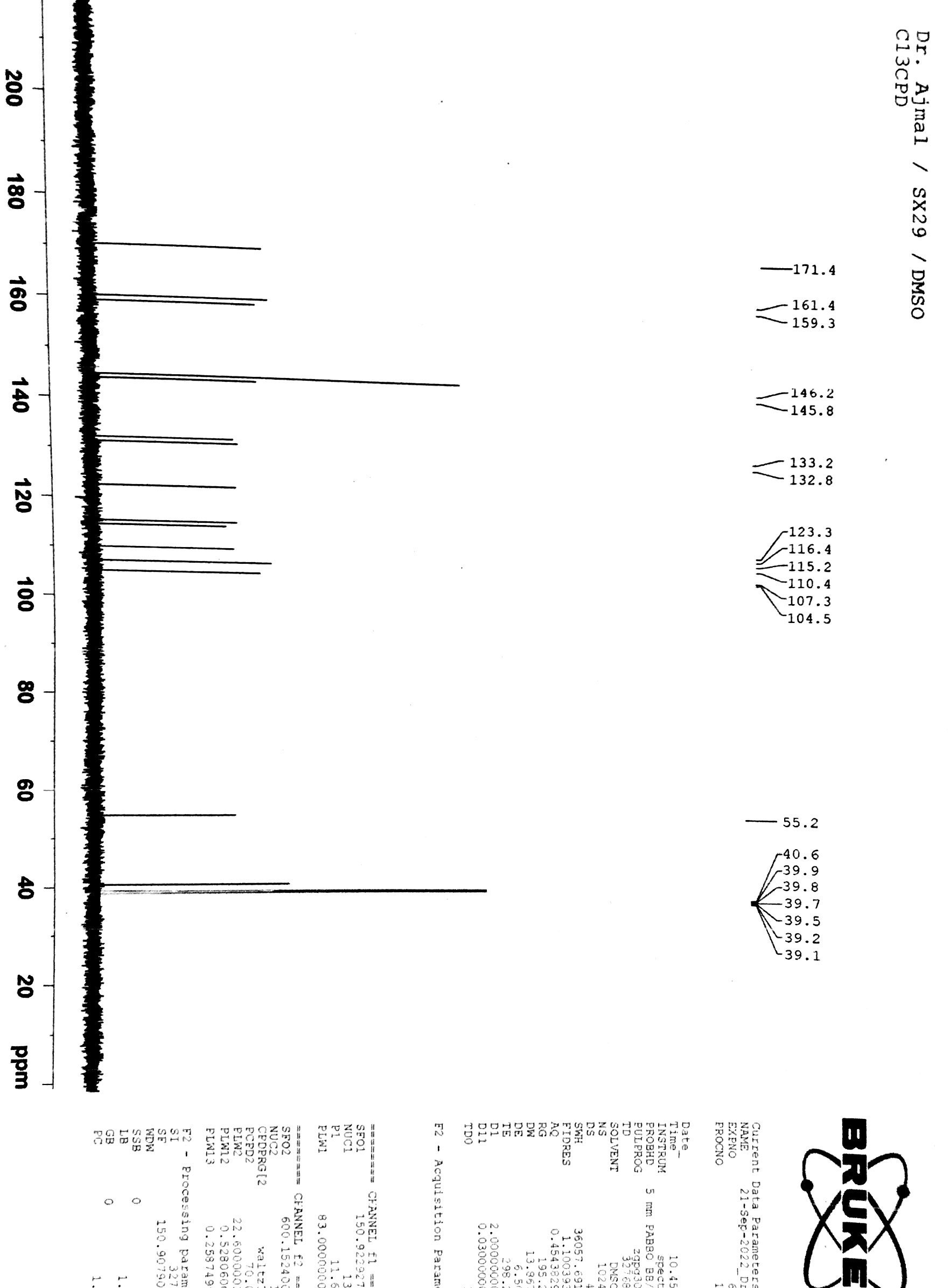


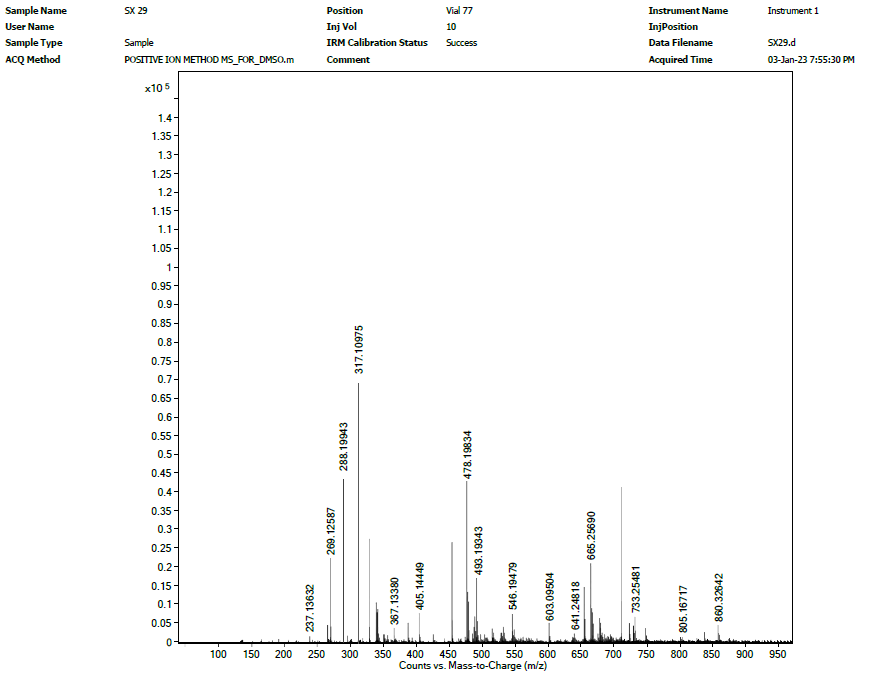


**Fig S8:** ^1^H-, ^13^C-NMR and HR-ESI-MS spectra of compound **8**


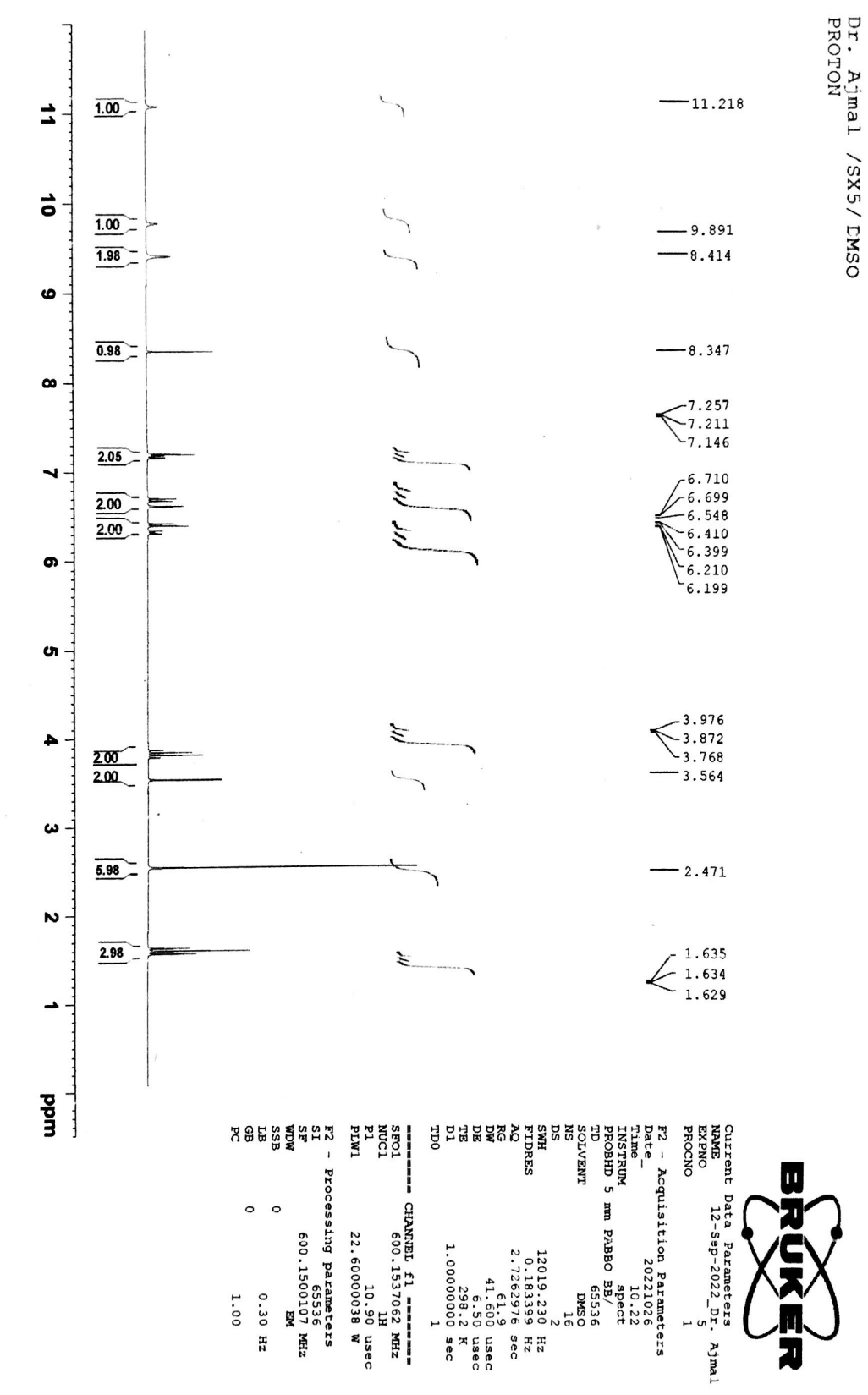


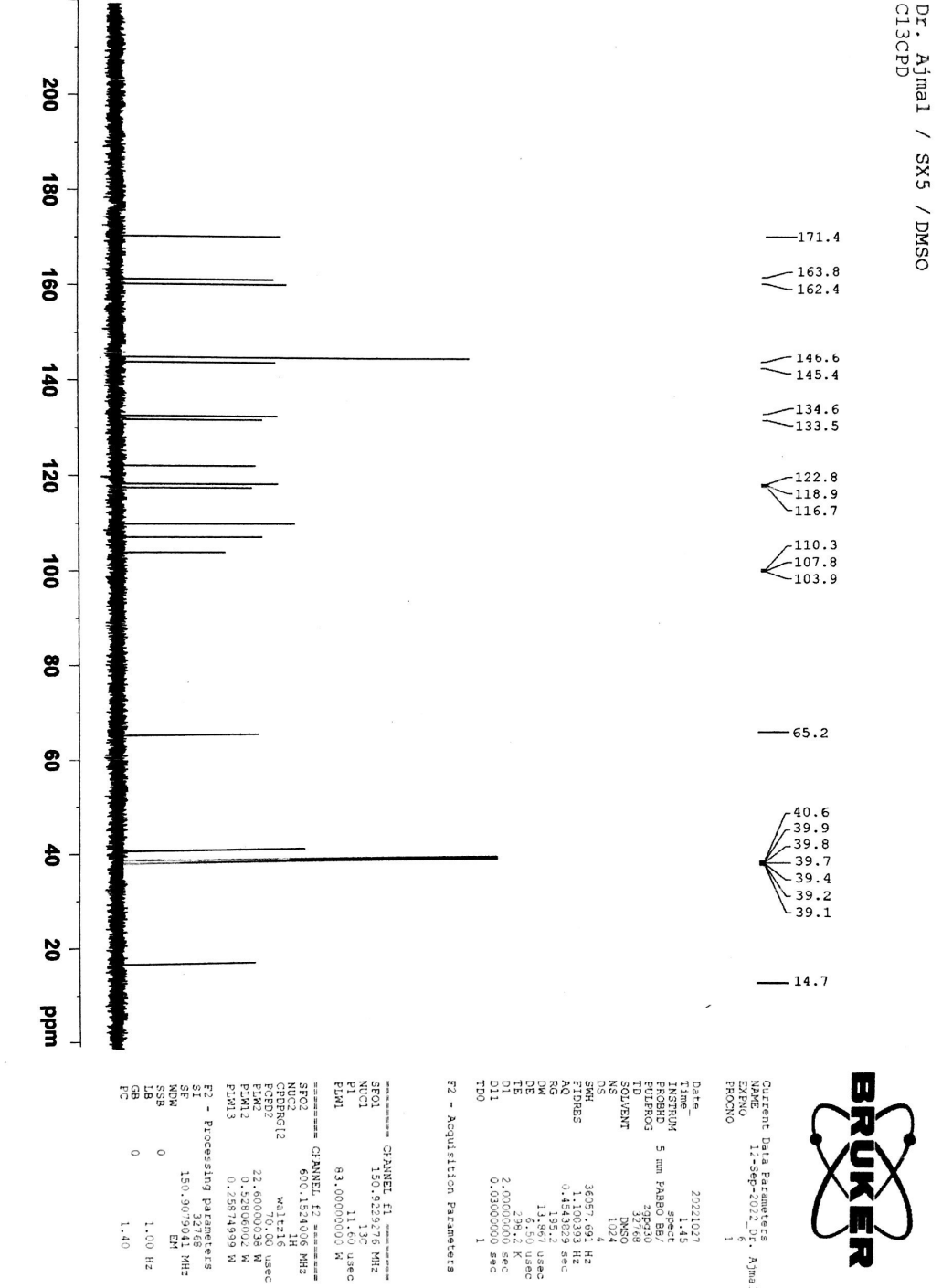


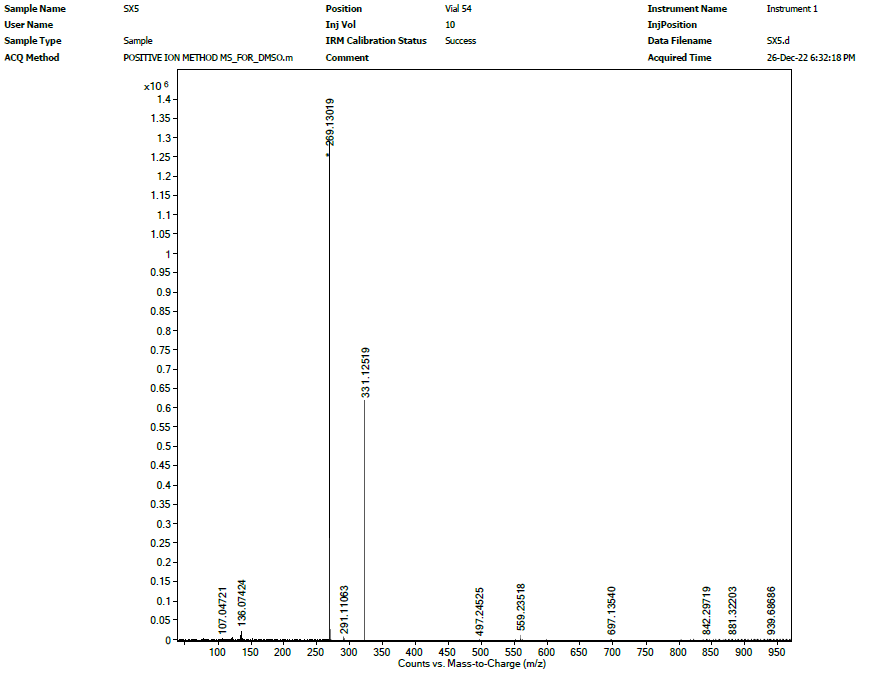


**Fig S9:** ^1^H-, ^13^C-NMR and HR-ESI-MS spectra of compound **9**

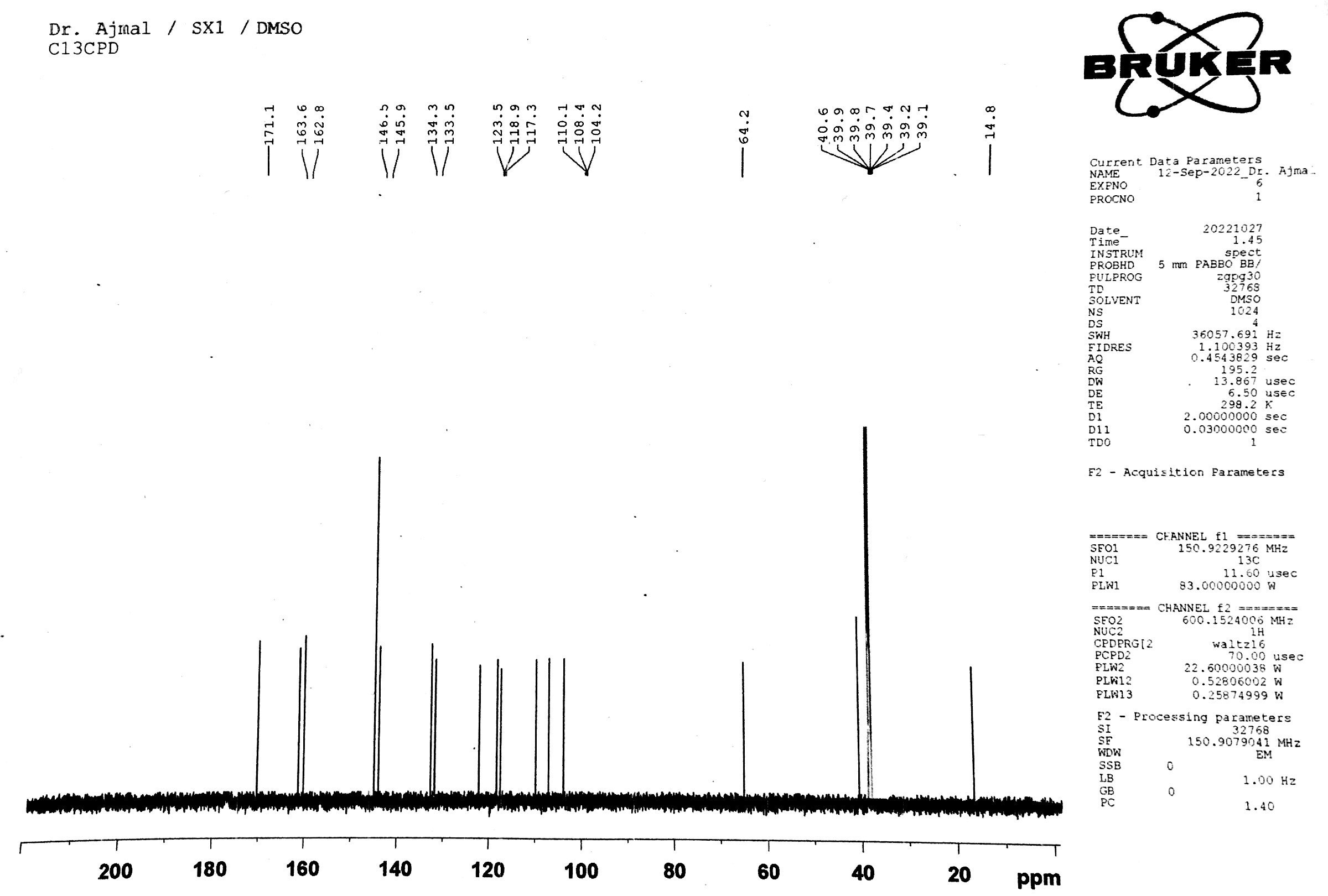


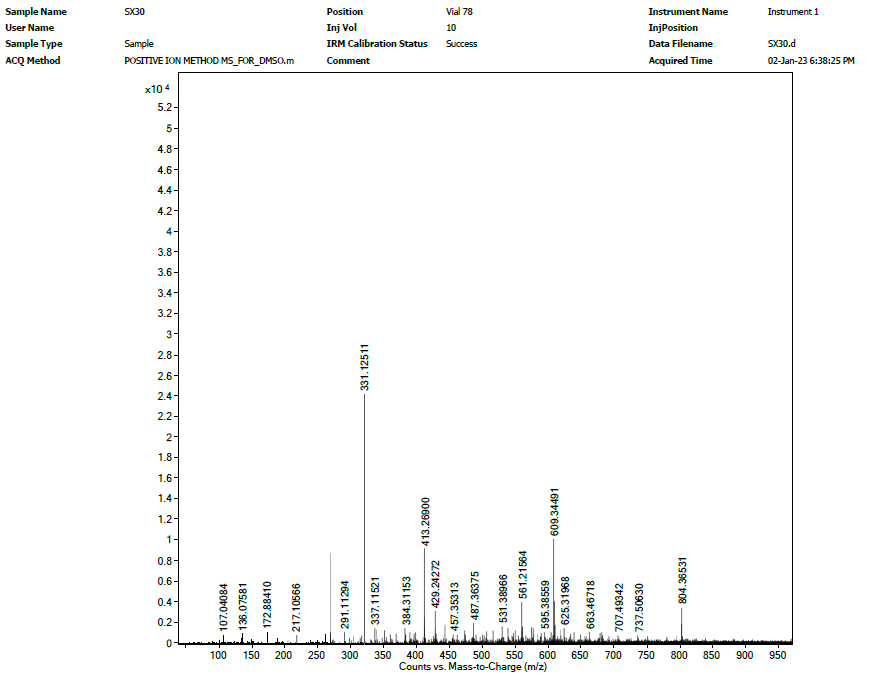


**Fig S10:** ^1^H-, ^13^C-NMR and HR-ESI-MS spectra of compound **10**


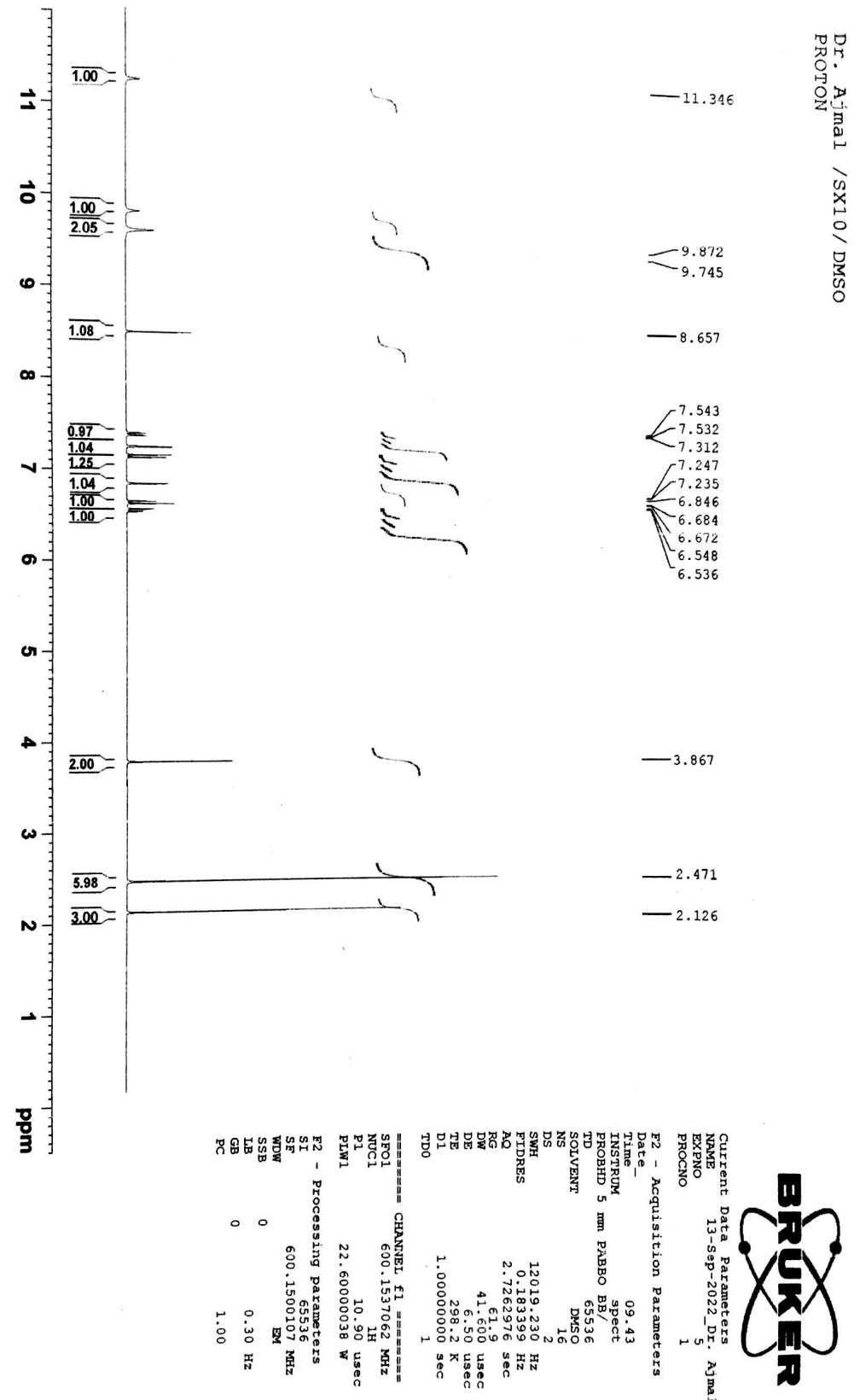


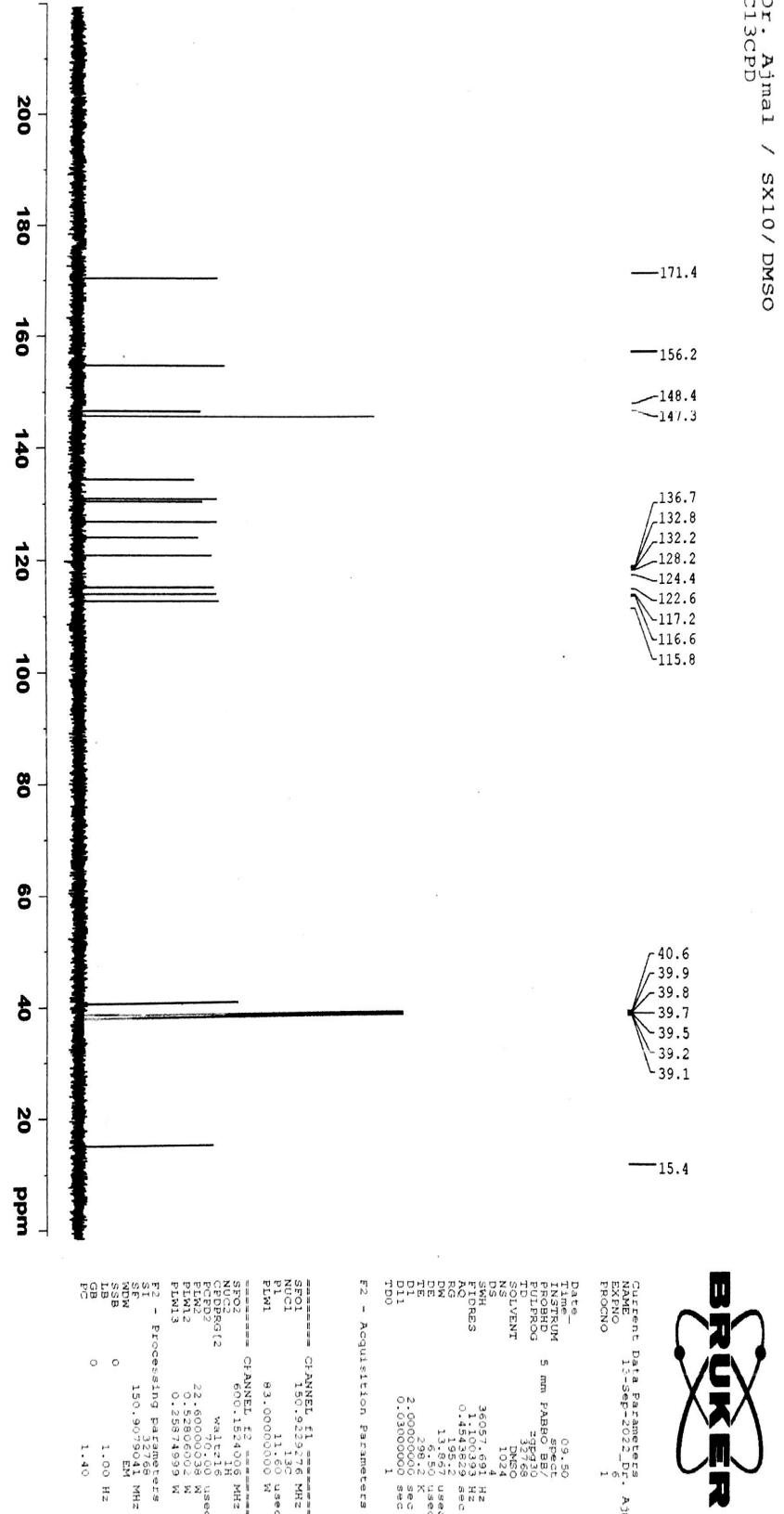


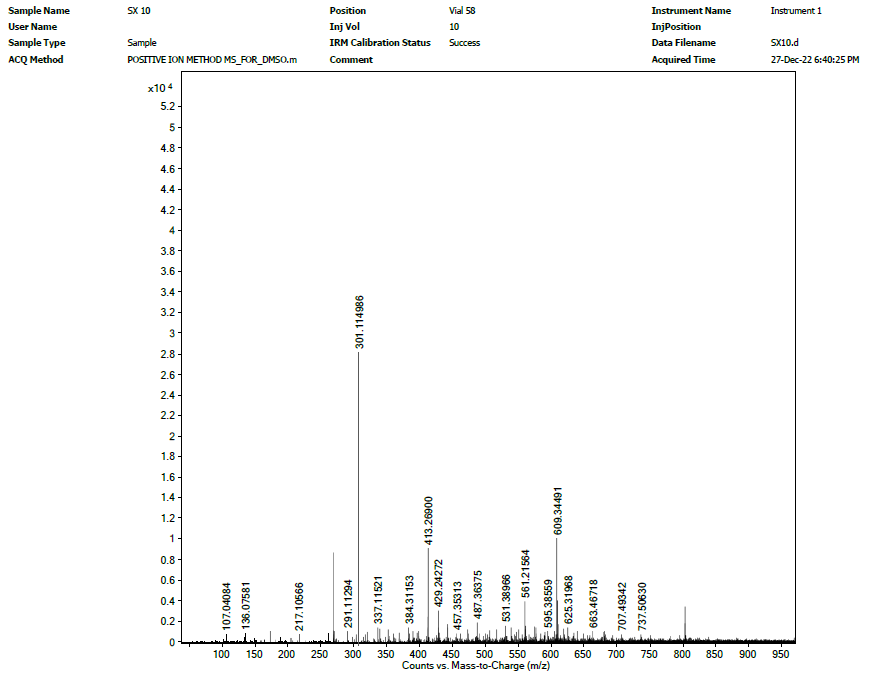


**Fig S11:** ^1^H-, ^13^C-NMR and HR-ESI-MS spectra of compound **11**


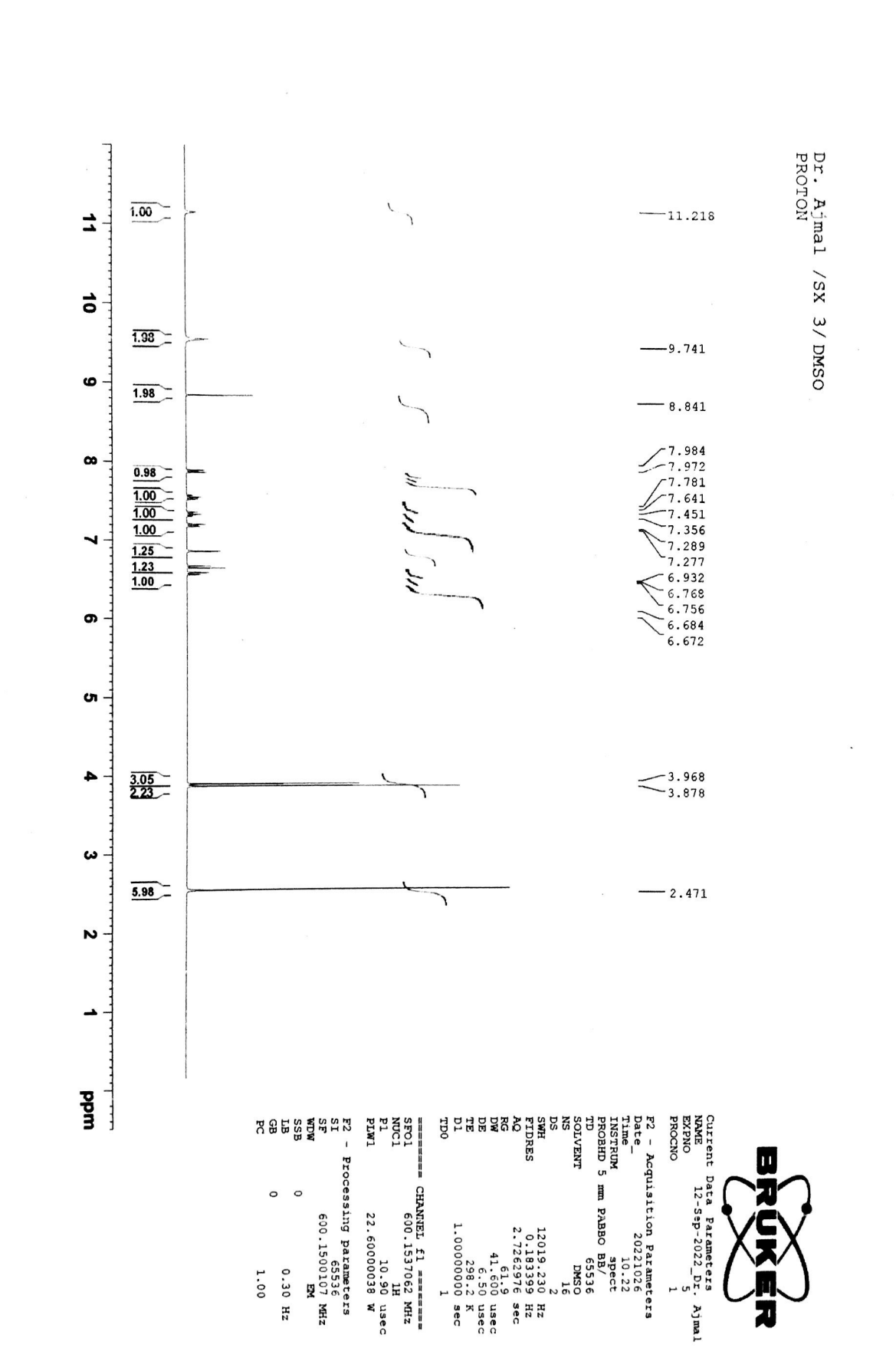


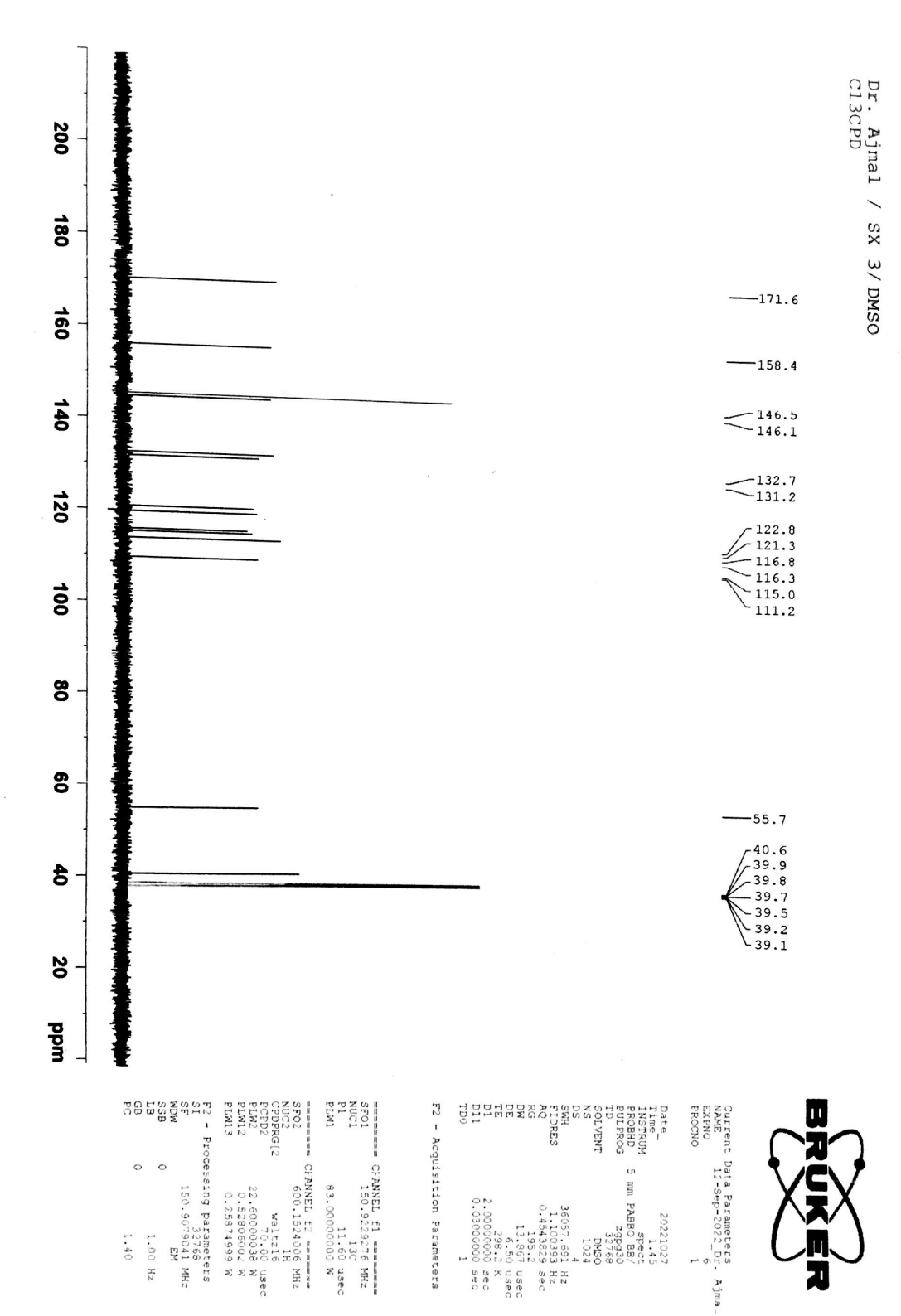


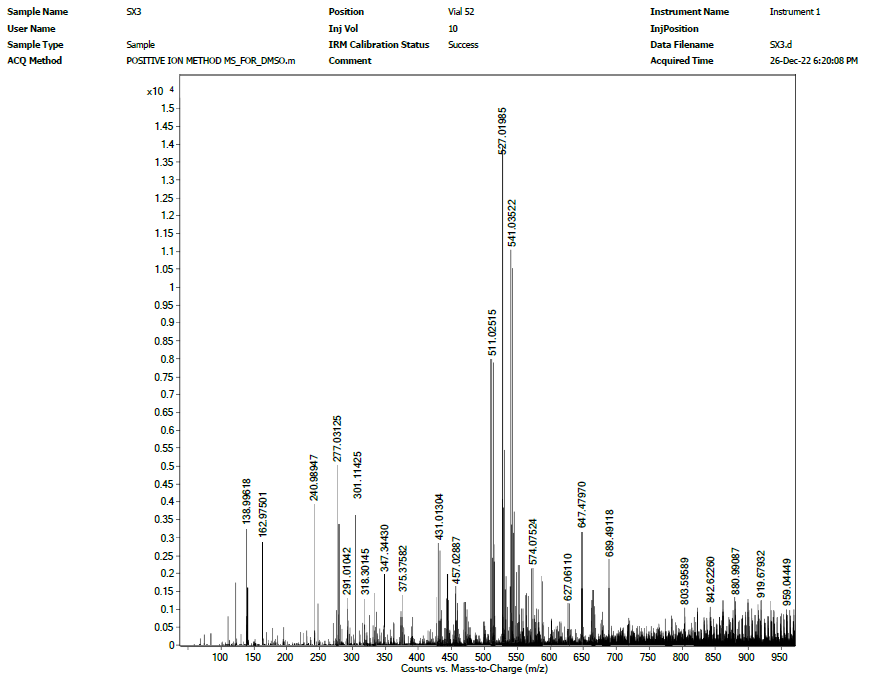


**Fig S12:** ^1^H-, ^13^C-NMR and HR-ESI-MS spectra of compound **12**


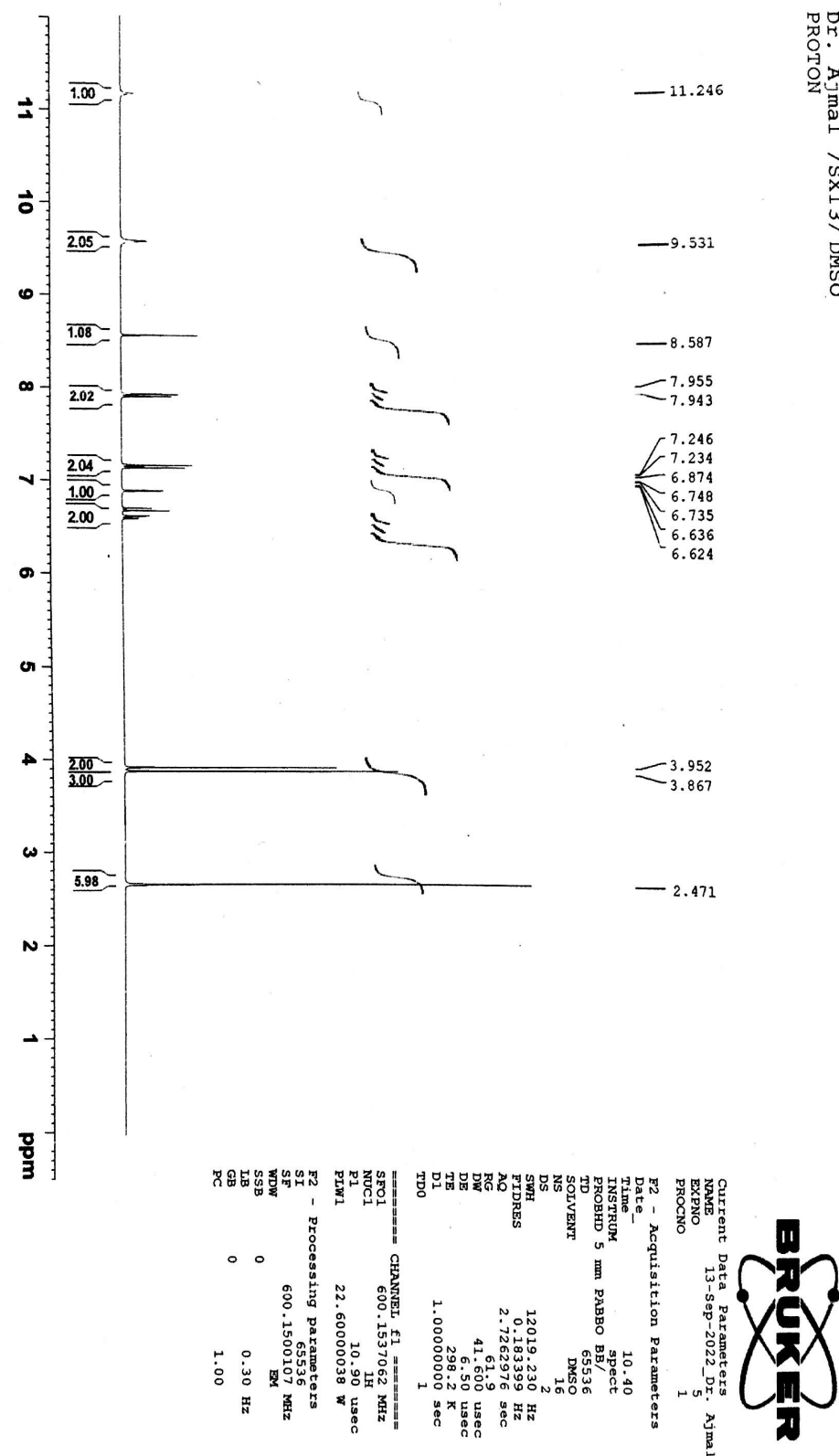


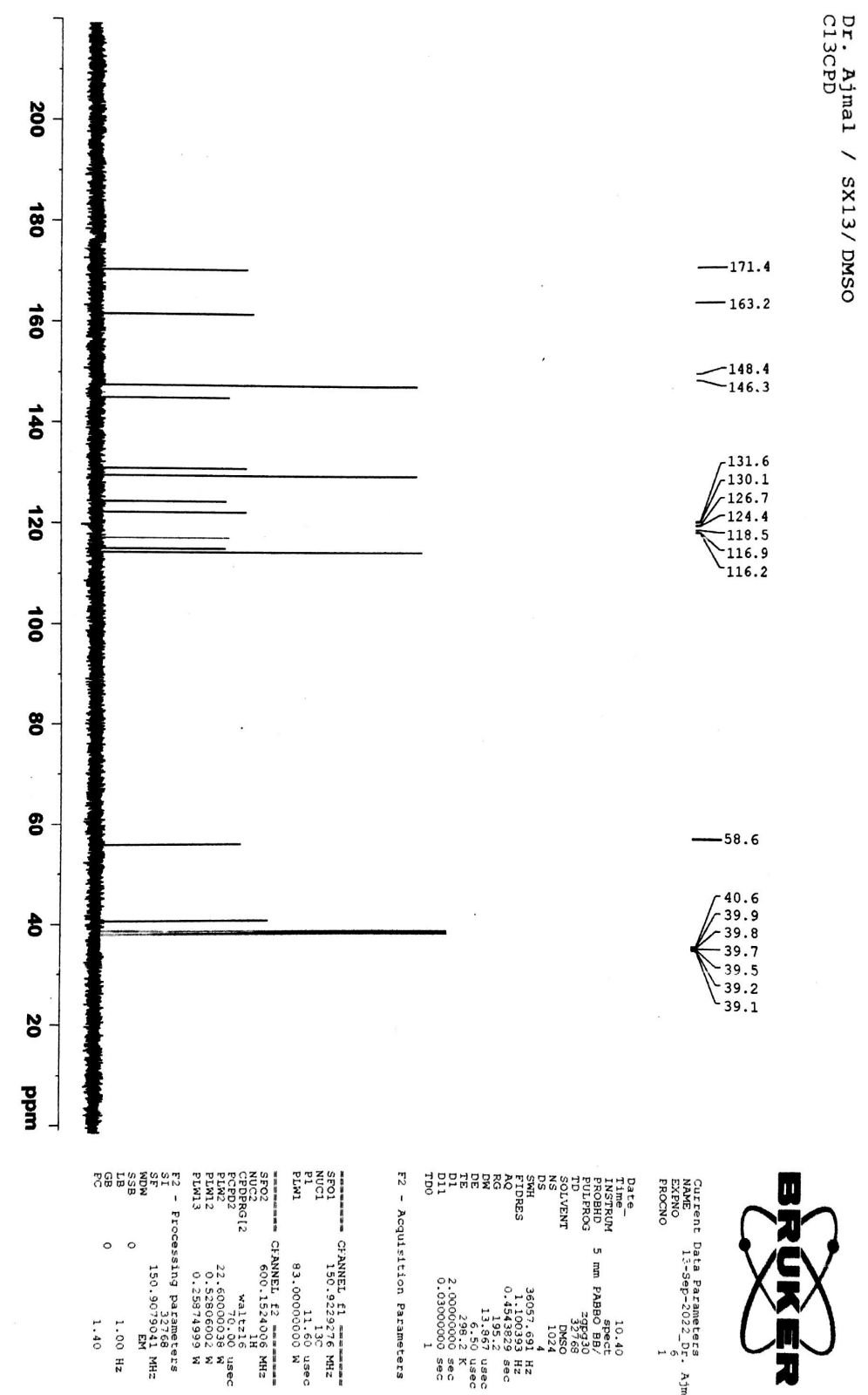

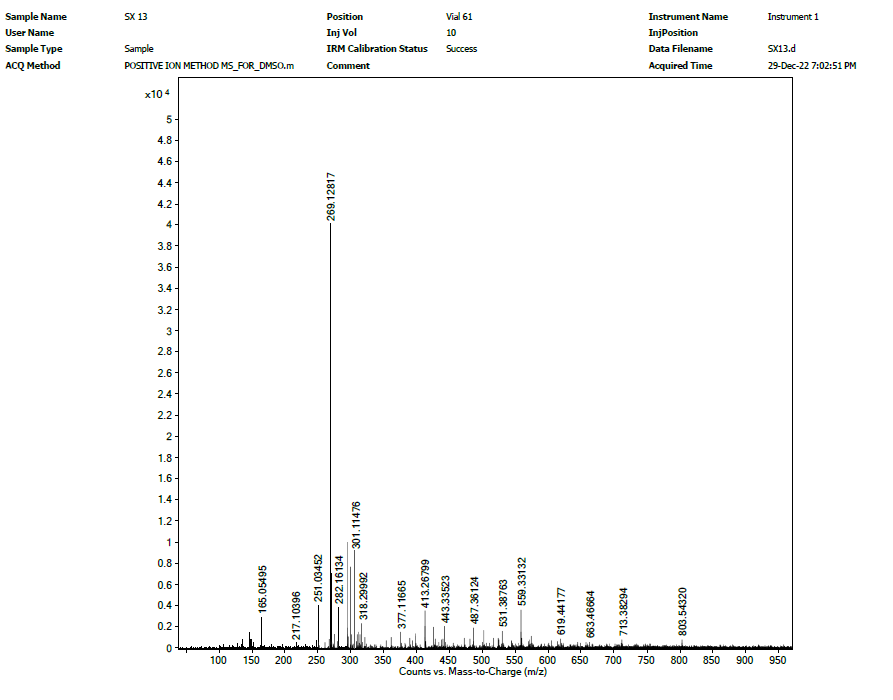
 **Fig S13:** ^1^H-, ^13^C-NMR and HR-ESI-MS spectra of compound **13**


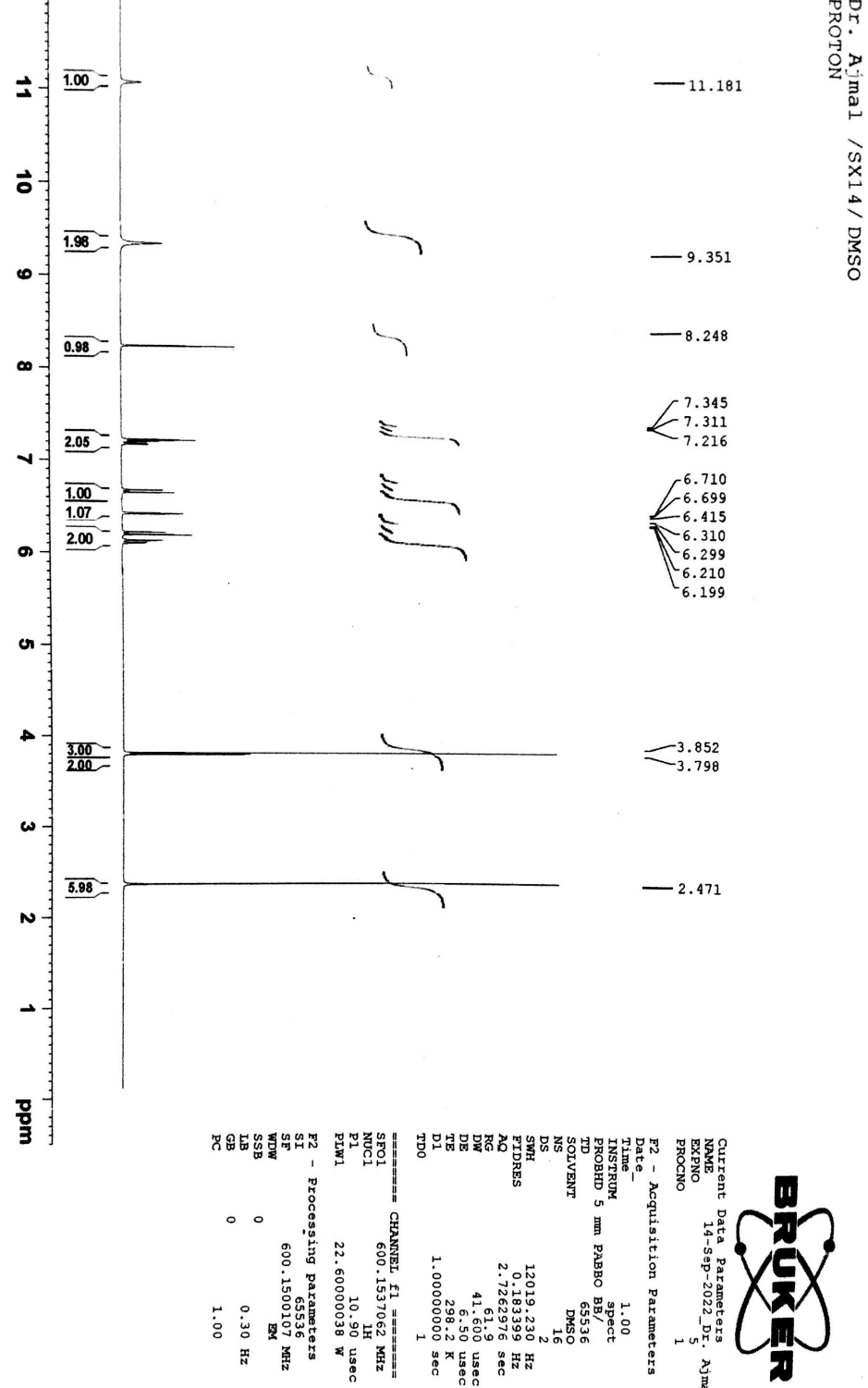


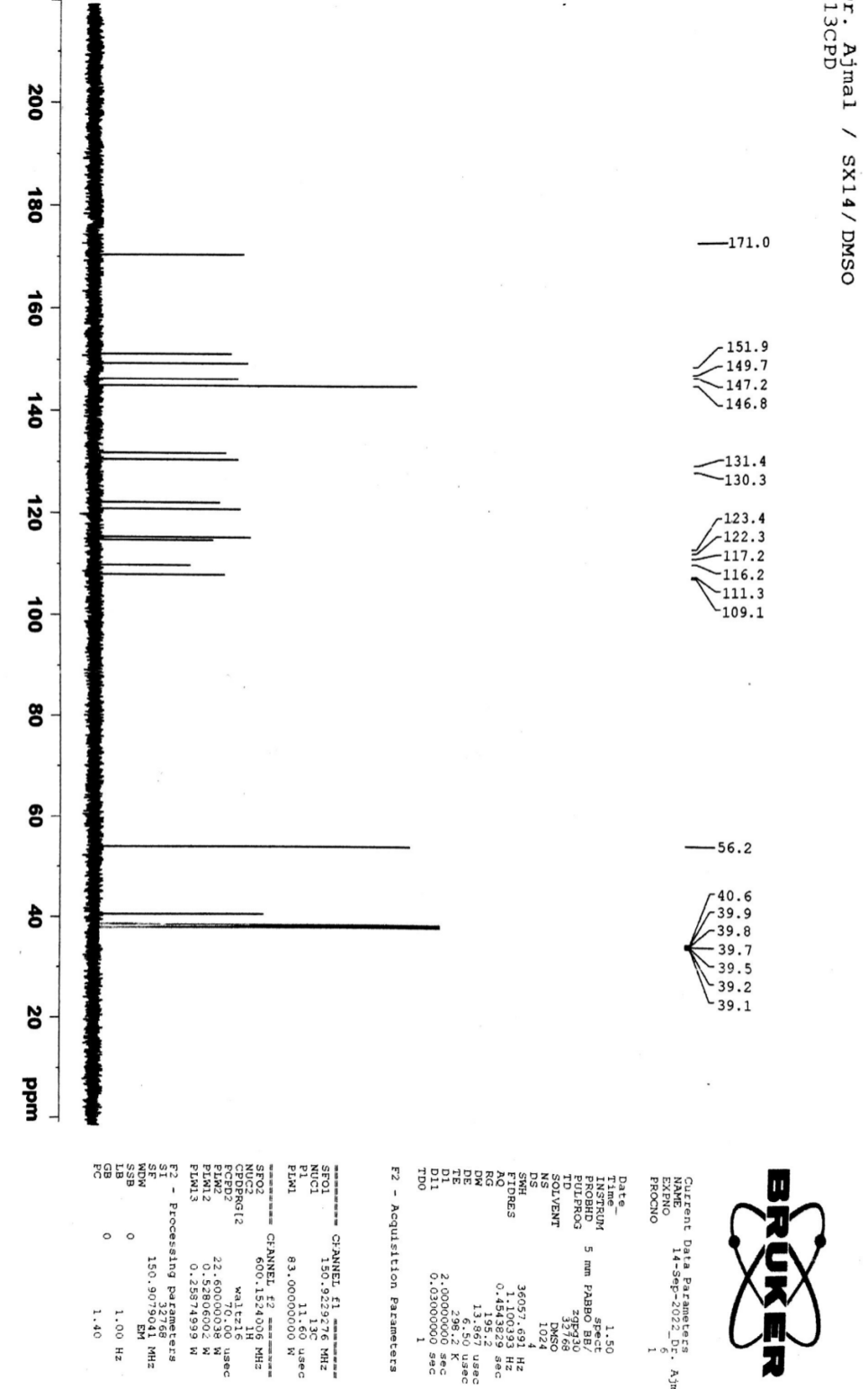


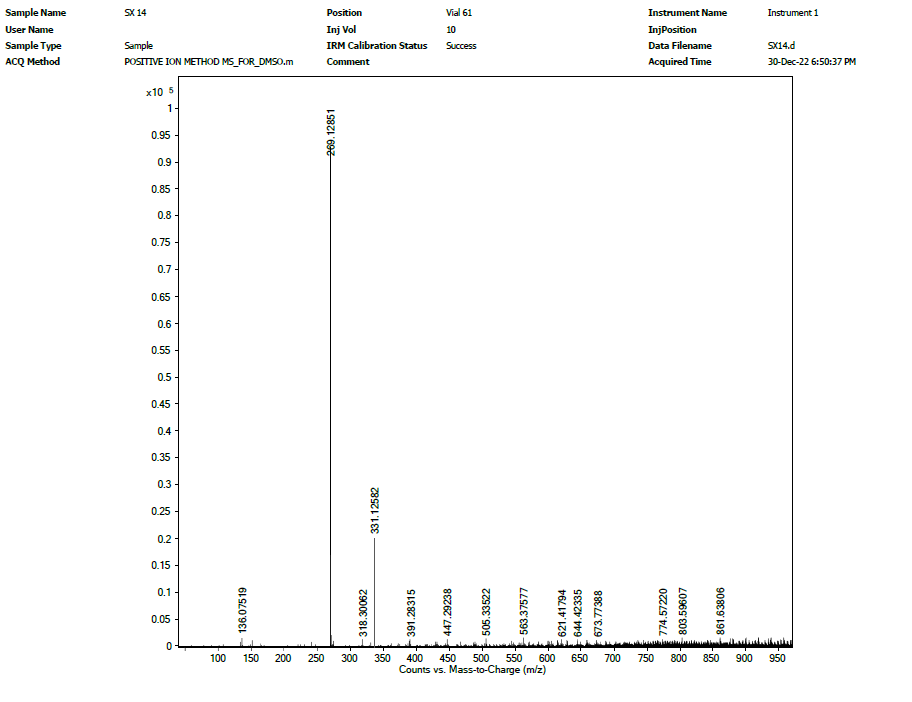


**Fig S14:** ^1^H-, ^13^C-NMR and HR-ESI-MS spectra of compound **14**


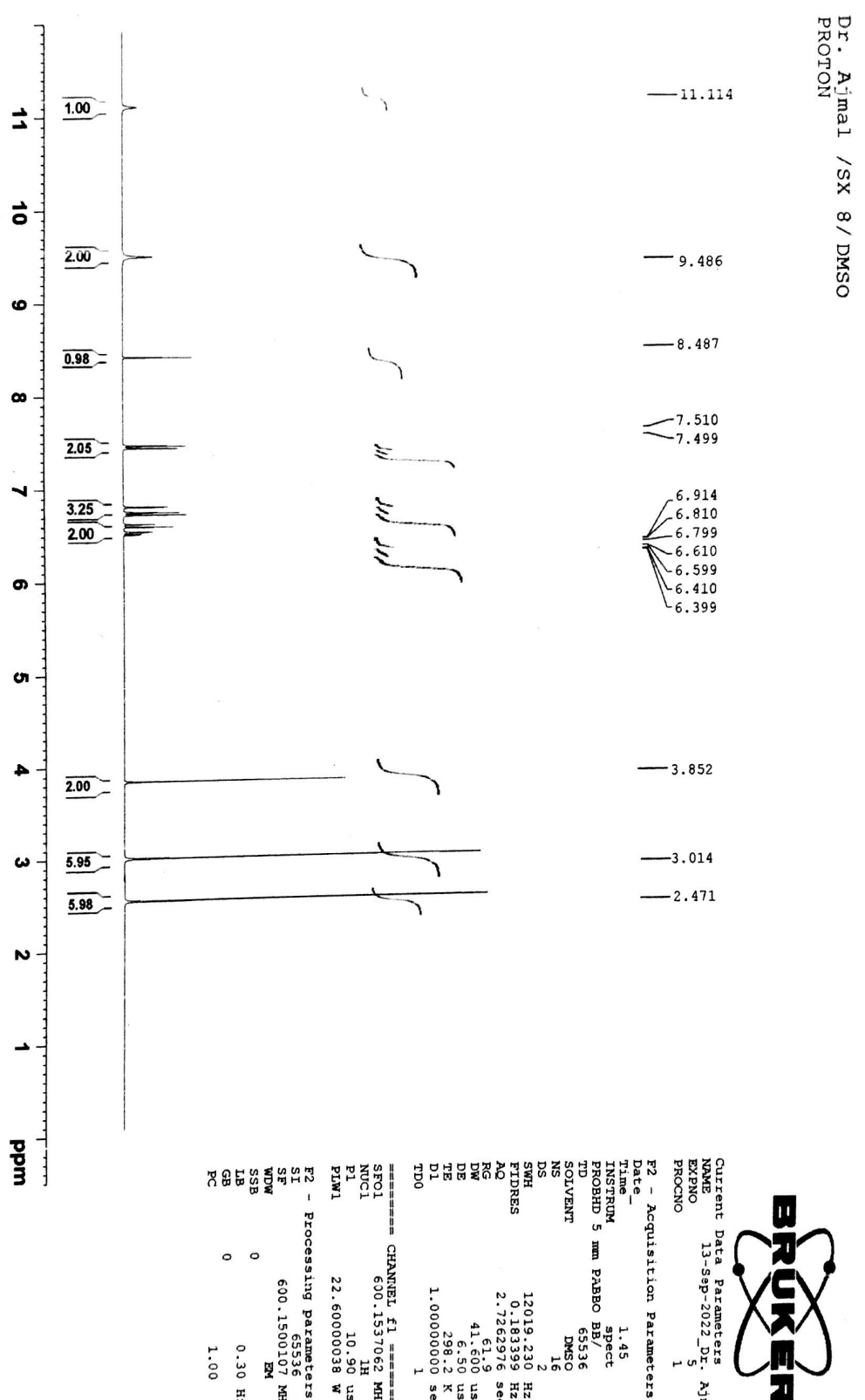


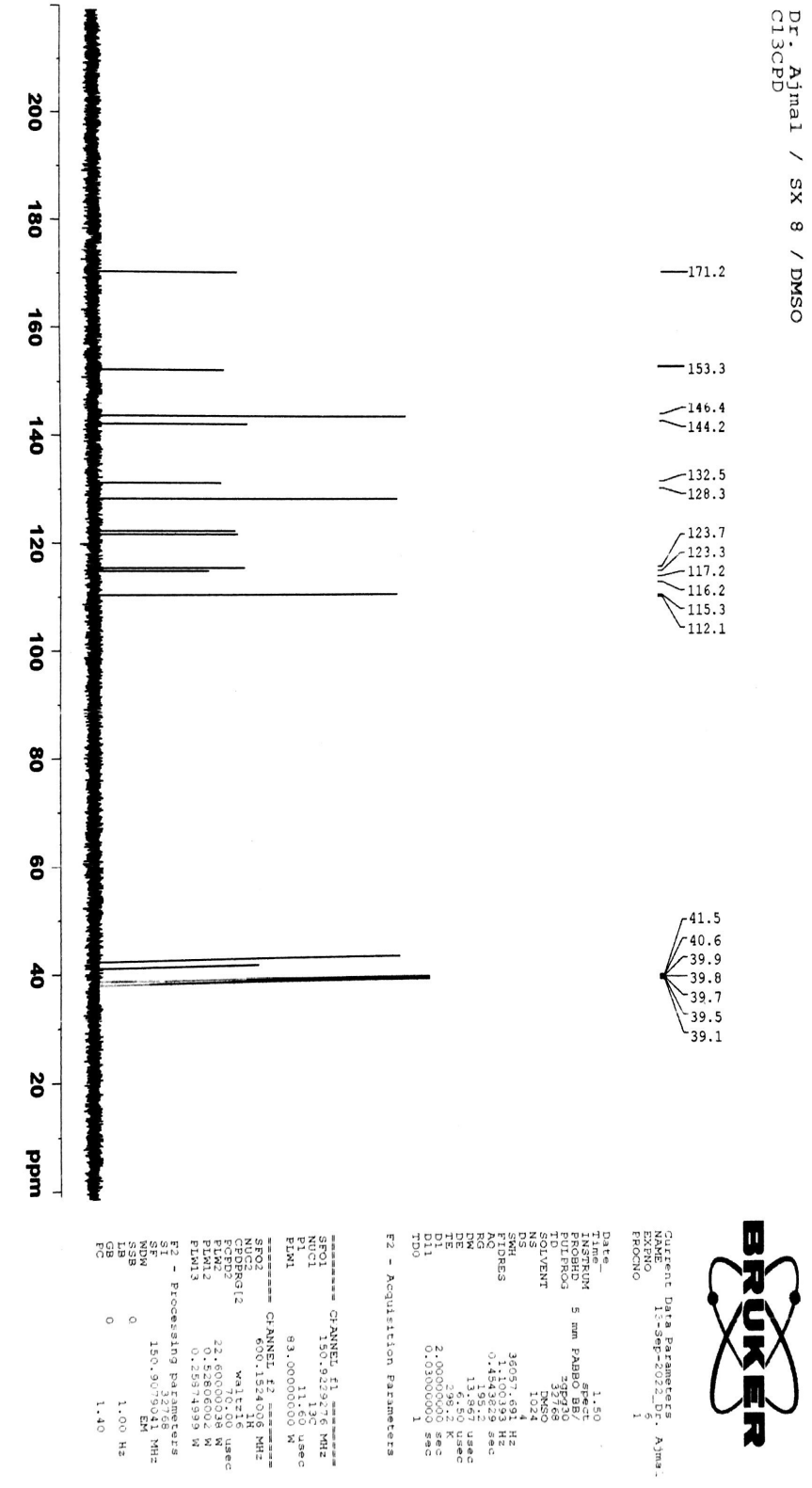


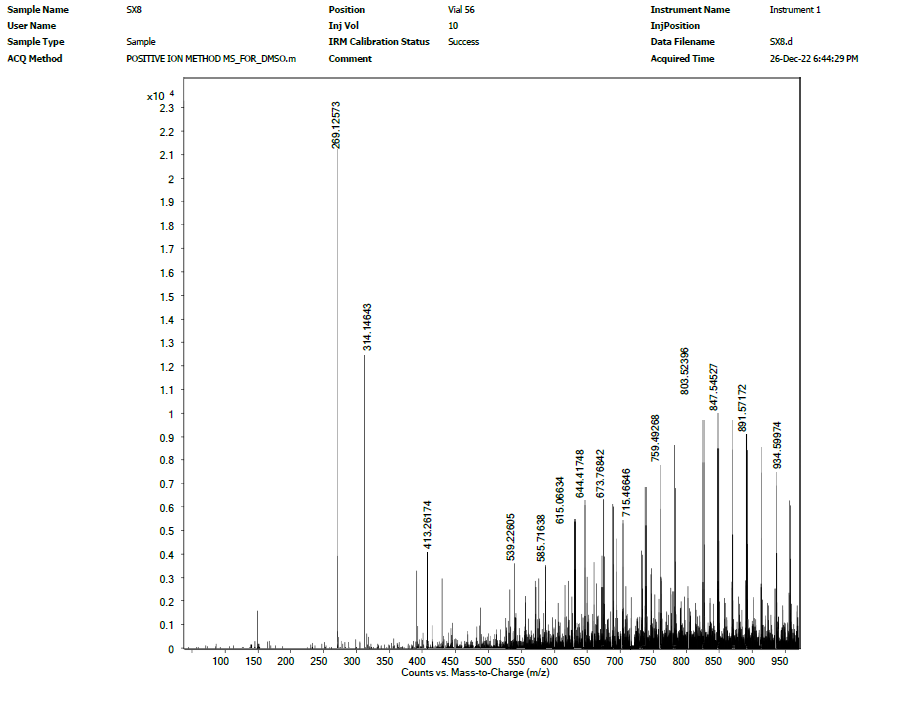
 **Fig S15:** ^1^H-, ^13^C-NMR and HR-ESI-MS spectra of compound **15**


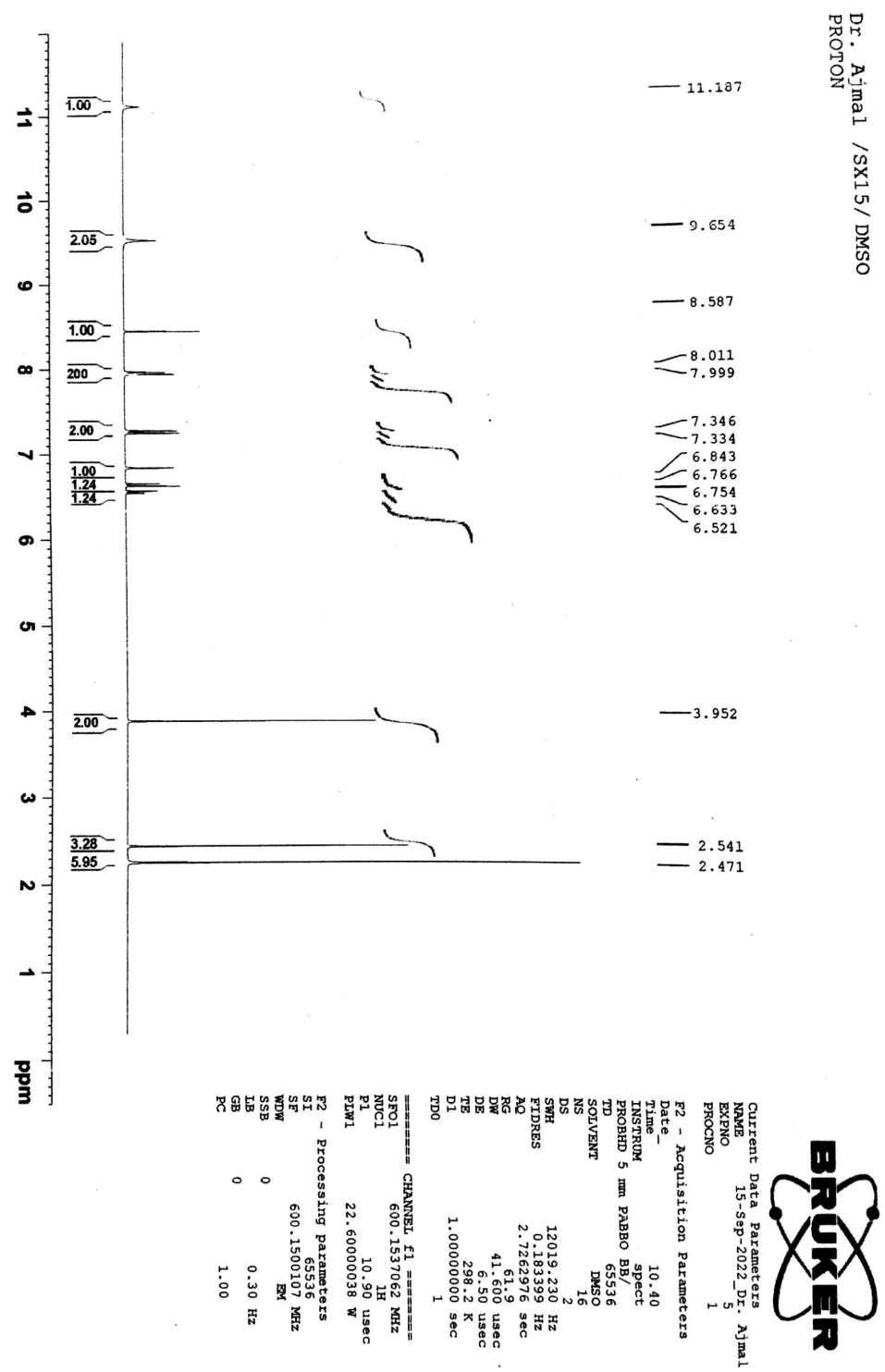


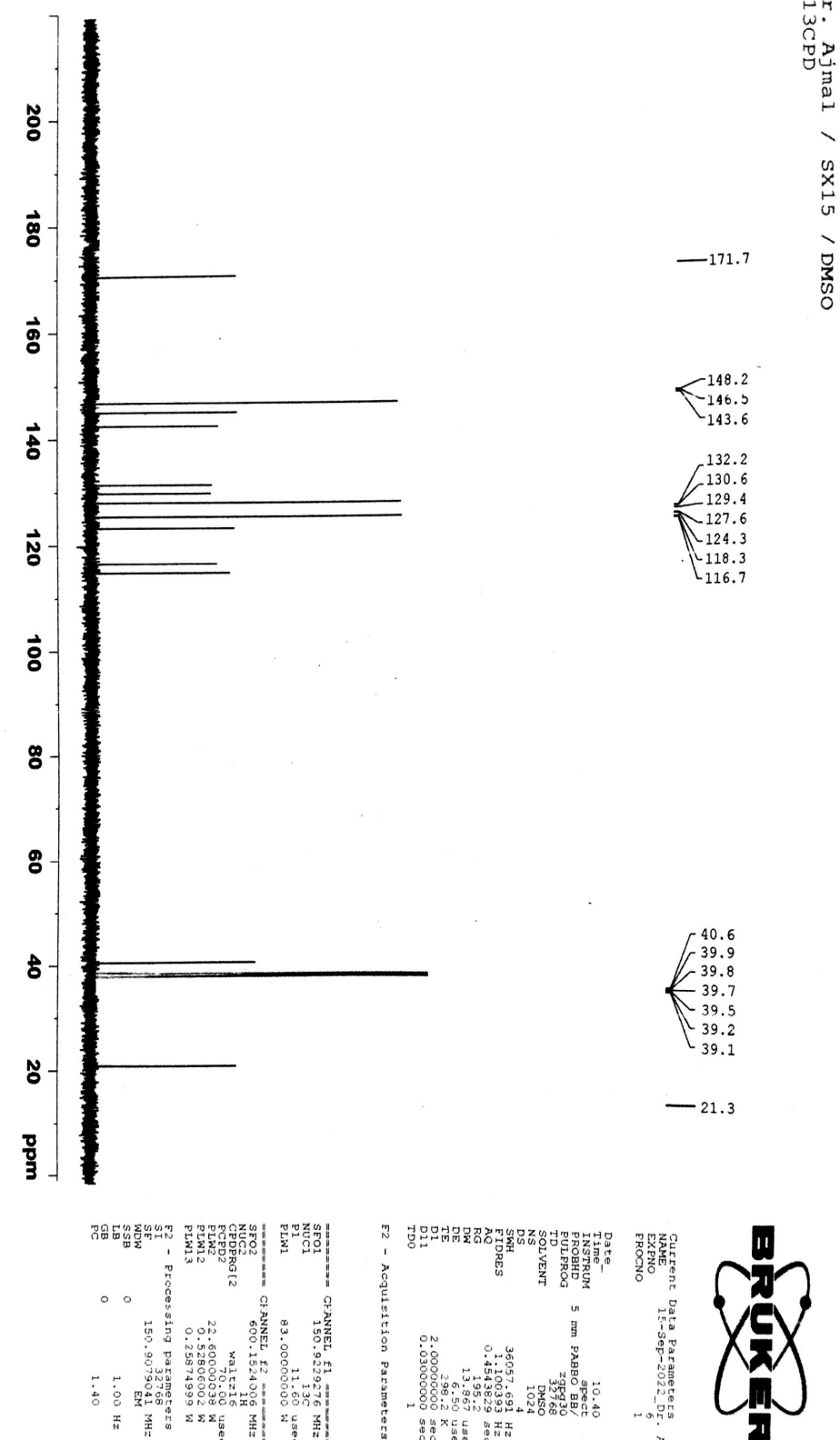


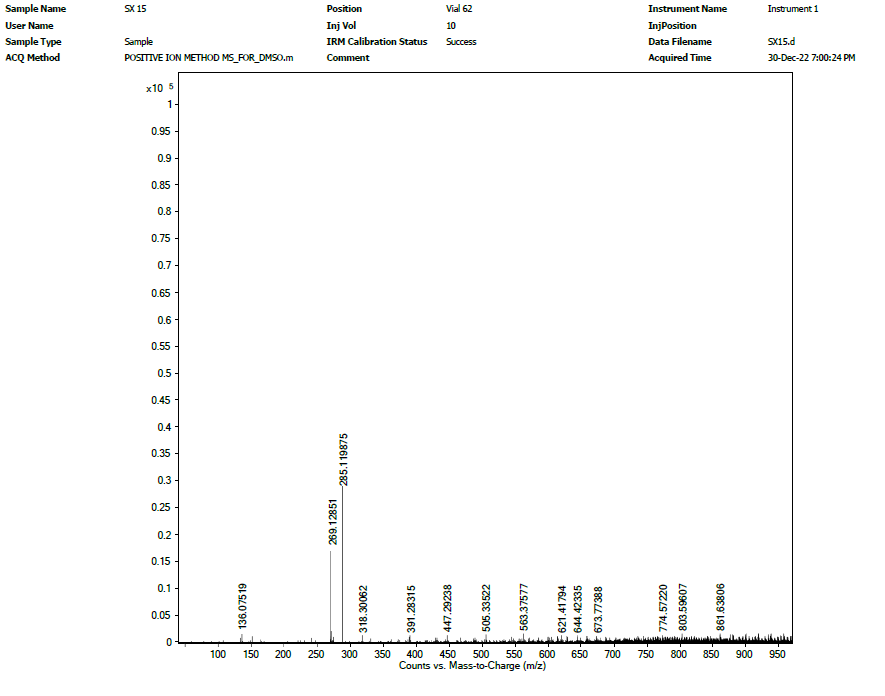


**Fig S16:** ^1^H-, ^13^C-NMR and HR-ESI-MS spectra of compound **16**


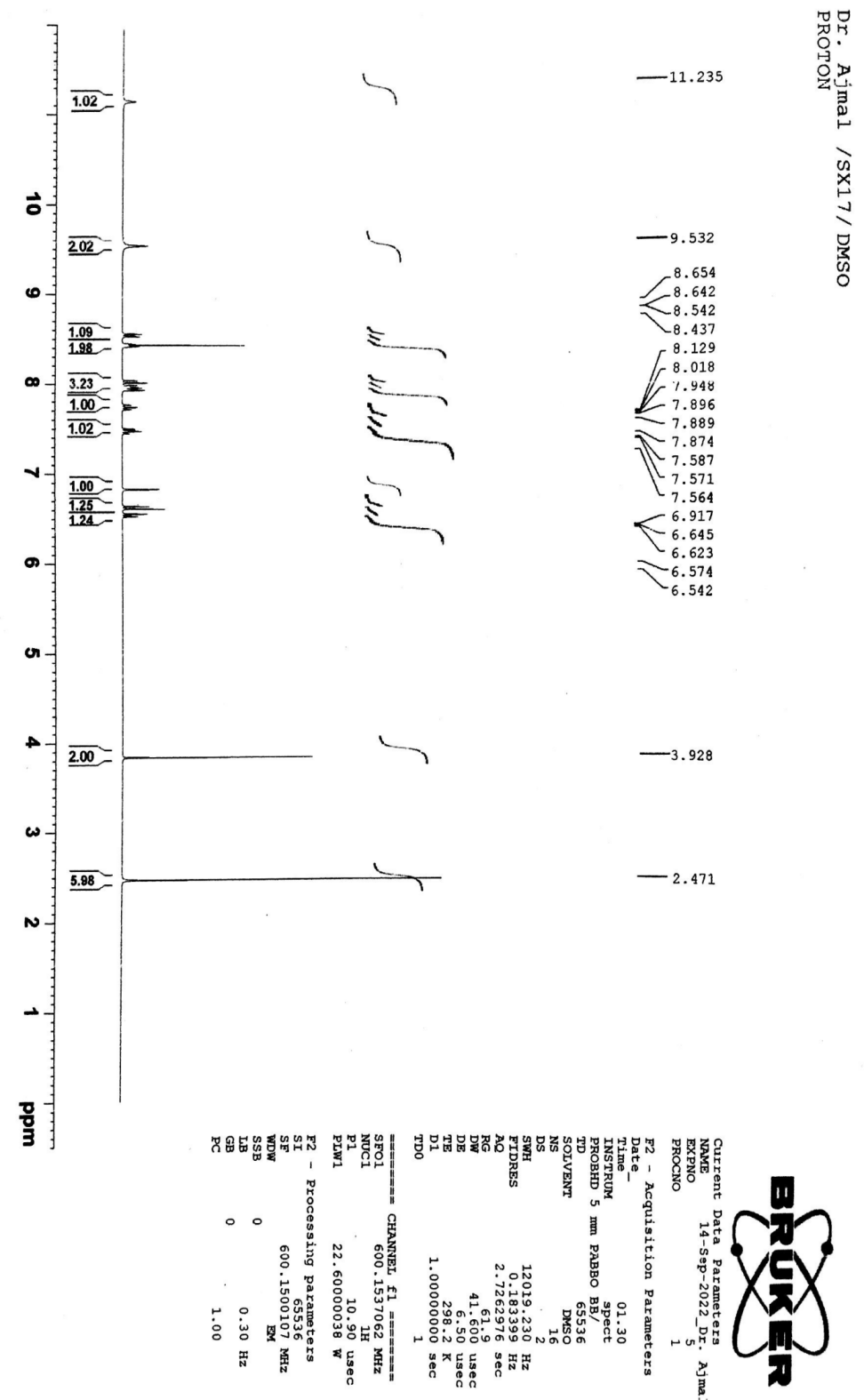


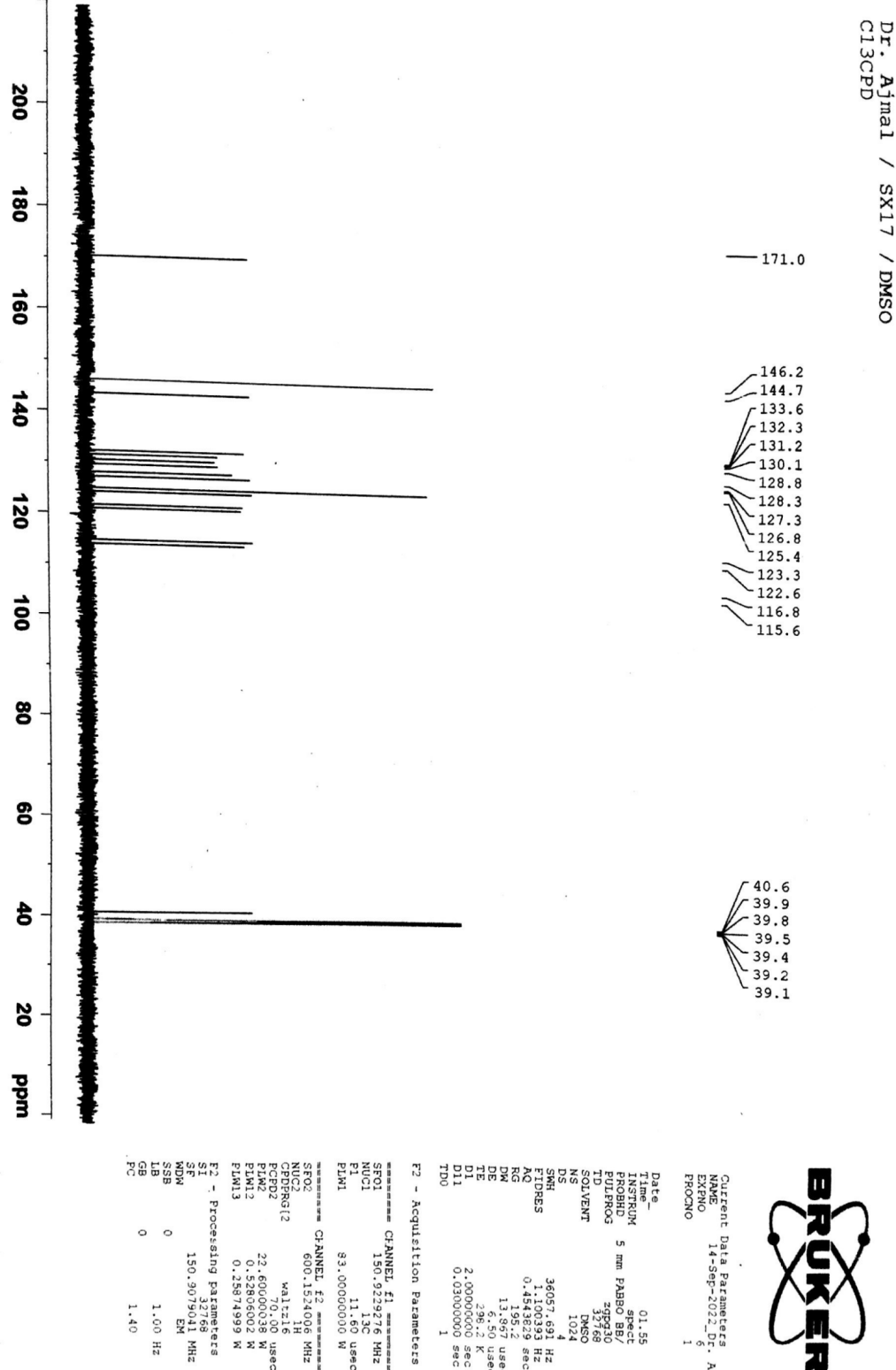


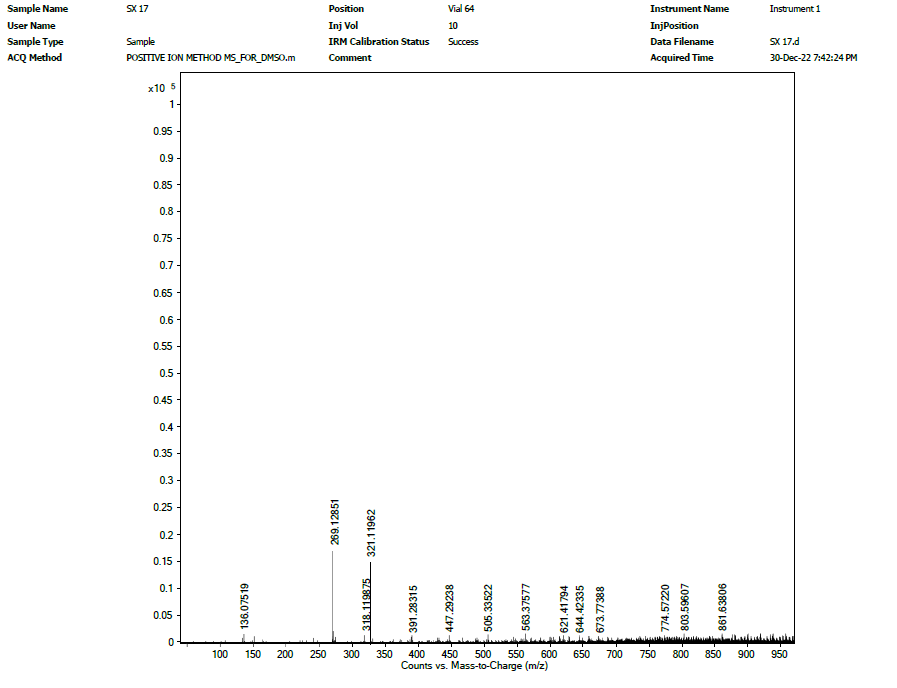
 **Fig S17:** ^1^H-, ^13^C-NMR and HR-ESI-MS spectra of compound **17**

**
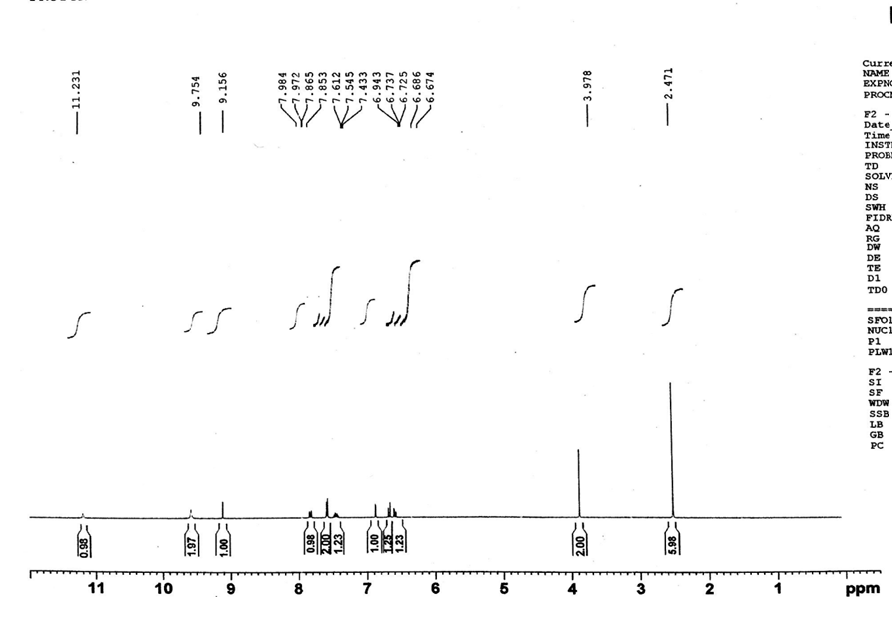
**

**
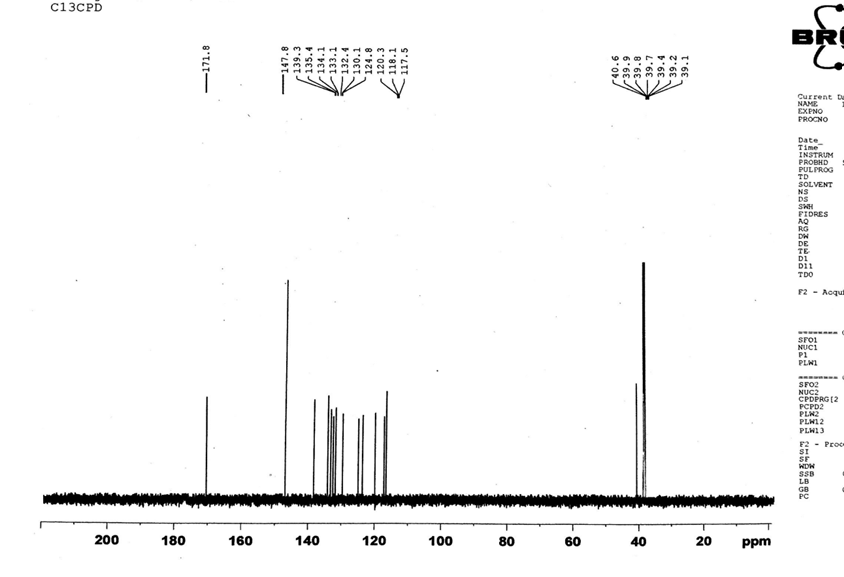
**


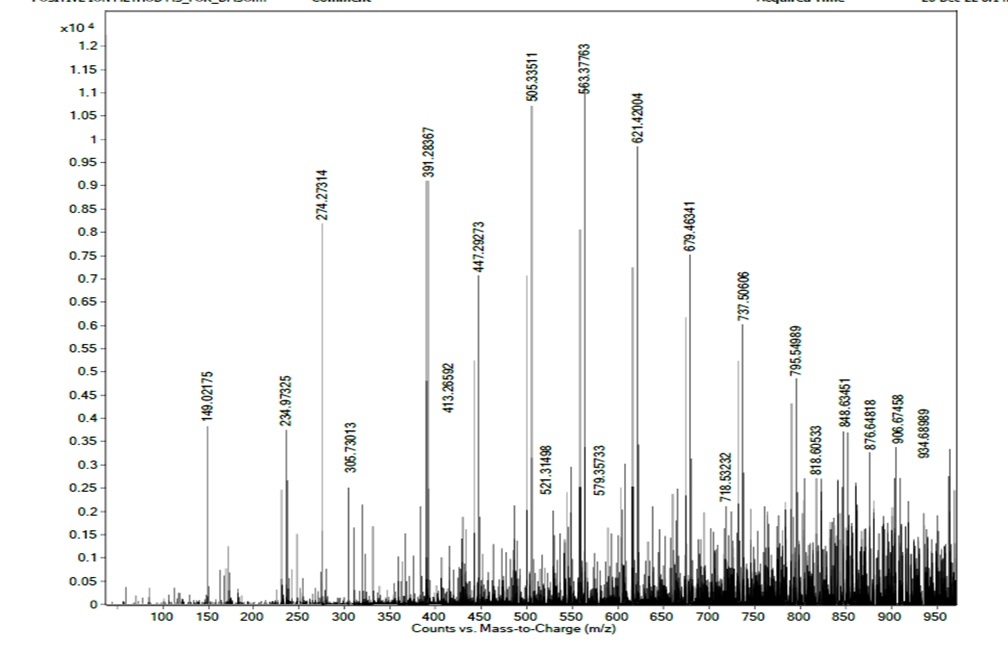


**Fig S18:** ^1^H-, ^13^C-NMR and HR-ESI-MS spectra of compound **18**


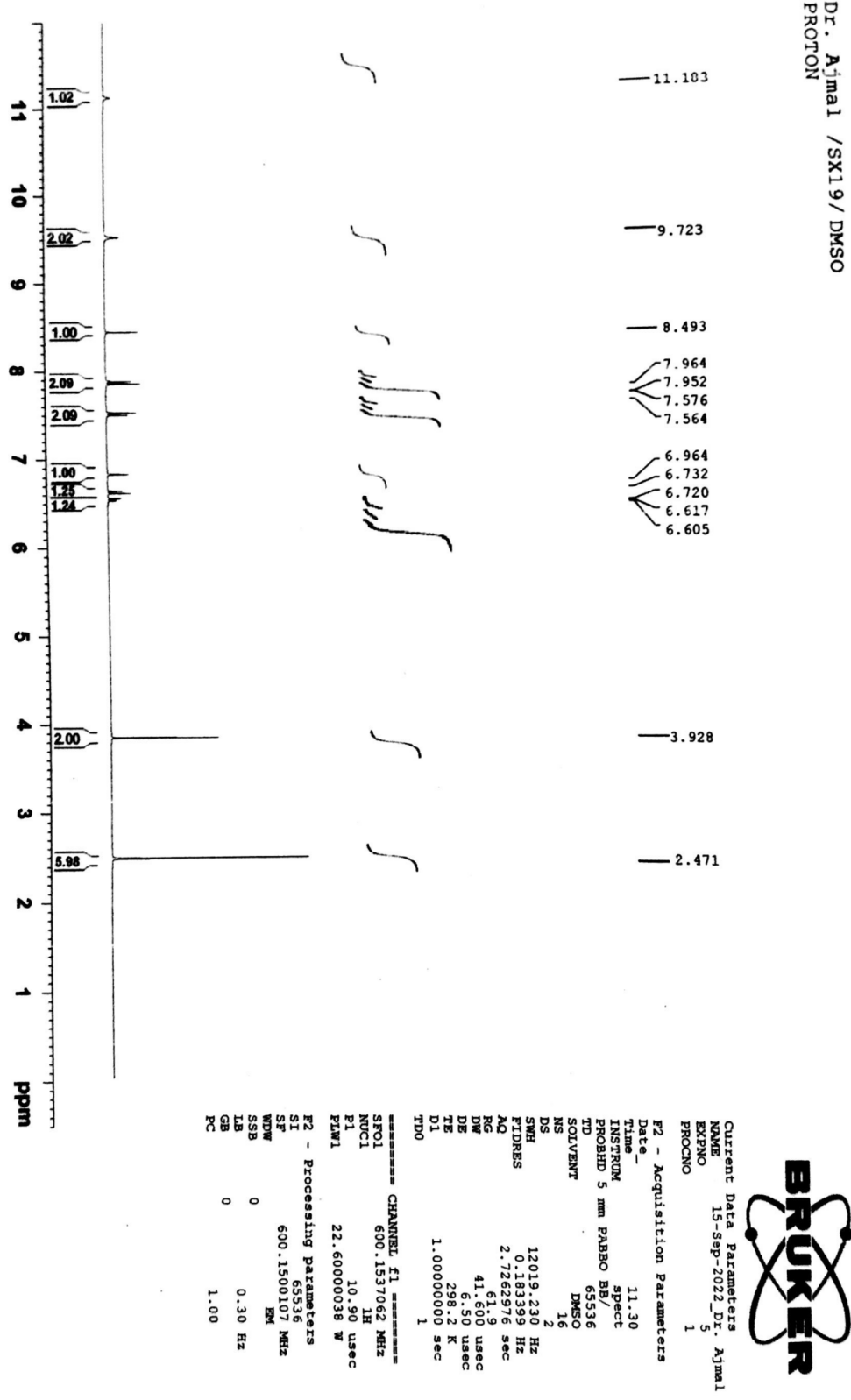


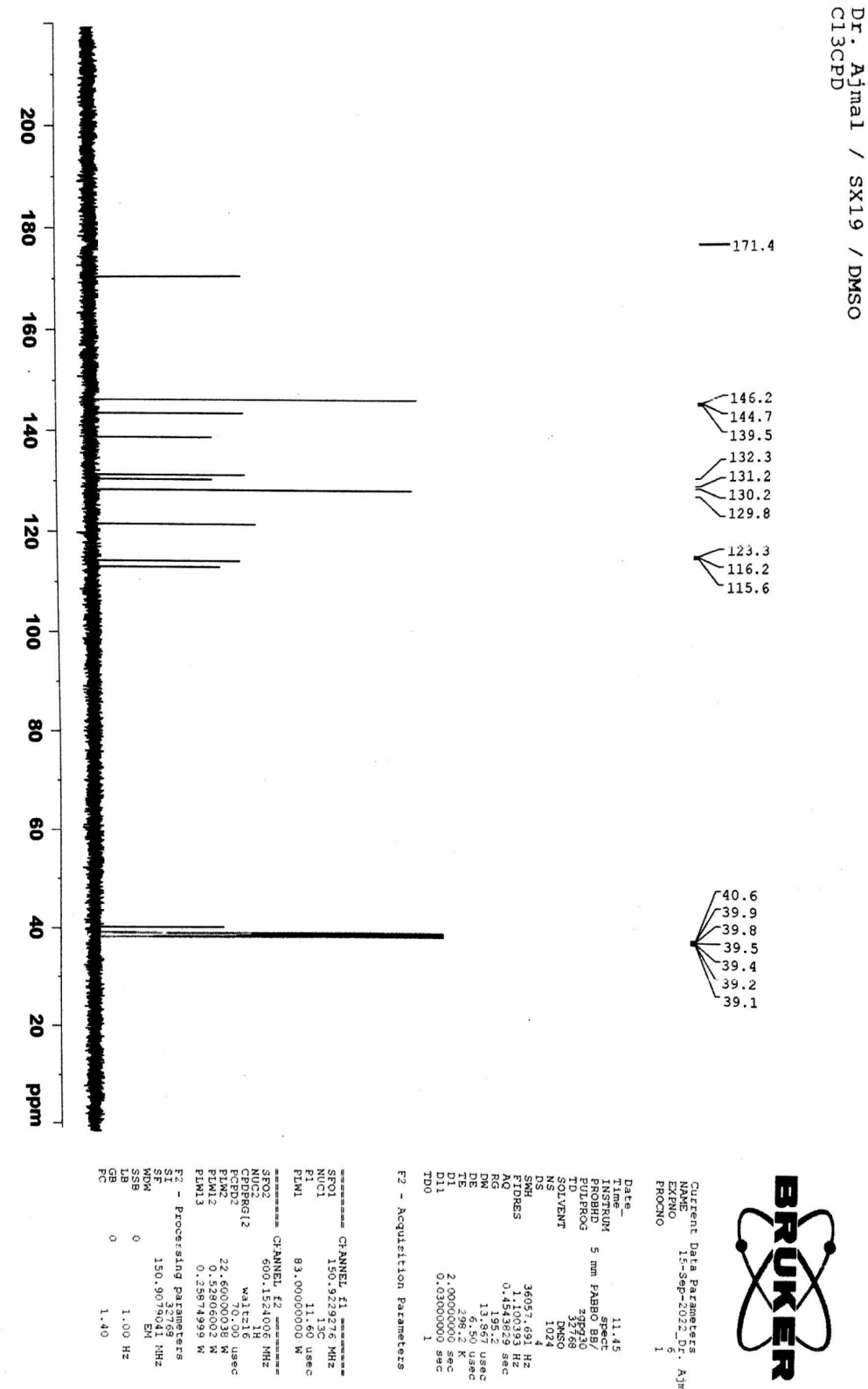


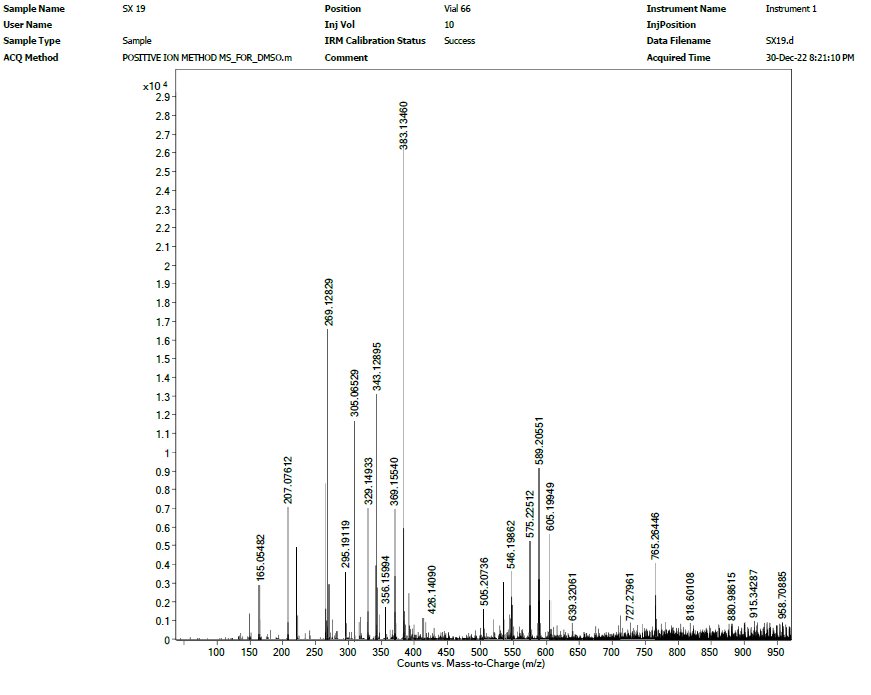


**Fig S19:** ^1^H-, ^13^C-NMR and HR-ESI-MS spectra of compound **19**


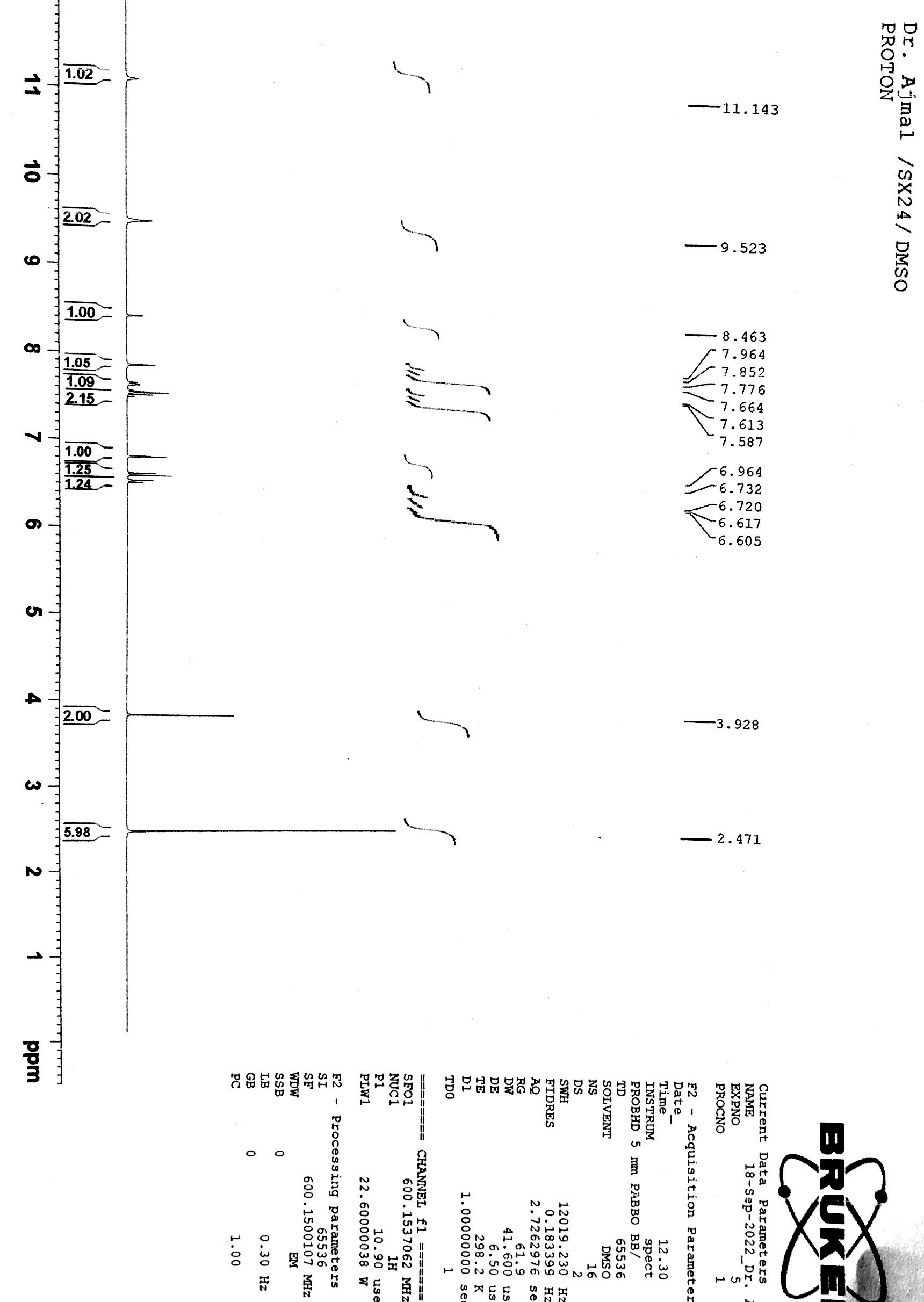


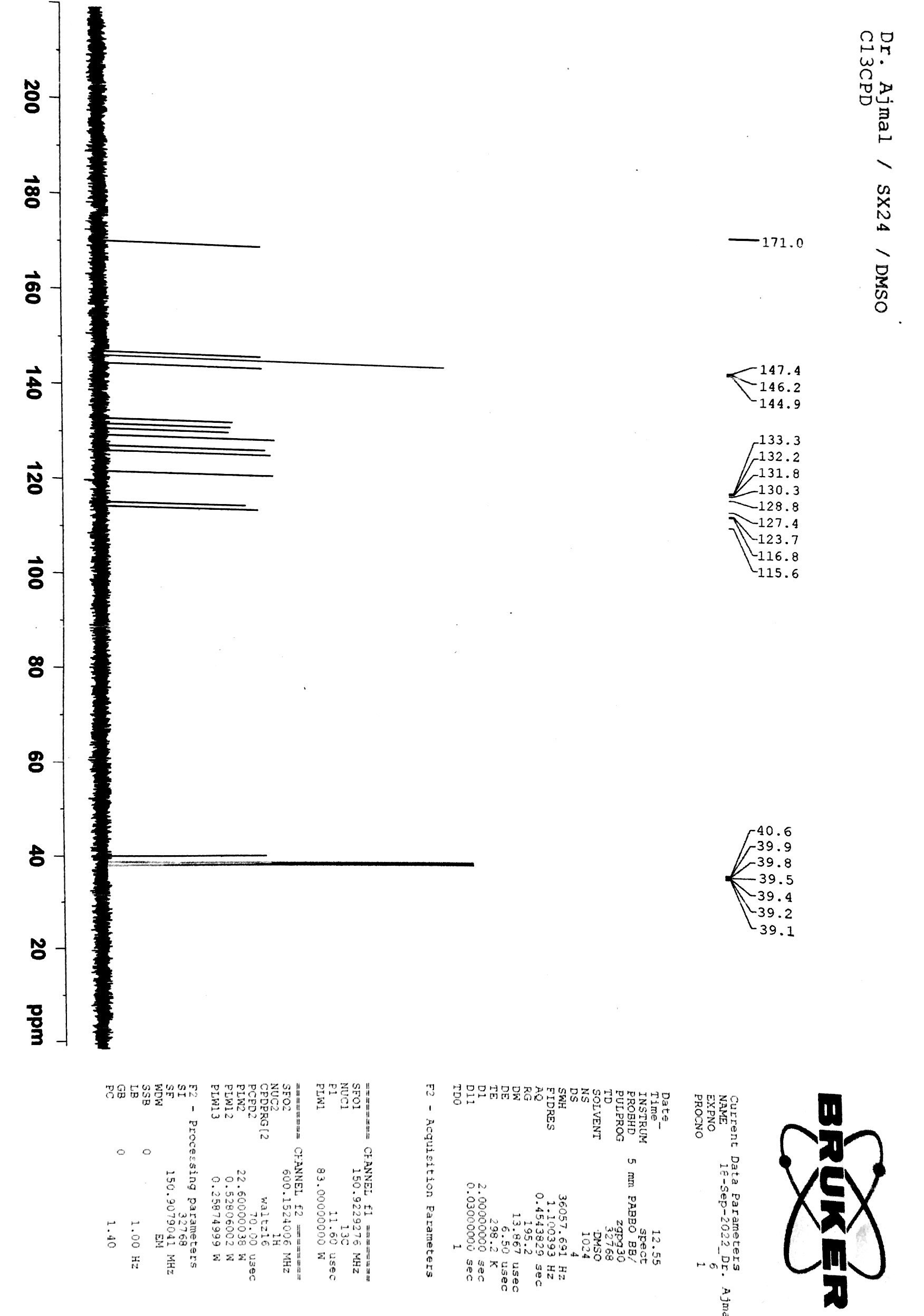


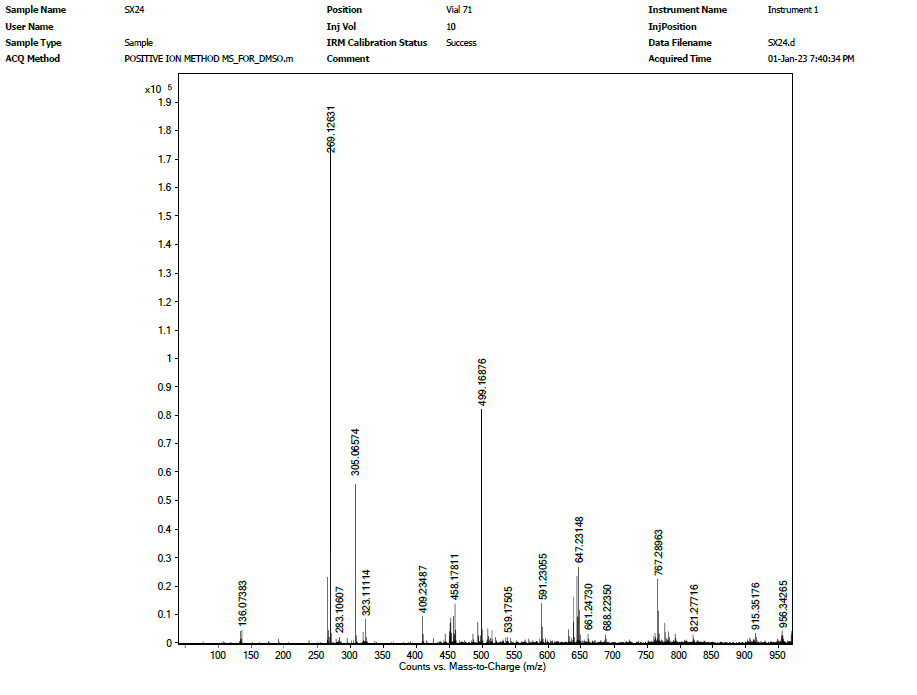


**Fig S20:** ^1^H-, ^13^C-NMR and HR-ESI-MS spectra of compound **20**


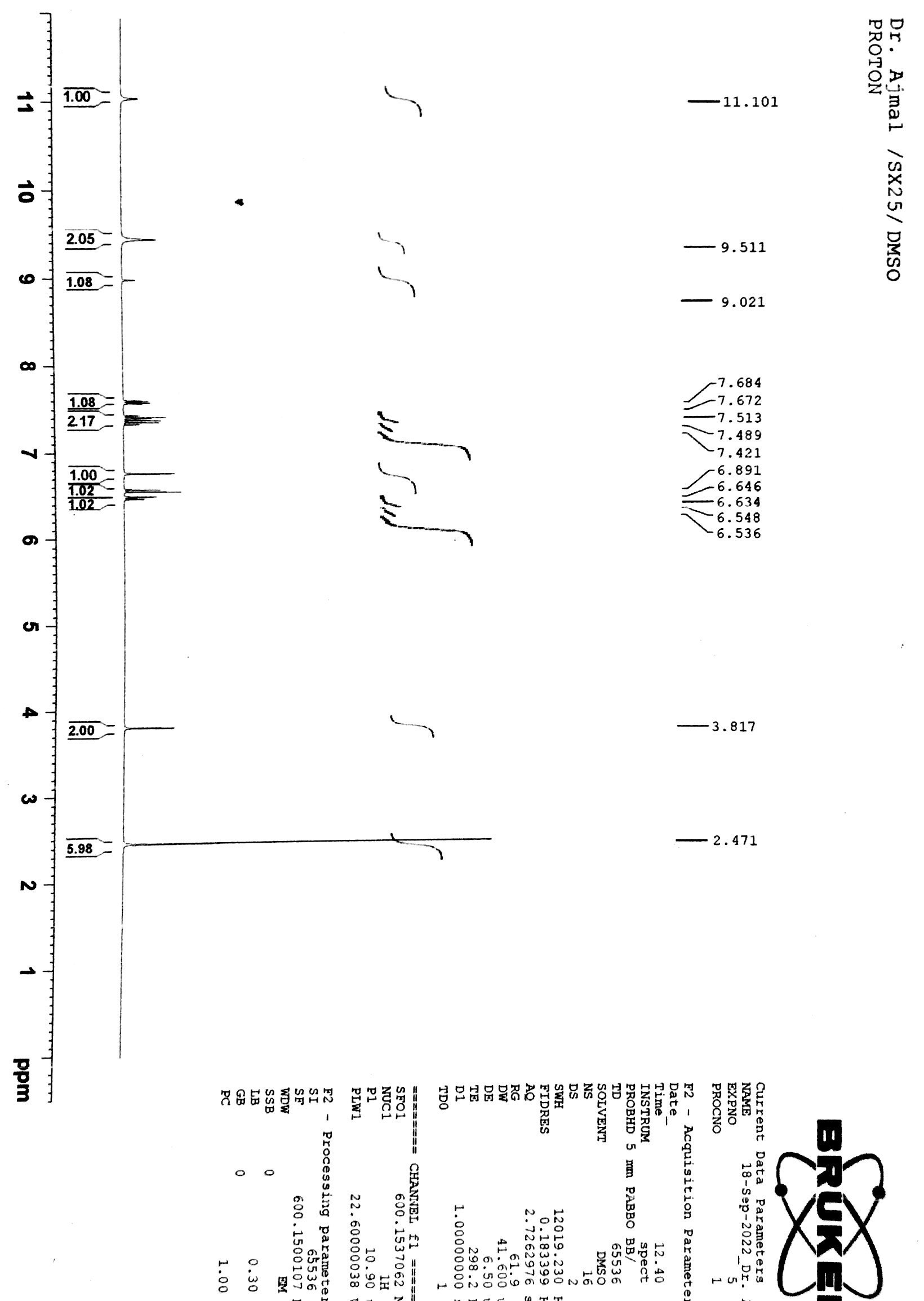


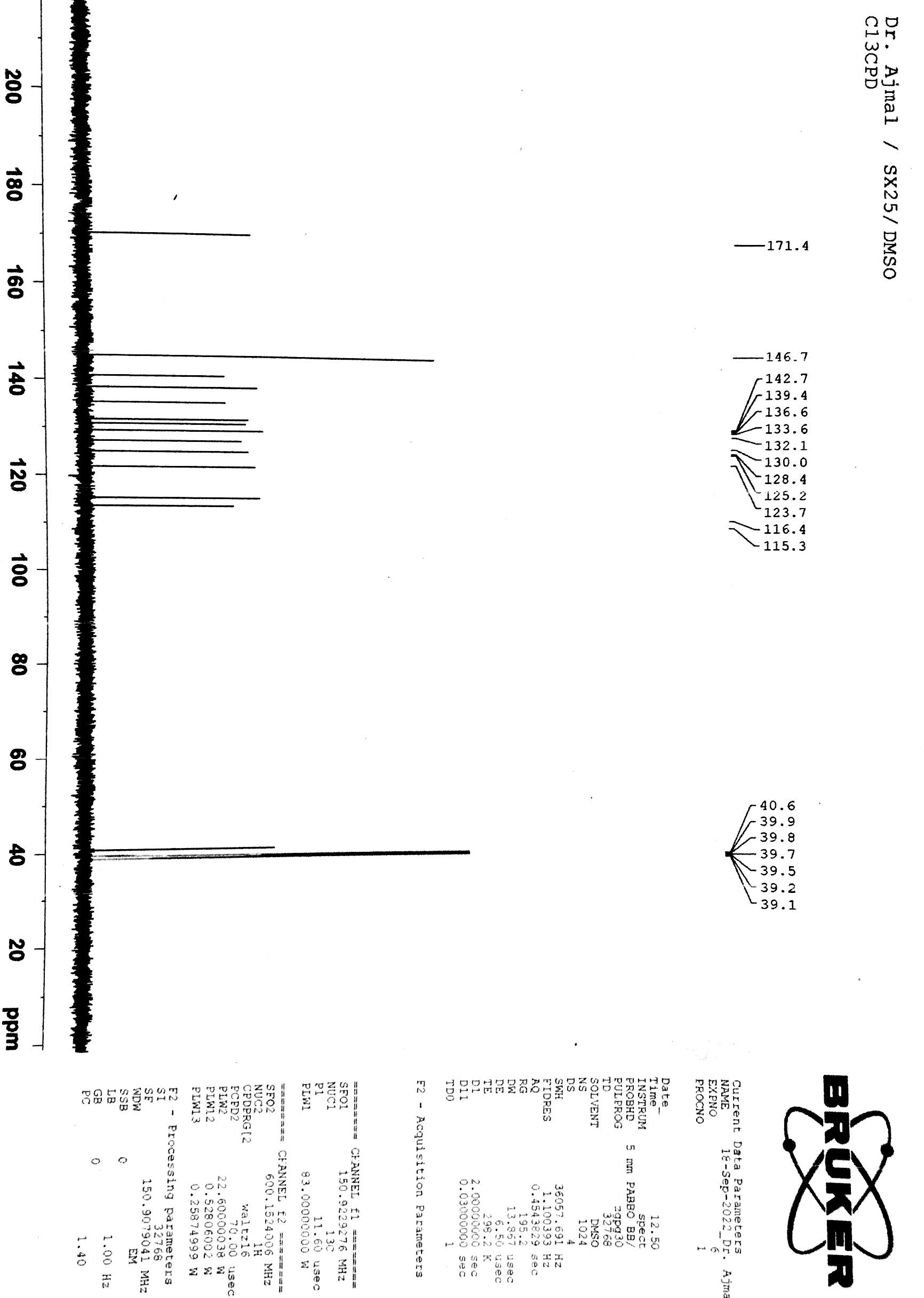


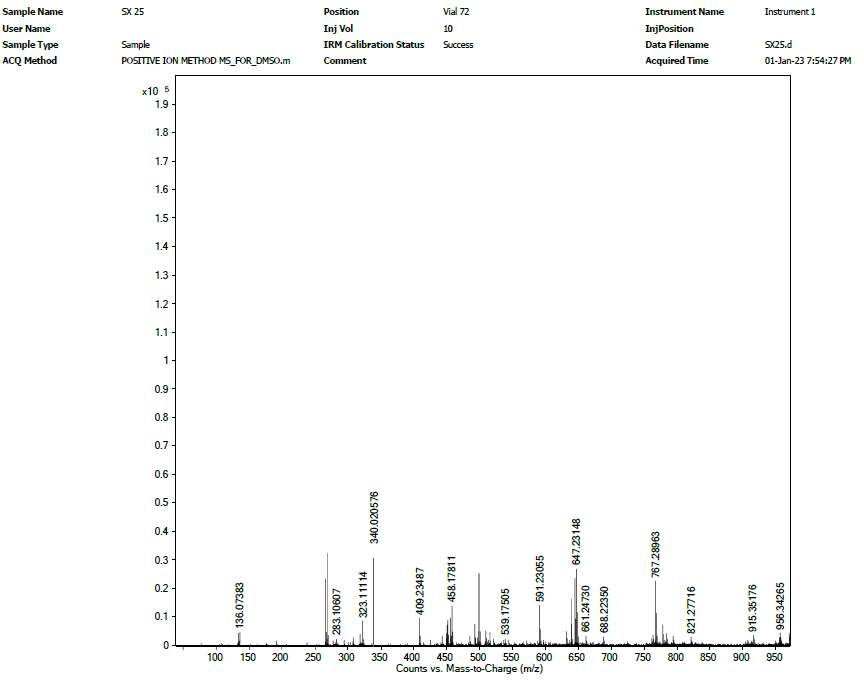


**Fig S21:** ^1^H-, ^13^C-NMR and HR-ESI-MS spectra of compound **21**


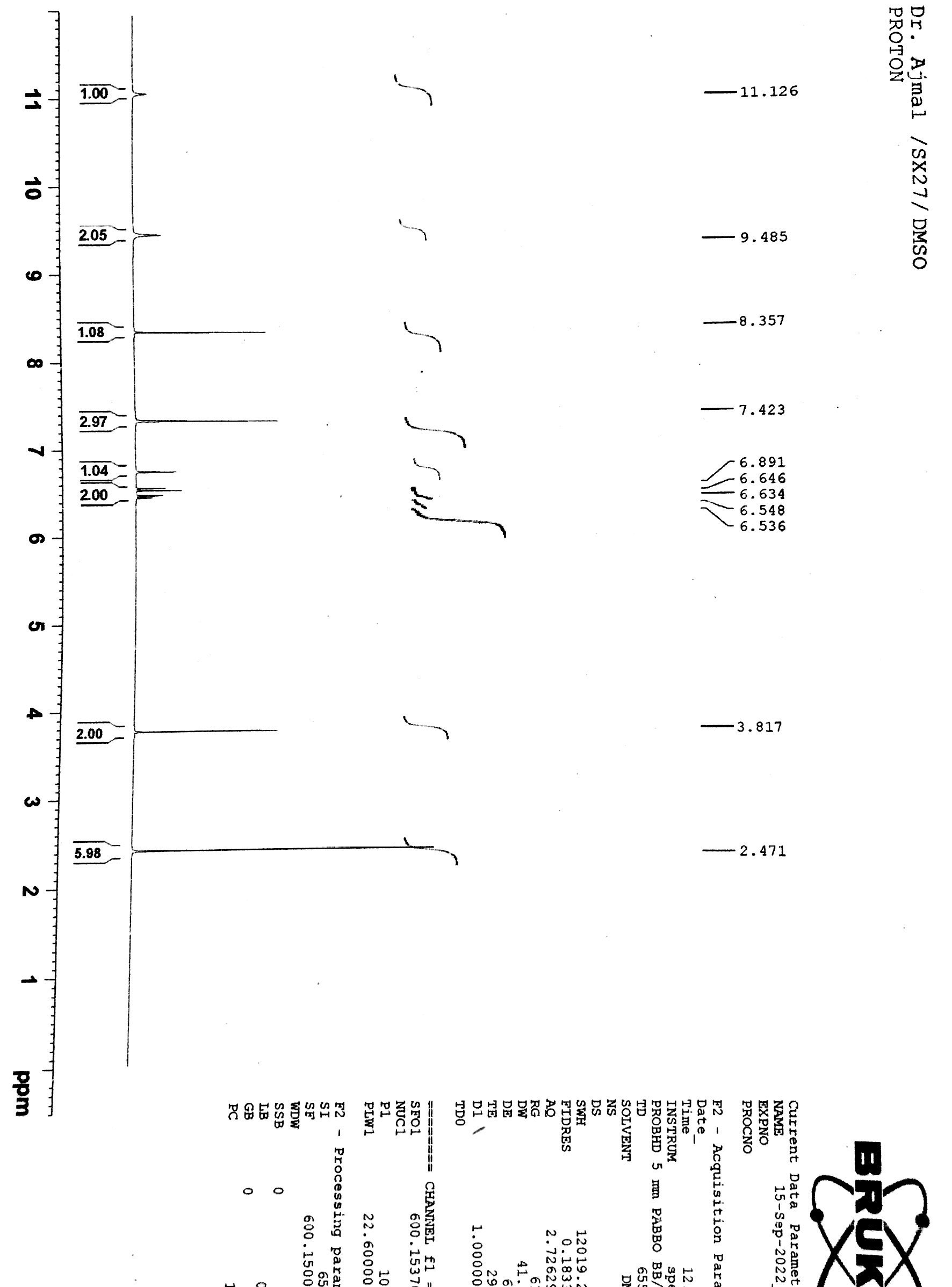


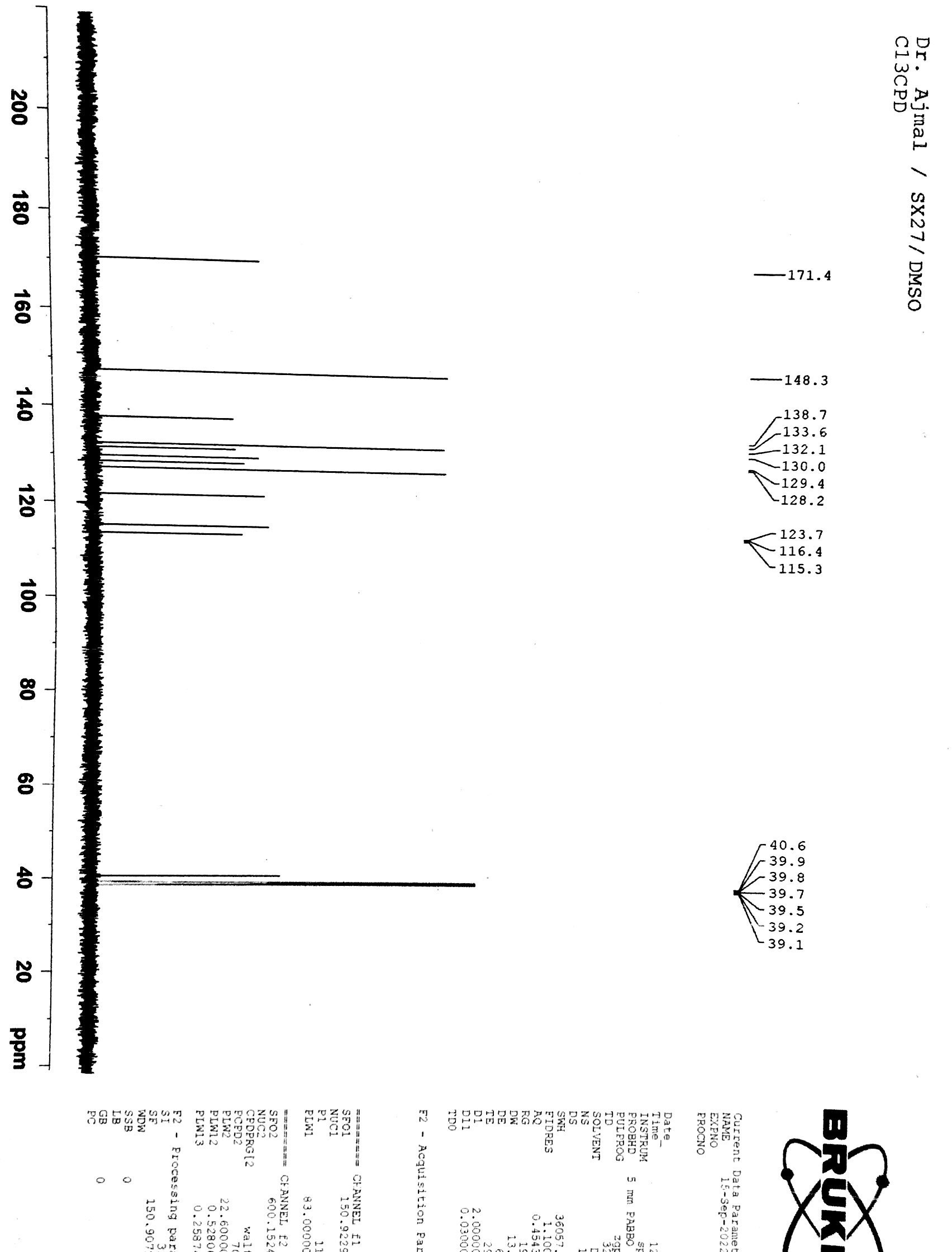


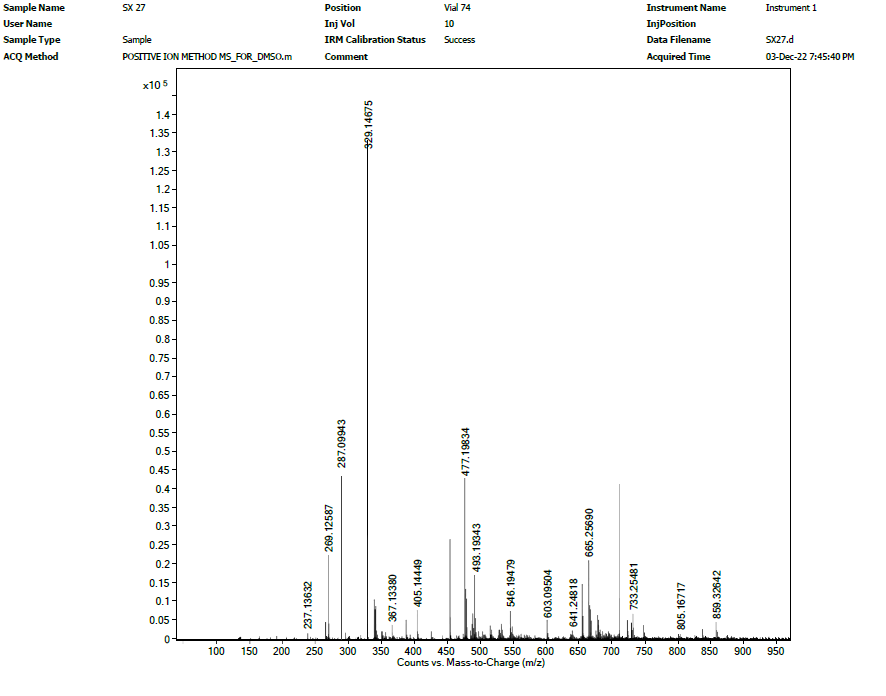


**Fig S22:** ^1^H-, ^13^C-NMR and HR-ESI-MS spectra of compound **22**


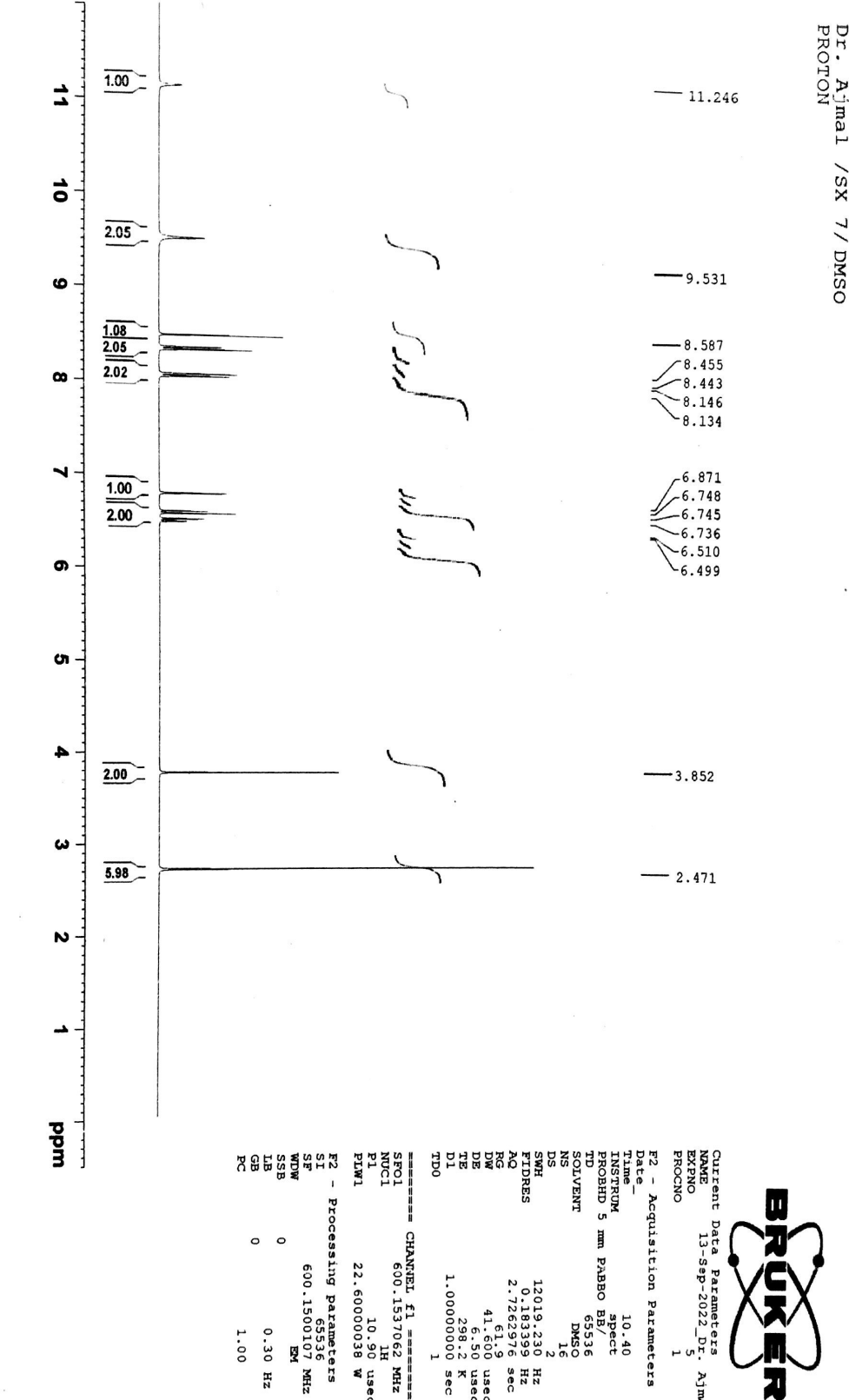


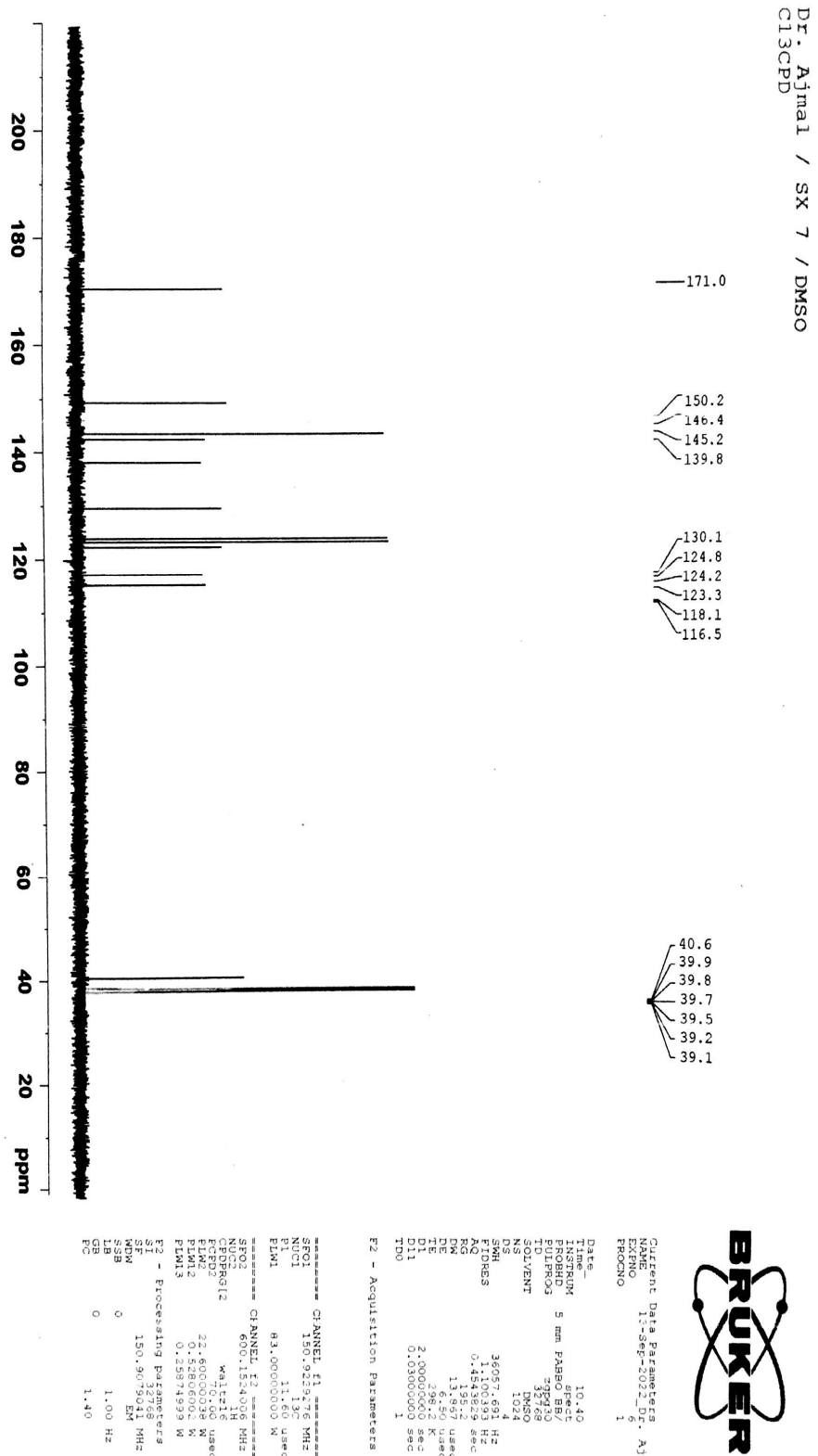


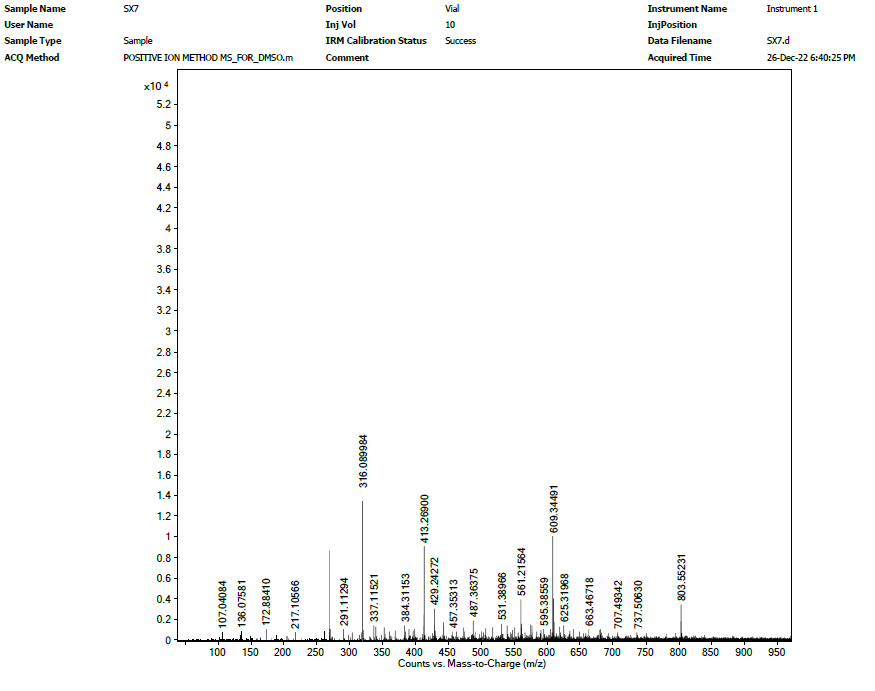
 **Fig S23:** ^1^H-, ^13^C-NMR and HR-ESI-MS spectra of compound **23**


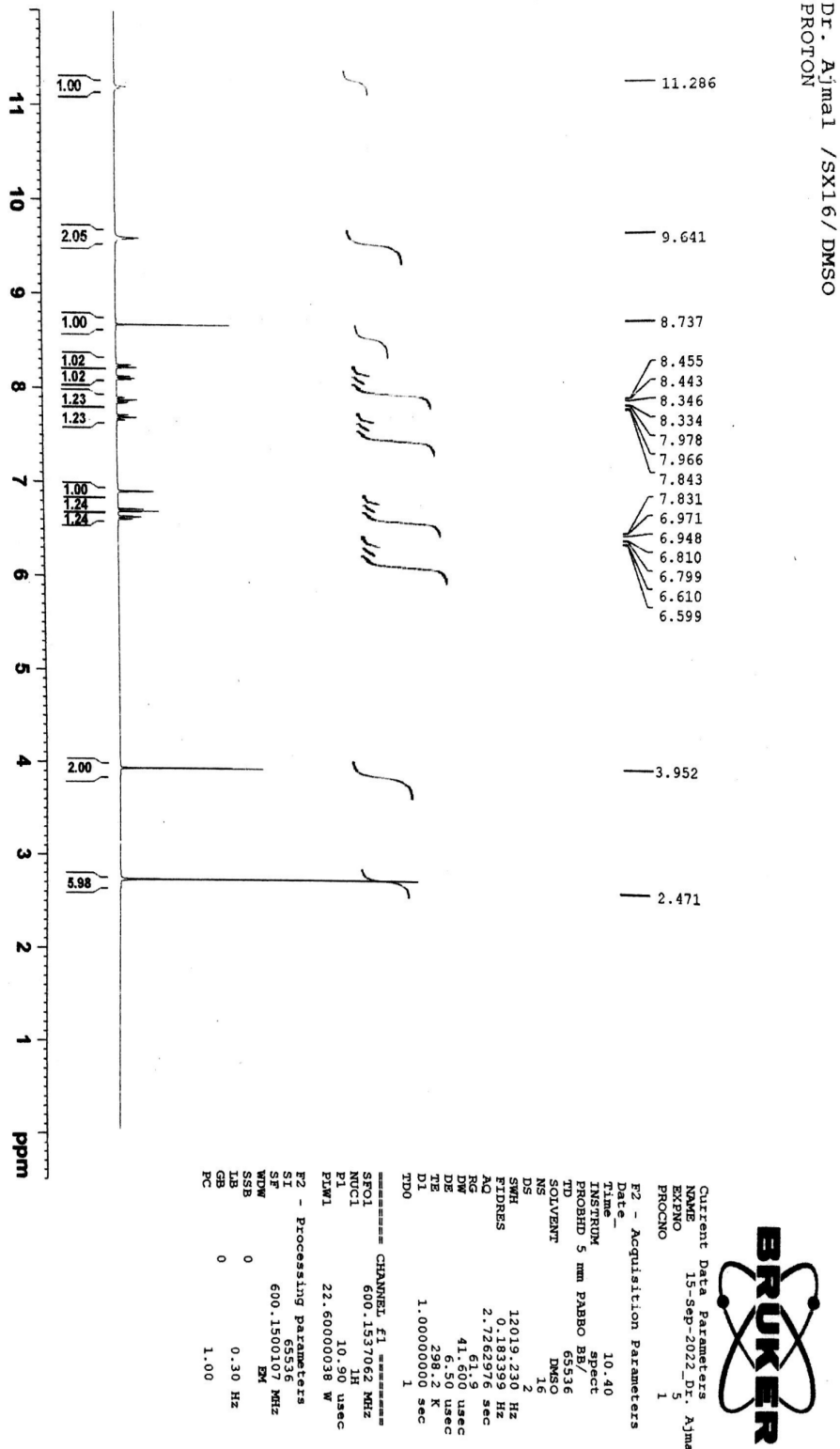


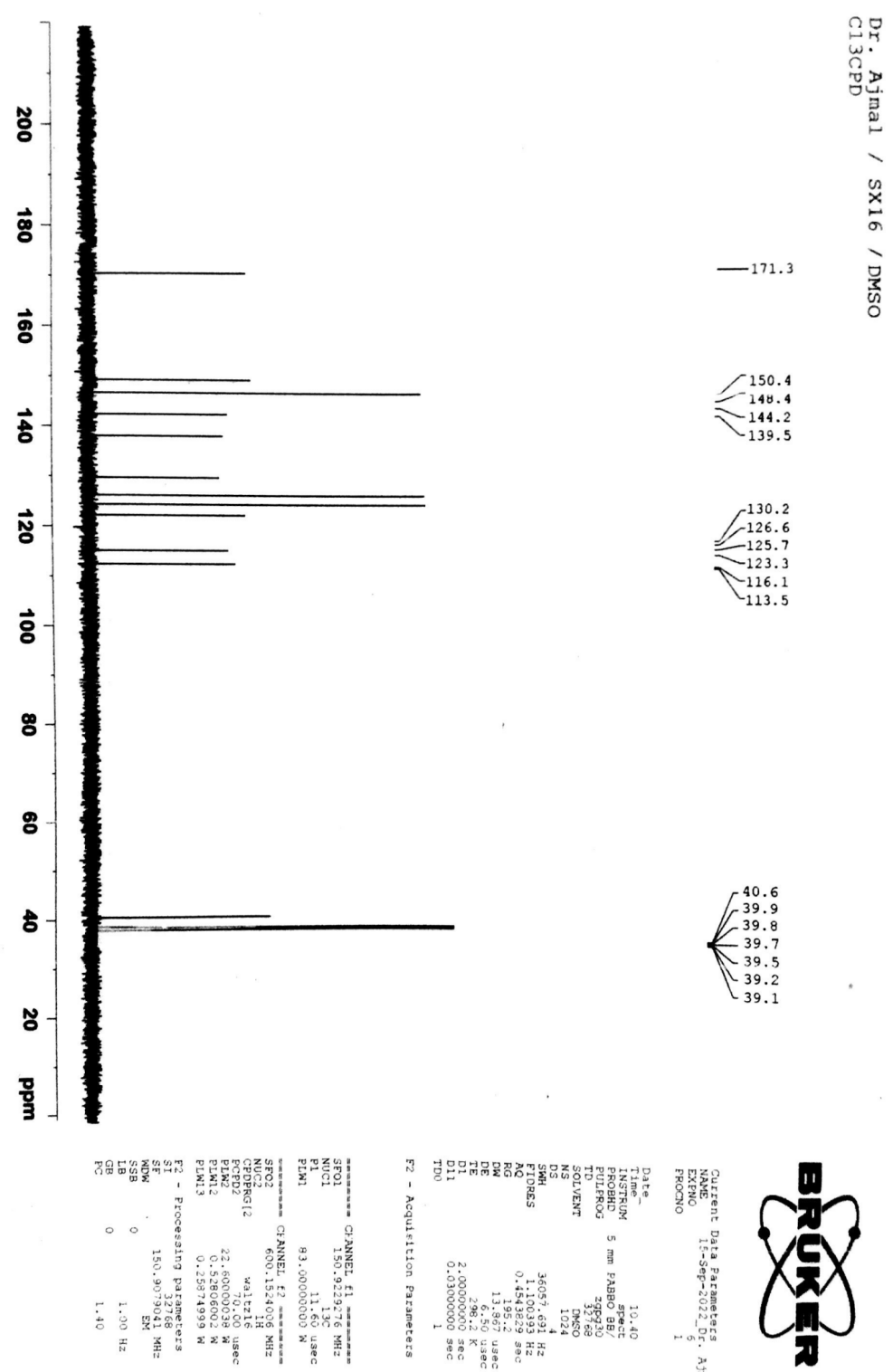


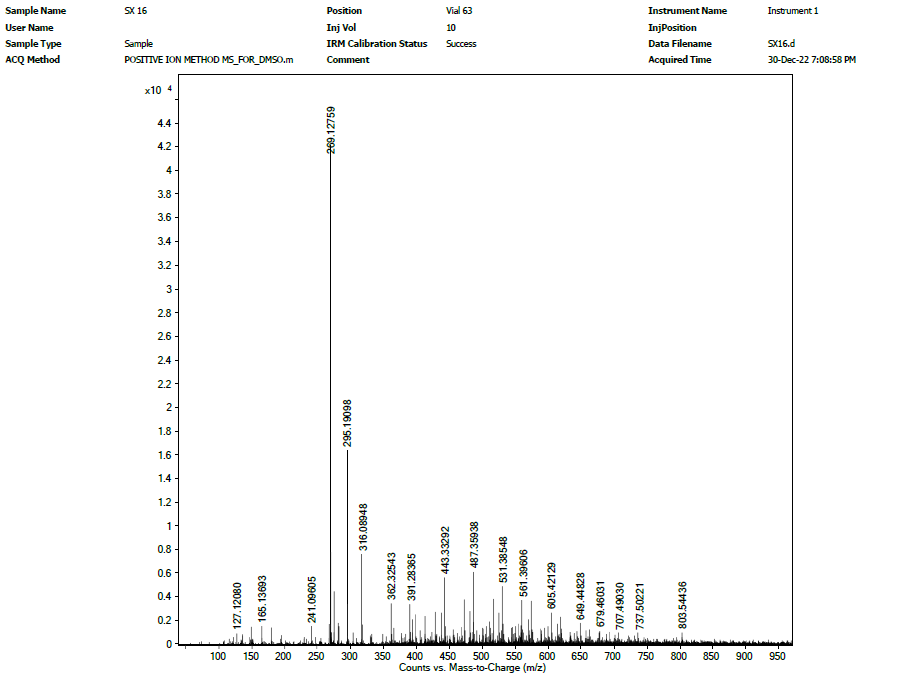
 **Fig S24:** ^1^H-, ^13^C-NMR and HR-ESI-MS spectra of compound **24**


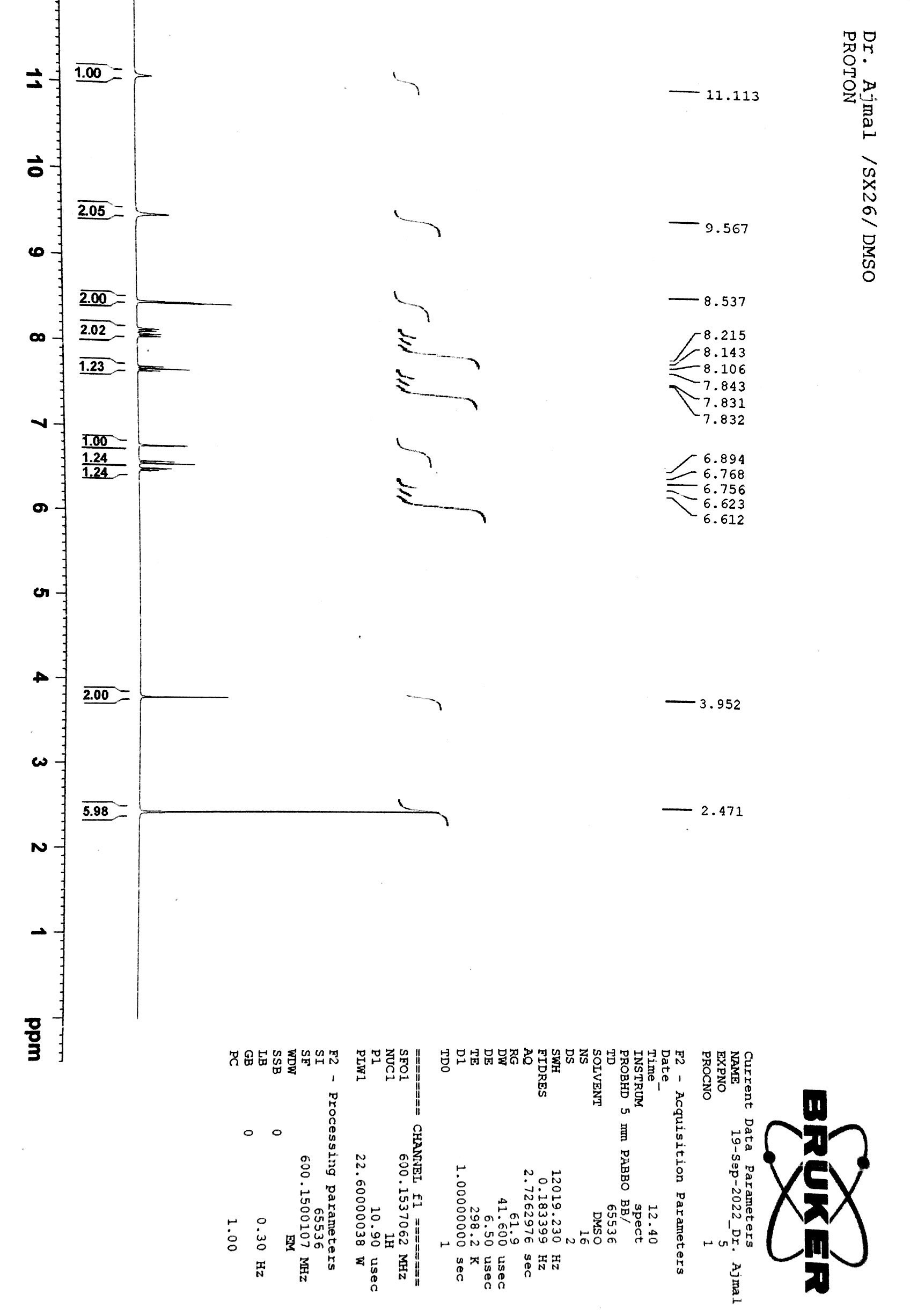


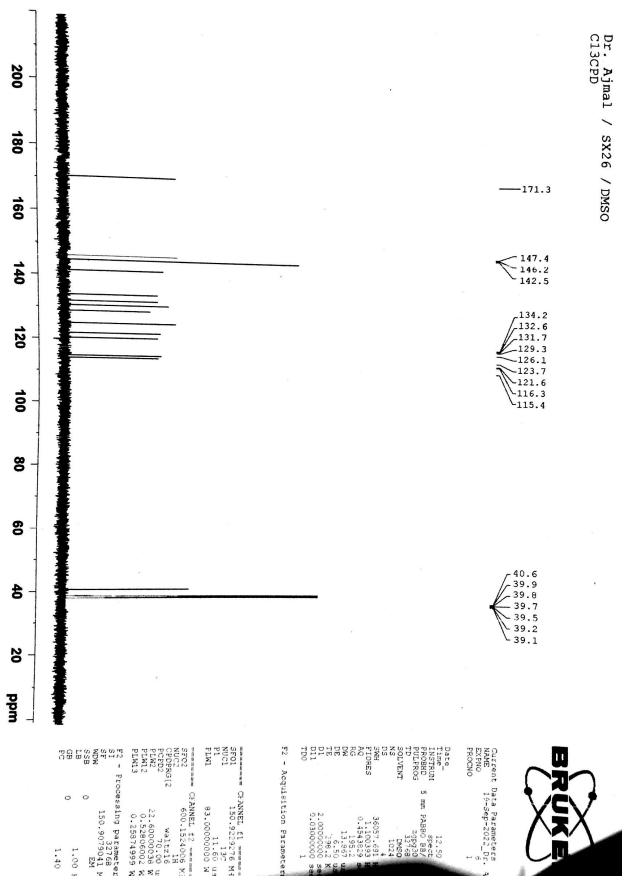


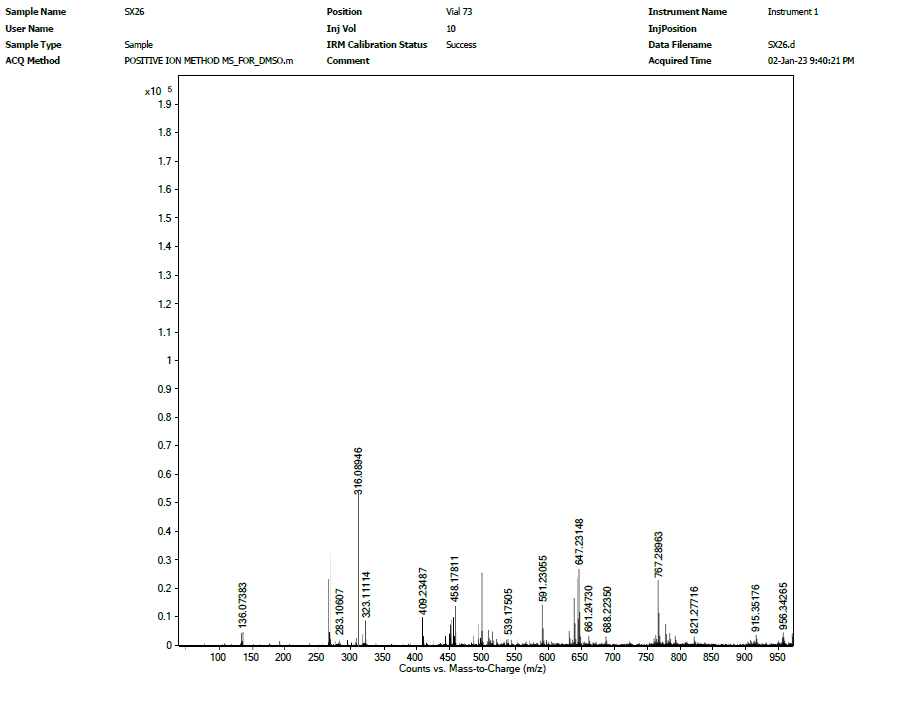


**Fig S25:** ^1^H-, ^13^C-NMR and HR-ESI-MS spectra of compound **25**


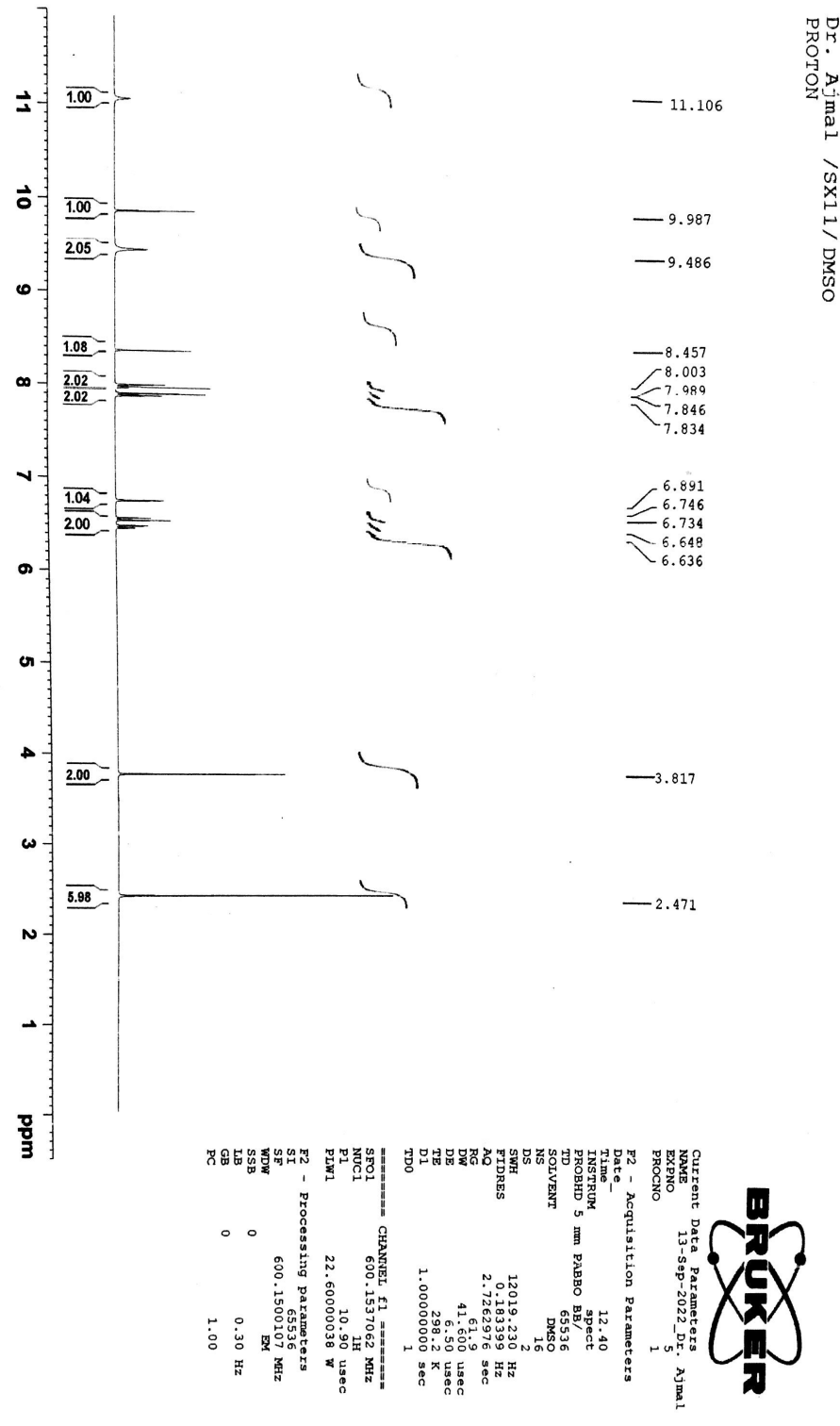


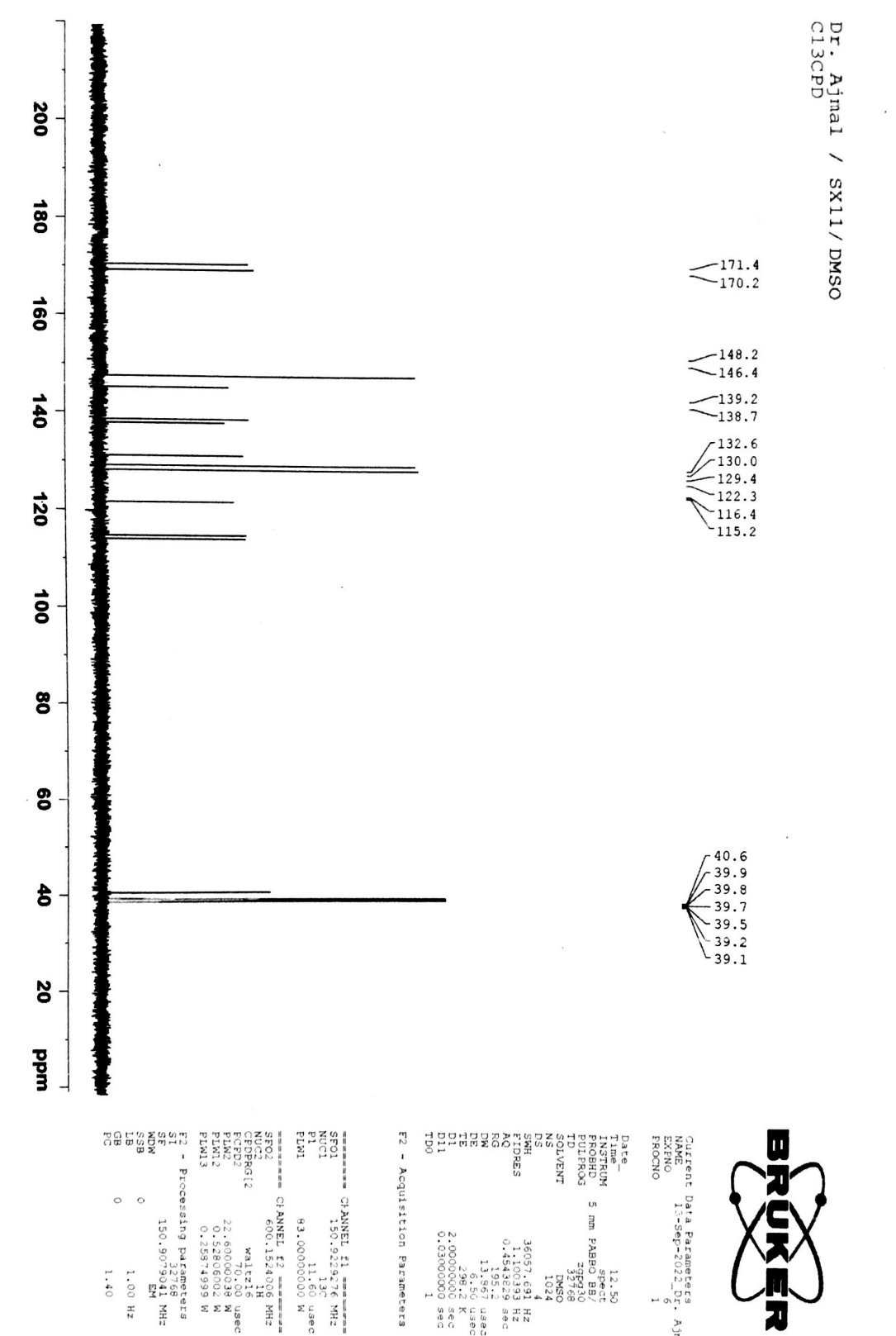


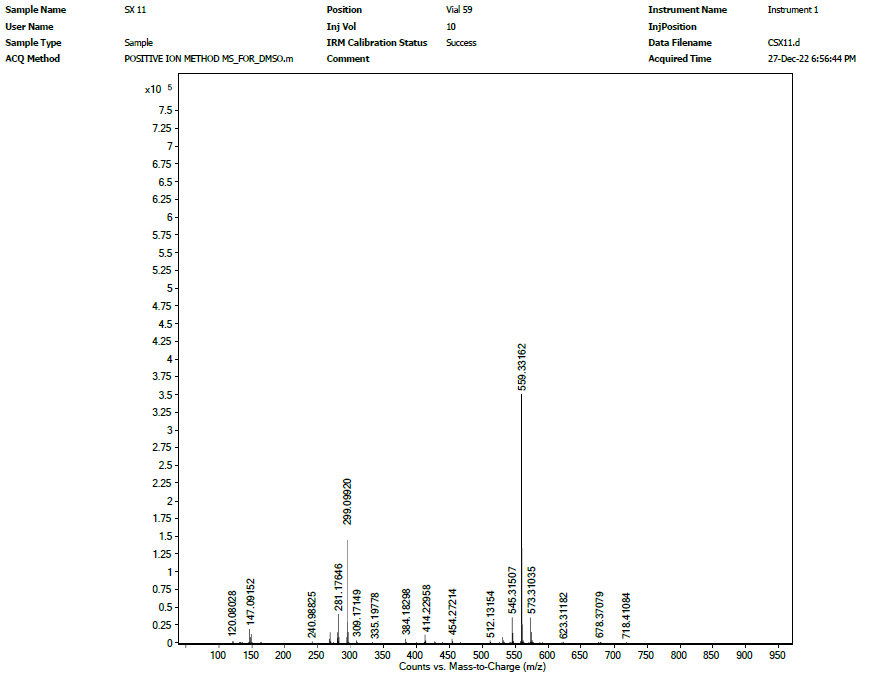


**Fig S26:** ^1^H-, ^13^C-NMR and HR-ESI-MS spectra of compound **26**


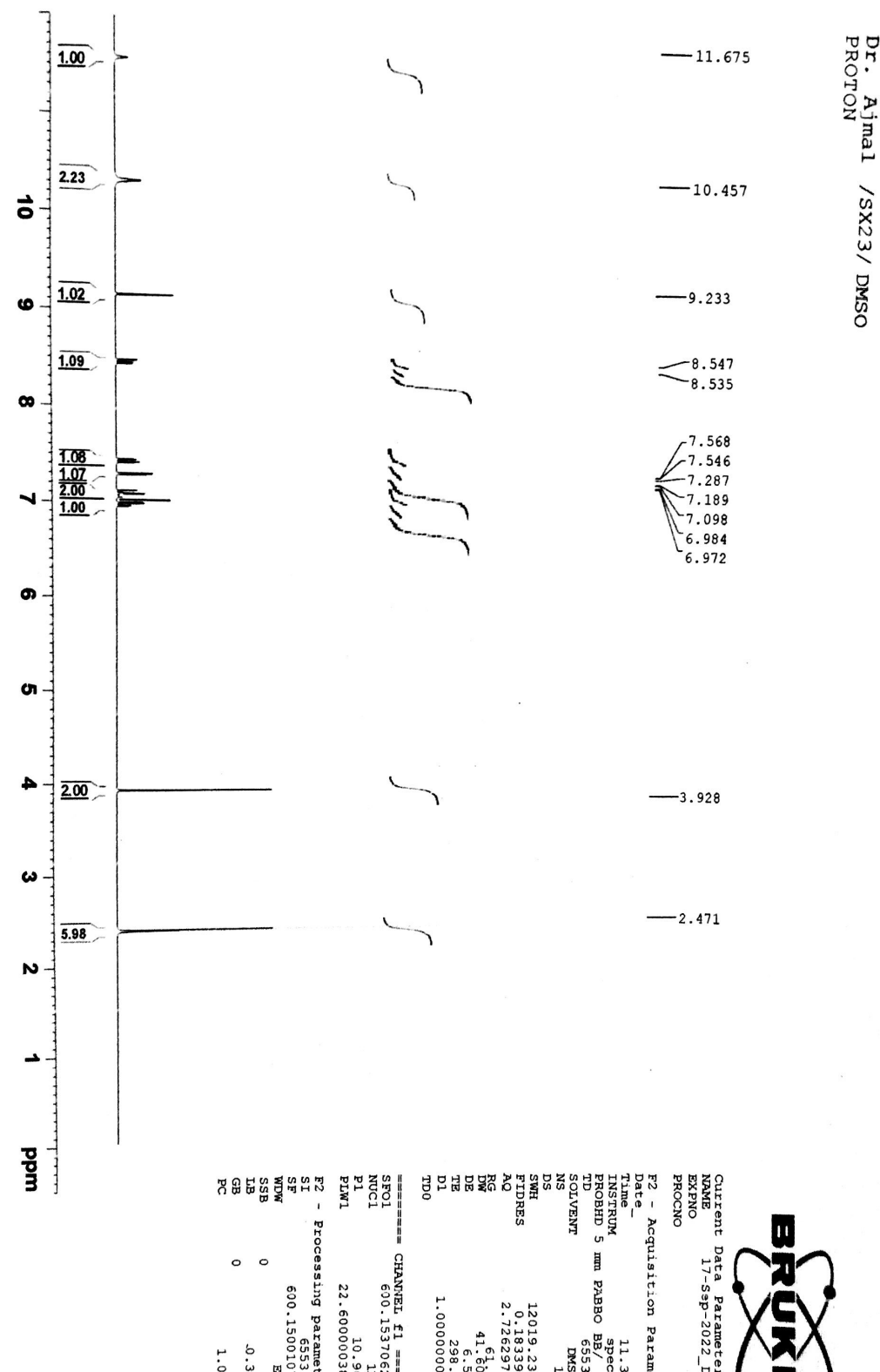


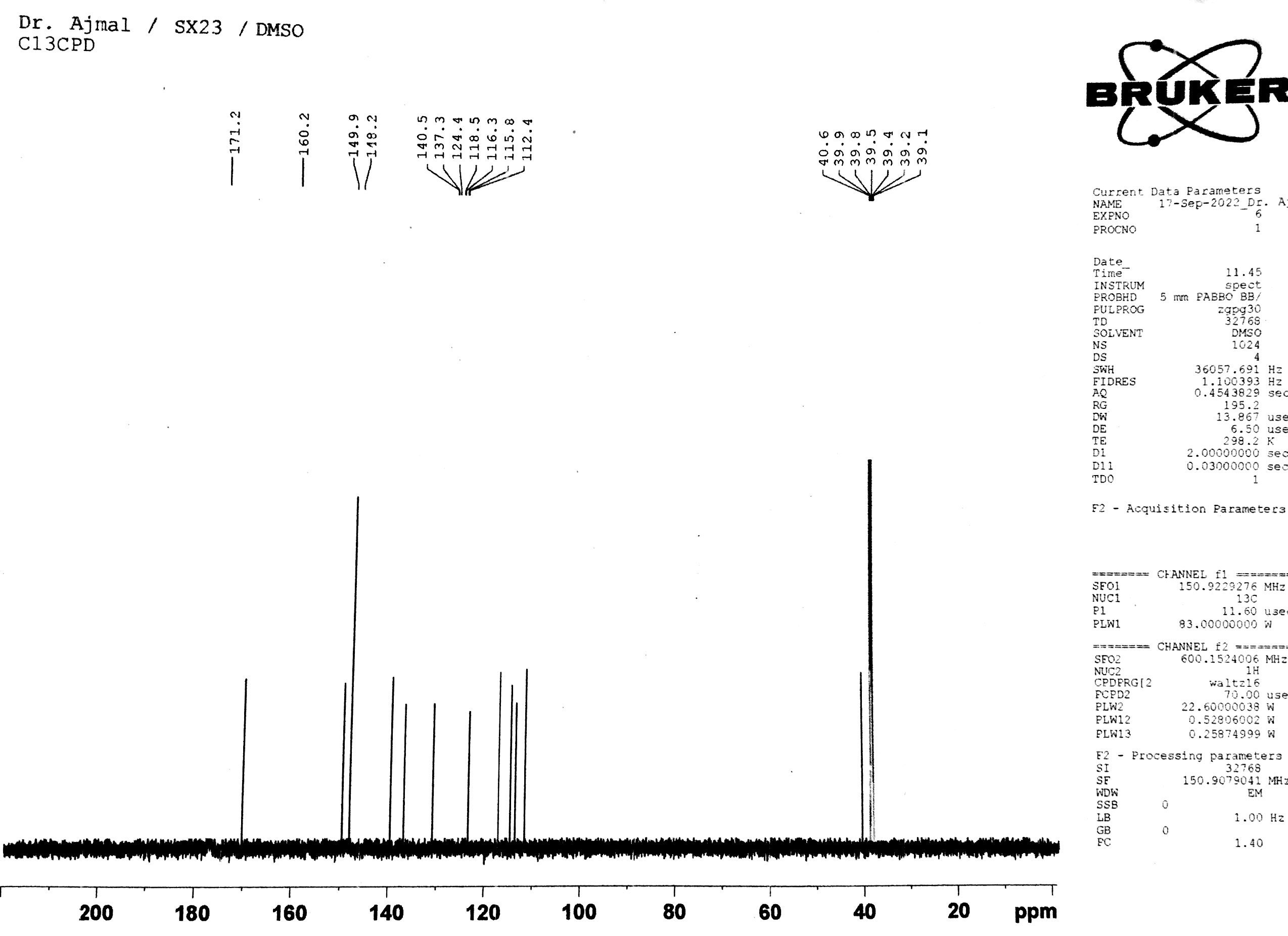


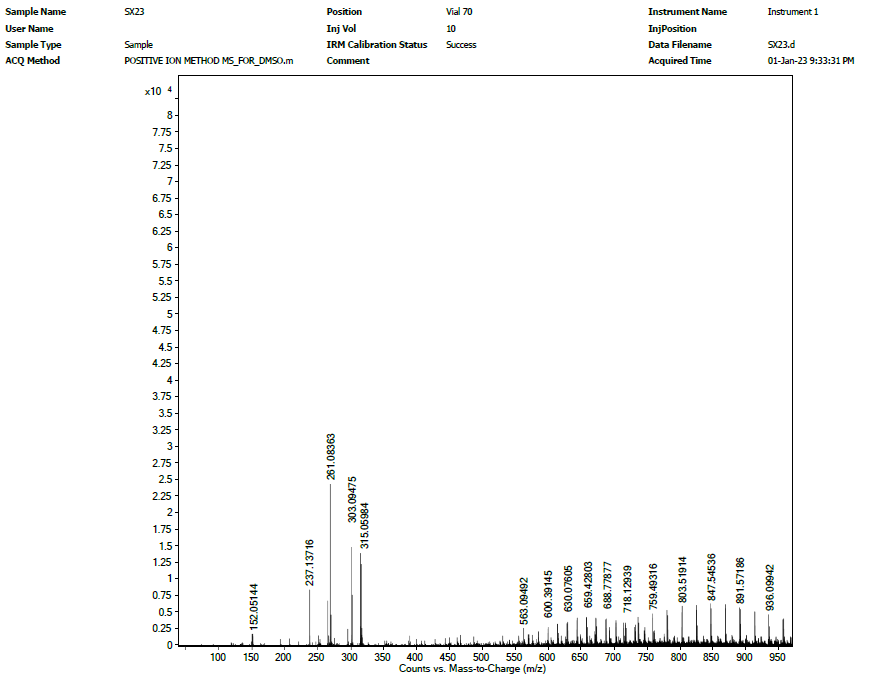


**Fig S27:** ^1^H-, ^13^C-NMR and HR-ESI-MS spectra of compound **27**


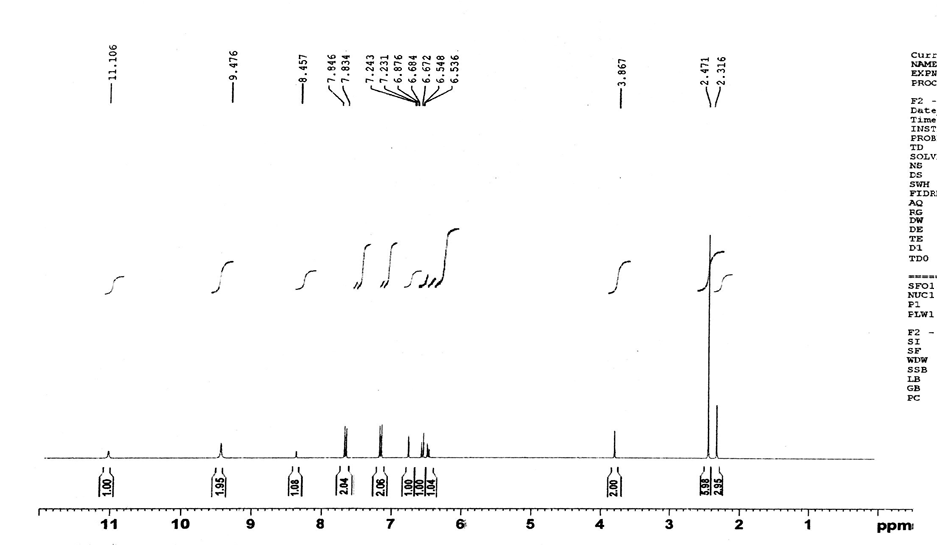


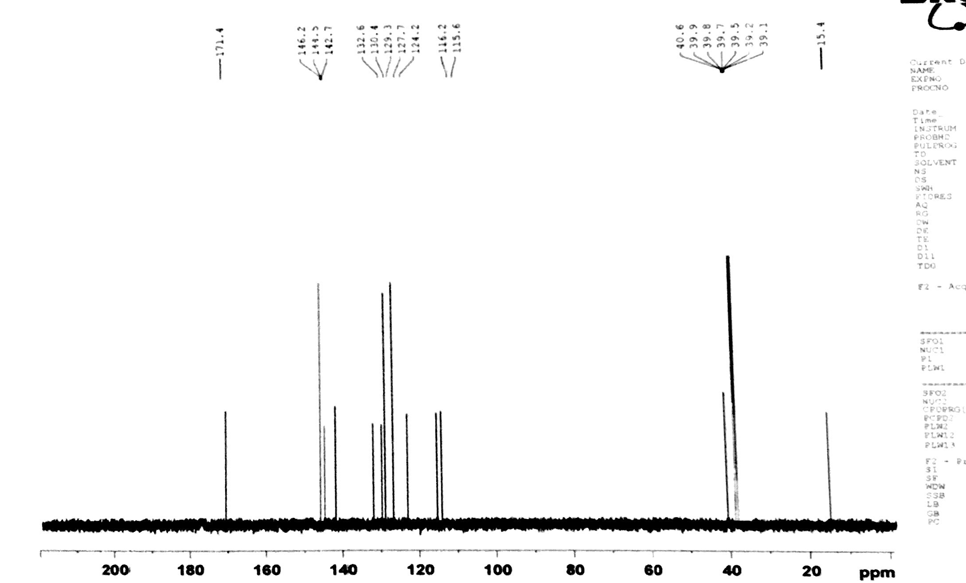


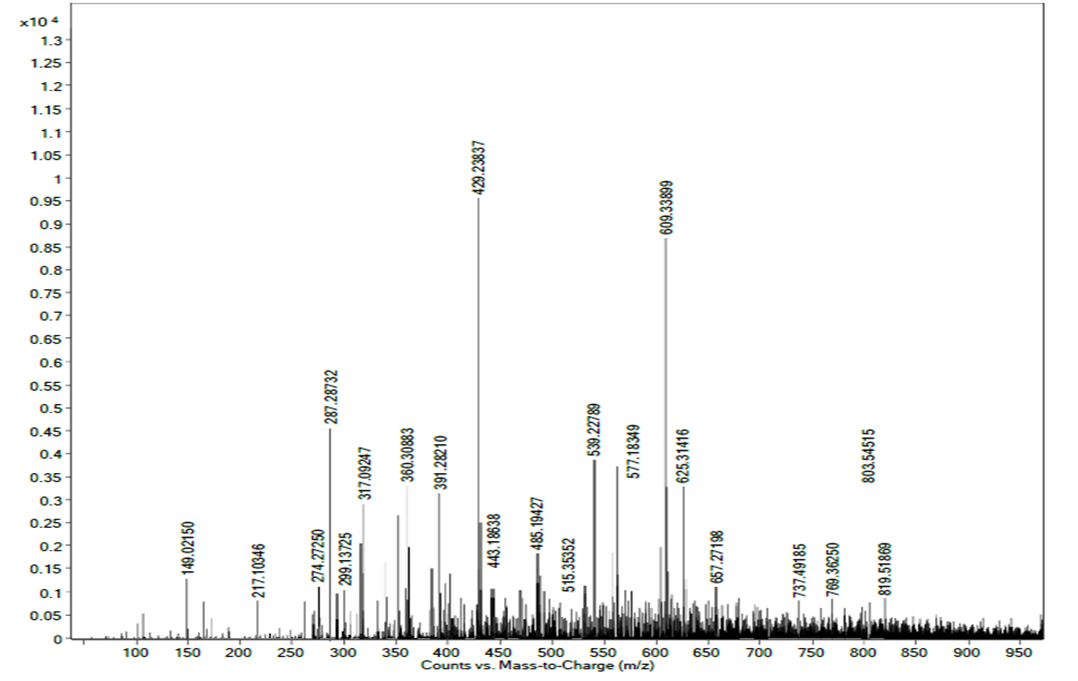


**Fig S28:** ^1^H-, ^13^C-NMR and HR-ESI-MS spectra of compound **28**

**Protocol for the α-glucosidase inhibition assay**

The α-glucosidase inhibition activity was performed with slight modifications as given by Rahim et al. Total volume of 100 μL reaction mixture contained, 70 μL 50mM phosphate buffer pH 6.8, 10μL (0.5mM in methanol) test compound, followed by the addition of 10μL (0.057 units, Sigma Inc.) enzyme solution in the buffer. The contents were mixed, pre-incubated for 10 min at 37 °C and pre-read at 400 nm. The reaction was initiated by the addition of 10 μL of 0.5mM substrate(p-nitrophenyl glucopyranoside, Sigma Inc.). After 30 min of incubation at 37 °C, the absorbance of p-nitrophenol was measured at 400 nm using the Synergy HT 96-well plate reader, BioTek, USA. Acarbose was used as positive control. All experiments were carried out in triplicates (mean ± SEM, n=3). Percent inhibition was calculated by the following equation: Inhibition (%) = (Abs of Control−Abs of Test/Abs of Control) × 100 Active compound solutions were suitably diluted, and their inhibition studies were determined. Data obtained was used for the determination of IC50 values (concentration at which there is 50% enzyme inhibition) using EZ-Fit Enzyme Kinetics Software (PerrellaScientificInc. Amherst, USA).

**Raw data for the α-glucosidase inhibition assay**

|  |  |  | **Exp 1-5** |  |  |  |  |  |  |  |  |  |
| --- | --- | --- | --- | --- | --- | --- | --- | --- | --- | --- | --- | --- |
| Result Data | |  |  |  |  |  |  |  |  |  |  |  |
|  | 1 | 2 | 3 | 4 | 5 | 6 | 7 | 8 | 9 | 10 | 11 | 12 |
| A | 2.501726 | 2.00273 | 1.793763 | 4.210274 | 4.30235 | 4.72534 | 7.8253 | 8.62735 | 9.02736 | 11.2837 | 11.8965 | 12.5347 |
| B | 2.6712 | 2.3846 | 2.7354 | 5.7344 | 6.1226 | 6.20165 | 11.234 | 10.0287 | 11.02737 | 16.6528 | 15.57294 | 17.62543 |
| C | 4.67321 | 6.3848 | 5.3374 | 7.34618 | 8.102355 | 7.632274 | 13.1028 | 12.78355 | 12.12762 | 3.41873 | 3.62937 | 3.8711 |
| D | 9.374746 | 8.363546 | 9.654242 | 12.4866 | 11.3645 | 11.60263 | 16.83551 | 17.346 | 17.87354 | 6.2366 | 6.401828 | 5.89236 |
| E | 10.4726 | 10.6524 | 11.03635 | 20.346 | 19.36524 | 18.28365 | 2.102763 | 2.63543 | 3.102874 | 10.238 | 9.563827 | 9.749287 |
| F | 13.23631 | 12.83467 | 13.00264 | 2.294515 | 1.902635 | 2.001726 | 6.017262 | 5.4827 | 4.762552 | 11.456 | 12.0388 | 12.4038 |
| G | 15.623 | 14.60274 | 16.31028 | 4.283645 | 4.49353 | 5.001244 | 6.582726 | 7.450283 | 8.102827 | 16.8636 | 18.237 | 17.73535 |
| H | 3.674881 | 2.6124 | 2.301655 | 6.5237 | 7.201827 | 6.004531 | 8.502663 | 9.1376 | 9.623356 | 25.65012 | 24.85012 | 25.00001 |

|  |  |  | **Exp 6-11** |  |  |  |  |  |  |  |  |  |
| --- | --- | --- | --- | --- | --- | --- | --- | --- | --- | --- | --- | --- |
| Result Data | |  |  |  |  |  |  |  |  |  |  |  |
|  | 1 | 2 | 3 | 4 | 5 | 6 | 7 | 8 | 9 | 10 | 11 | 12 |
| A | 2.501726 | 2.00273 | 2.101828 | 7.3487 | 7.803837 | 8.302784 | 9.238 | 10.408 | 11.0023 | 3.724 | 3.87 | 4.5128 |
| B | 2.4712 | 2.43846 | 2.57354 | 10.2339 | 10.523 | 9.73652 | 13.10233 | 13.847 | 14.29341 | 6.20377 | 5.8376 | 6.8364 |
| C | 4.567321 | 6.333848 | 5.63374 | 12.23 | 11.4562 | 10.83636 | 2.102763 | 2.63543 | 2.6033 | 8.10727 | 7.3825 | 8.407333 |
| D | 9.212337 | 8.213635 | 9.541654 | 15.73 | 16.89274 | 16.386 | 4.196 | 3.765 | 3 | 9.3082 | 10.00274 | 10.6923 |
| E | 10.74726 | 10.49652 | 11.10364 | 1.80266 | 1.5637 | 2.37 | 6.017262 | 5.4827 | 4.762552 | 11.487 | 10.836 | 11.02837 |
| F | 14.6432 | 15.0213 | 15.621 | 4.283645 | 4.49353 | 5.001244 | 8.31055 | 7.3065 | 6.418 | 16.69274 | 17.2038 | 19.827 |
| G | 3.12938 | 3.84 | 3.6012 | 6.5237 | 7.201827 | 6.004531 | 8.502663 | 9.1376 | 9.623356 | 16.8636 | 17.27452 | 17.73535 |
| H | 4.82736 | 5.30828 | 5.6833 | 7.87 | 8.56672 | 7.203847 | 14.7 | 16.2 | 15.61 | 25.65012 | 26.85012 | 25.76 |
|  |  |  |  |  |  |  |  |  |  |  |  |  |
|  | Result Data |  | **Exp 12-17** |  |  |  |  |  |  |  |  |  |
|  | 1 | 2 | 3 | 4 | 5 | 6 | 7 | 8 | 9 | 10 | 11 | 12 |
| A | 2.401727 | 1.63826 | 1.827602 | 4.622 | 5.10227 | 5.51286 | 10.60282 | 10.20374 | 9.963534 | 18.8926 | 17.42838 | 18.20277 |
| B | 3.63512 | 4.10273 | 4.302365 | 6.3017 | 6.74928 | 7.20188 | 11.69822 | 11.02838 | 11.29365 | 6.20377 | 5.8376 | 5.2018 |
| C | 6.238162 | 6.109274 | 5.745256 | 9.2034 | 8.6963 | 9.583344 | 14.20177 | 15.4822 | 15.79264 | 8.10727 | 7.3825 | 8.407333 |
| D | 9.21288 | 8.836442 | 9.601277 | 10.72543 | 10.36242 | 11.00233 | 3.81625 | 3.502727 | 3 | 9.3082 | 8.7362 | 9.692535 |
| E | 11.03938 | 10.49236 | 10.82039 | 14.48477 | 15.23937 | 14.022 | 6.102 | 5.3826 | 4.59626 | 10.40283 | 9.592635 | 11.1028 |
| F | 12.0287 | 11.69264 | 11.90374 | 3.201727 | 2.791625 | 1.793636 | 8.002837 | 7.420182 | 6.10736 | 14.62424 | 15.10287 | 16.502 |
| G | 17.82534 | 15.61227 | 16.73027 | 6.23017 | 6.655749 | 7.311029 | 9.501727 | 9.019837 | 8.593636 | 25.4 | 27.001 | 26.5028 |
| H | 2.82736 | 1.8026 | 2.735441 | 9.002728 | 8.472525 | 9.391717 | 10.60283 | 11.10374 | 10.47826 | 25.2365 | 26.85012 | 26.876 |
|  |  |  |  |  |  |  |  |  |  |  |  |  |
|  |  |  | **Exp 18-23** |  |  |  |  |  |  |  |  |  |
| Result Data | |  |  |  |  |  |  |  |  |  |  |  |
|  | 1 | 2 | 3 | 4 | 5 | 6 | 7 | 8 | 9 | 10 | 11 | 12 |
| A | 2.470173 | 2.1297 | 1.7634 | 7.30172 | 6.8344 | 7.592725 | 19.865 | 20.672 | 21.328 | 2.401827 | 2.736524 | 3.01827 |
| B | 3.6132 | 3.2076 | 3.489155 | 10.38625 | 10.72543 | 9.8533 | 1.68235 | 2.40283 | 1.401277 | 4.19278 | 4.59277 | 4.8623 |
| C | 5.293663 | 5.63087 | 4.89355 | 12.3017 | 11.49273 | 11.63737 | 3.293641 | 3.62535 | 4.10746 | 6.82652 | 7.02736 | 7.420187 |
| D | 9.374746 | 8.363546 | 9.654242 | 4.725 | 4.301626 | 4.59625 | 5.1028 | 4.792534 | 5.482635 | 8.397161 | 9.018236 | 8.753133 |
| E | 10.3922 | 10.349 | 11.30274 | 6.23751 | 6.72534 | 5.81625 | 7.20377 | 6.592635 | 7.502635 | 10.238 | 11.3736 | 10.79364 |
| F | 15.623 | 14.60274 | 16.31028 | 7.8265 | 8.102737 | 7.39262 | 9.381653 | 10.4762 | 9.692636 | 17.632 | 17.2037 | 19.8354 |
| G | 3.674881 | 4.012 | 4.59235 | 9.4017 | 10.736 | 9.69236 | 15.735 | 14.26142 | 16.8254 | 16.8636 | 15.3521 | 17.73535 |
| H | 5.3092 | 6.20176 | 5.792534 | 11.4846 | 12.30475 | 11.45828 | 1.4652 | 2.02837 | 1.20133 | 27.87 | 27.5102 | 28.1028 |
|  |  |  |  |  |  |  |  |  |  |  |  |  |
|  |  |  | **Exp 24-33** |  |  |  |  |  |  |  |  |  |
| Result Data | |  |  |  |  |  |  |  |  |  |  |  |
|  | 1 | 2 | 3 | 4 | 5 | 6 | 7 | 8 | 9 | 10 | 11 | 12 |
| A | 2.861424 | 2.89365 | 3.70836 | 7.230172 | 6.798834 | 7.659273 | 10.3653 | 11.36356 | 10.62038 | 17.229 | 15.3928 | 18.236 |
| B | 4.28176 | 3.871524 | 4.59625 | 10.32863 | 10.57254 | 9.78533 | 14.4927 | 14.827 | 15.3028 | 0.388 | 1.2939 | 0.938 |
| C | 6.490228 | 6.71028 | 6.39 | 12.3017 | 11.49273 | 11.63737 | 4.0012 | 5.2038 | 4.410293 | 0.0038 | 0.383737 | 0.01828 |
| D | 9.374746 | 8.363546 | 9.654242 | 14.902 | 14.387 | 15.62535 | 6.2366 | 7.1028 | 6.428 | 0.01828 | 0.01828 | 0.3838 |
| E | 10.23922 | 10.2349 | 11.23027 | 1.63 | 2.10287 | 2.455633 | 7.20377 | 6.592635 | 7.502635 | 0.3838 | 0.3838 | 0.28387 |
| F | 18.402 | 16.823 | 17.69264 | 3.28622 | 3.610287 | 4.1029 | 9.481653 | 10.64762 | 9.892636 | 0.283837 | 0.283837 | 0.38873 |
| G | 3.102827 | 2.3018 | 2.401726 | 5.3018 | 5.742029 | 6.20187 | 11.393 | 12.00188 | 11.29374 | 0.38873 | 0.38873 | 0.3737 |
| H | 5.3092 | 4.201374 | 5.792534 | 8.39176 | 8.591726 | 8.82635 | 16.203 | 16.82535 | 18.3028 | 26.10236 | 26.8 | 27 |

| Compd | Dose Curve response | Percent Inhibition (0.5mM) | **IC_50_ ± µM (SEM)** |
| --- | --- | --- | --- |
| 1 | 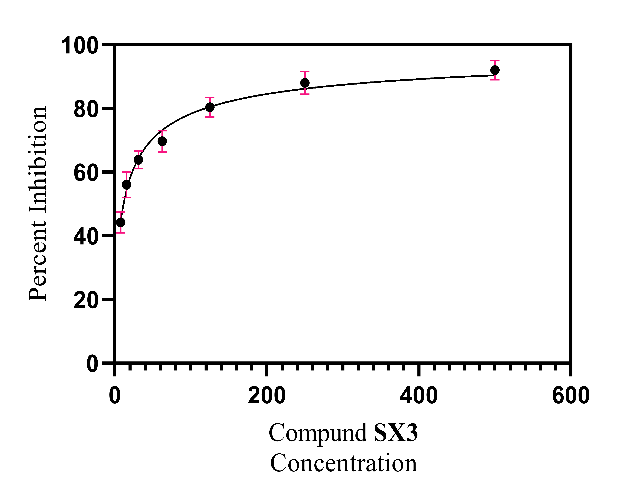 | 86.33 | 24.37 ± 0.83 |
| 2 | 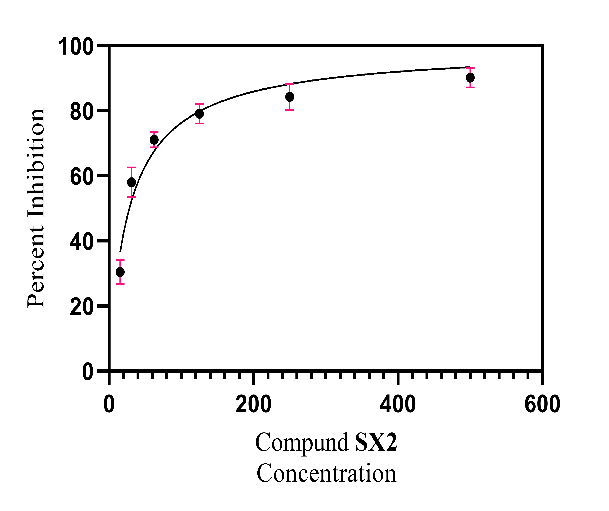 | 90.43 | 20.35 ± 1.27 |
| 3 | 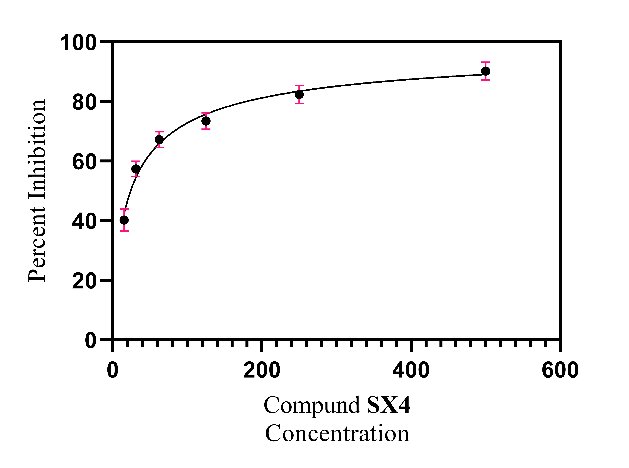 | 91.33 | 18.45 ± 1.21 |
| 4 | 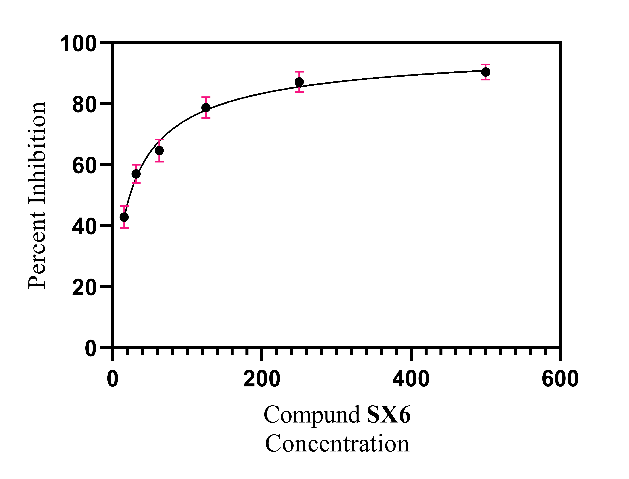 | 95.71 | 13.64 ± 0.58 |
| 5 | 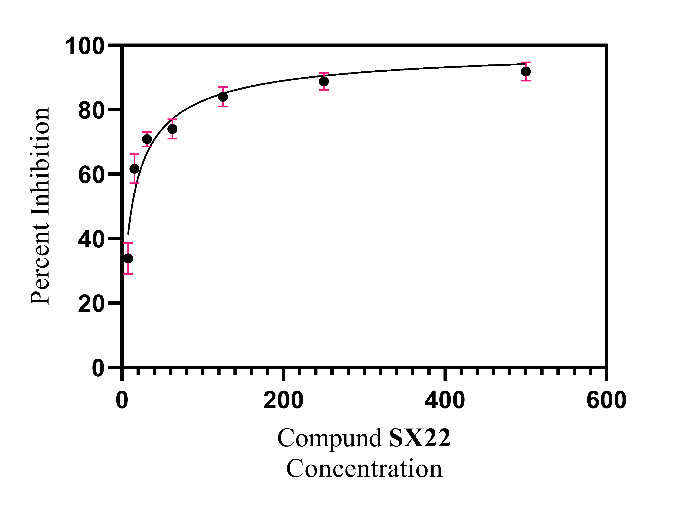 | 96.12 | 12.84 ± 0.52 |
| 6 | 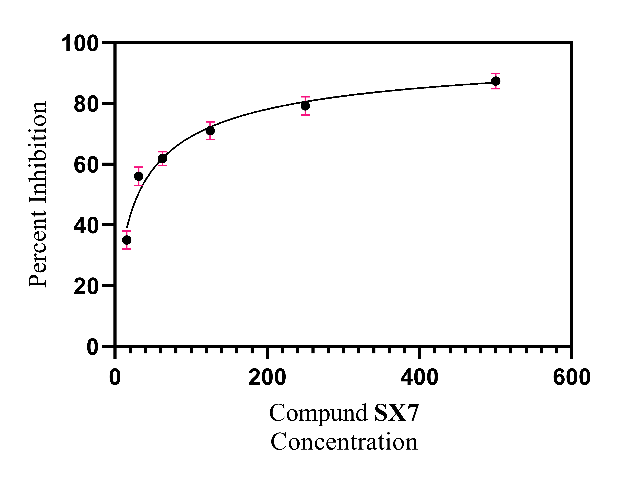 | 86.42 | 23.69 ± 1.11 |
| 7 | 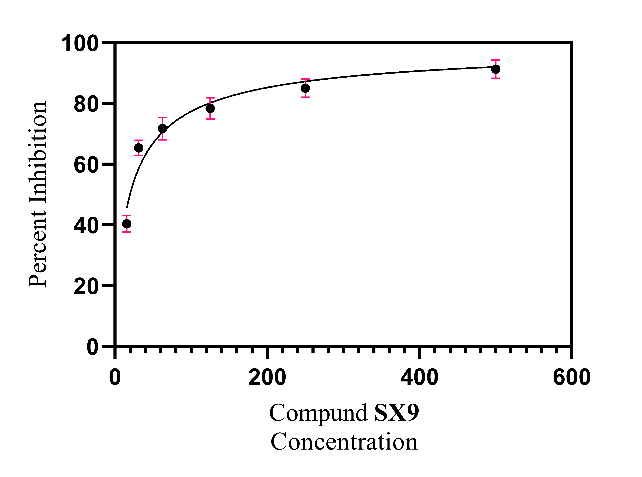 | 91.73 | 19.68 ± 0.82 |
| 8 | 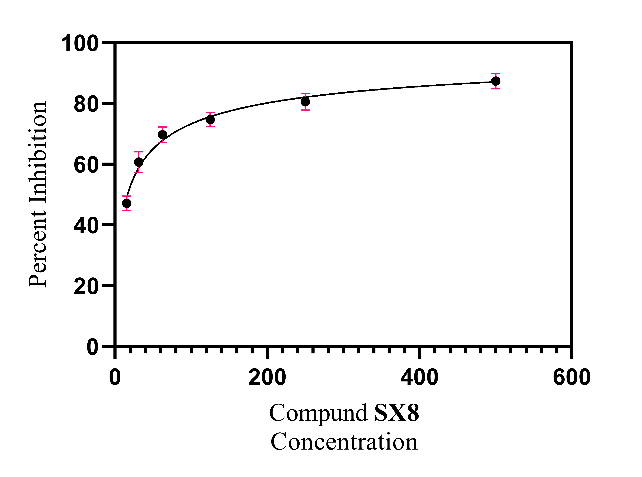 | 90.98 | 20.88 ± 0.53 |
| 9 | 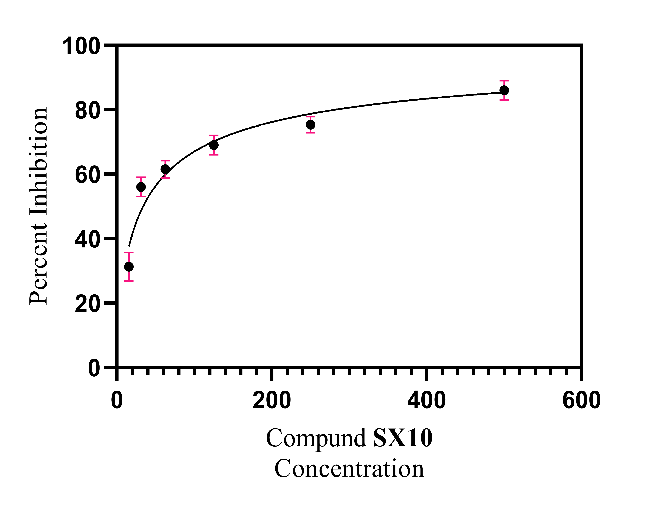 | 83.30 | 27.29 ± 1.15 |
| 10 | 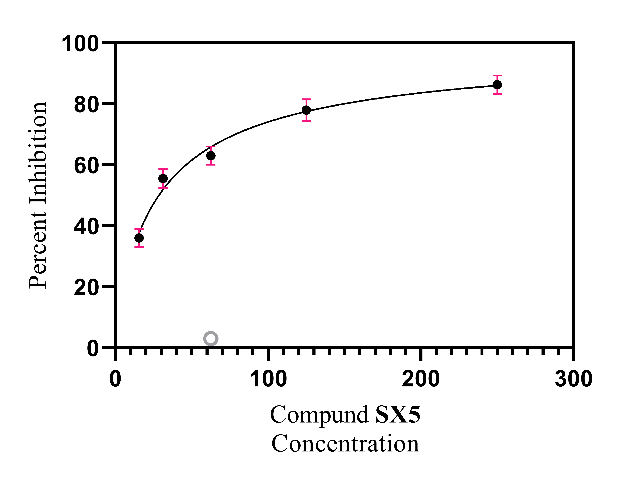 | 81.91 | 28.66 ± 1.21 |
| 11 | 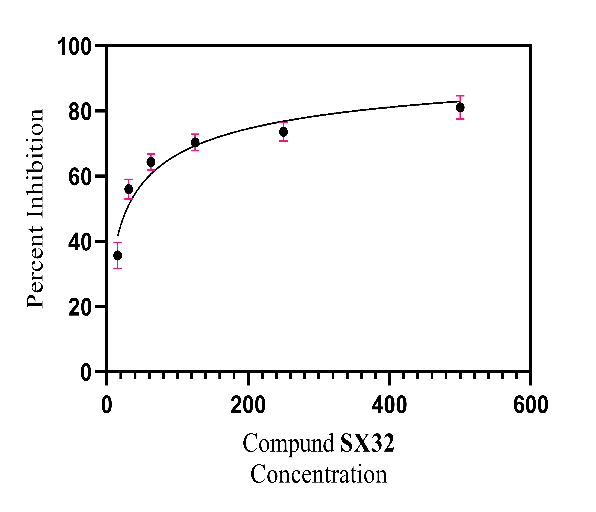 | 77.47 | 33.51 ± 1.22 |
| 12 | 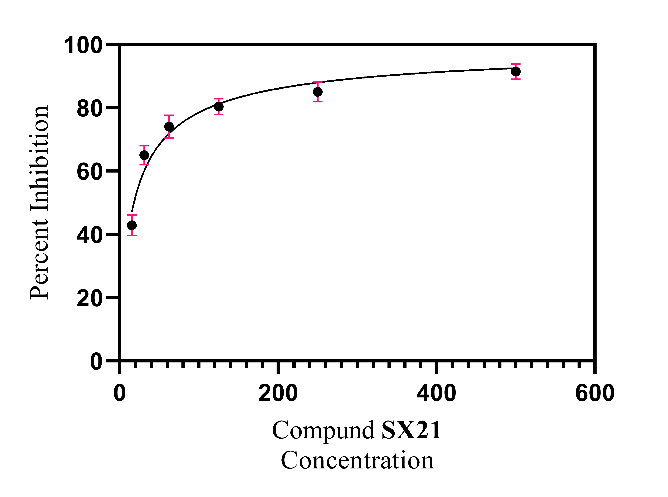 | 96.68 | 15.73 ± 0.71 |
| 13 | 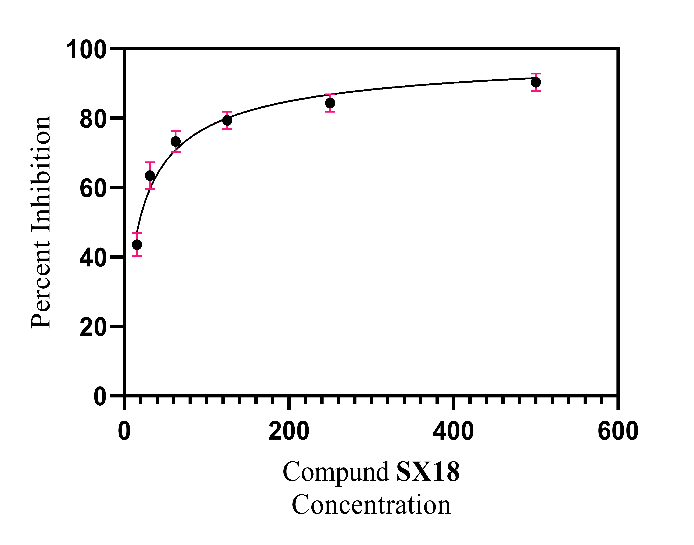 | 95.25 | 16.62 ± 0.47 |
| 14 | 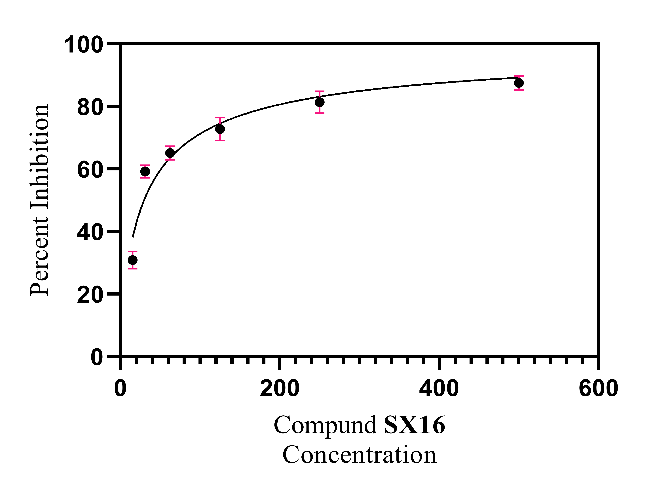 | 92.16 | 20.61 ± 0.59 |
| 15 | 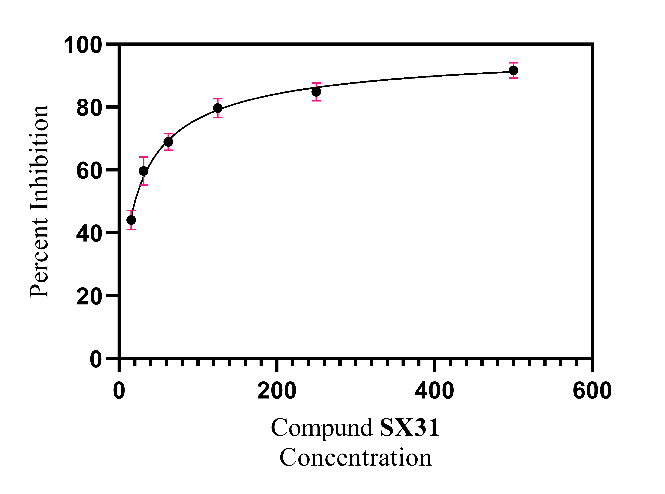 | 92.76 | 17.405 ± 0.74 |
| 16 | 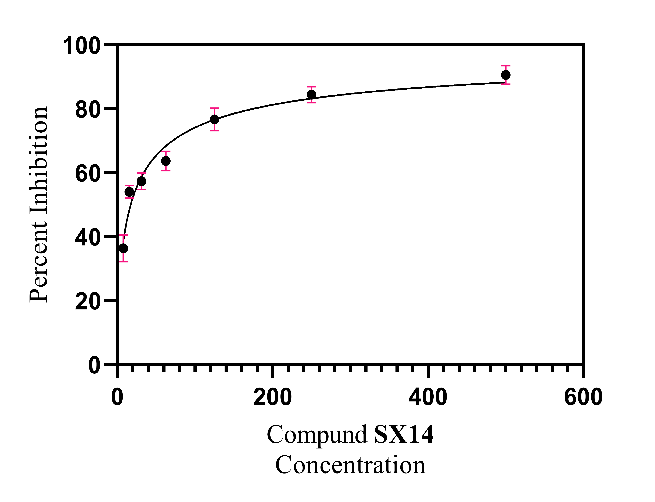 | 84.12 | 26.41 ± 1.10 |
| 17 | 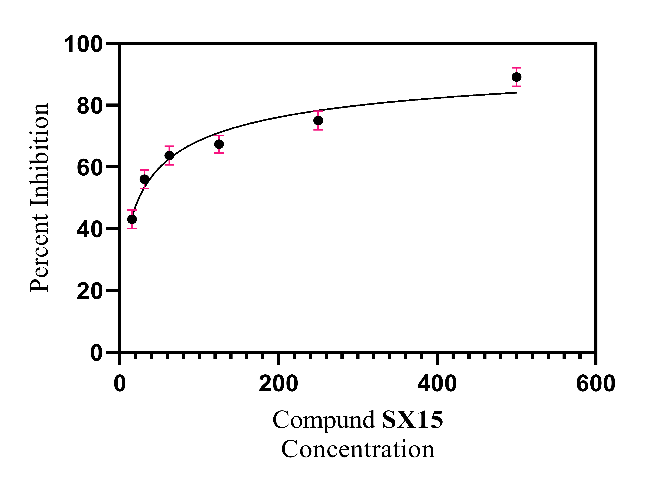 | 71.97 | 43.76±2.34 |
| 18 |  | 85.30 | 28.16 ± 1.09 |
| 19 |  | 79.43 | 31.22 ± 1.40 |
| 20 |  | 84.40 | 27.26 ± 0.83 |
| 21 |  | 86.42 | 24.16 ± 0.52 |
| 22 |  |  | N/A |
| 23 |  | 80.90 | 30.46 ± 1.28 |
| 24 |  | 82.71 | 29.67 ± 0.98 |
| 25 |  | 78.21 | 34.46 ± 1.48 |
| 26 |  |  | N/A |
| 27 |  |  | N/A |
| 28 |  |  | N/A |
| **Standard Acarbose** | | **59.37** | **873.34 ± 1.67** |

**α-Glucosidase Inhibition Assay Raw data table with dose Curve and Percent Inhibition**
